# Supplementary figures and images for: Effectiveness of couple education and counseling on knowledge, attitude and uptake of cervical cancer screening service among women of child bearing age in Southern Ethiopia: A cluster randomized trial protocol
Source: PLoS One. 2022 Jul 21;17(7):e0270663. doi: 10.1371/journal.pone.0270663 (PMC9302843; doi:10.1371/journal.pone.0270663)

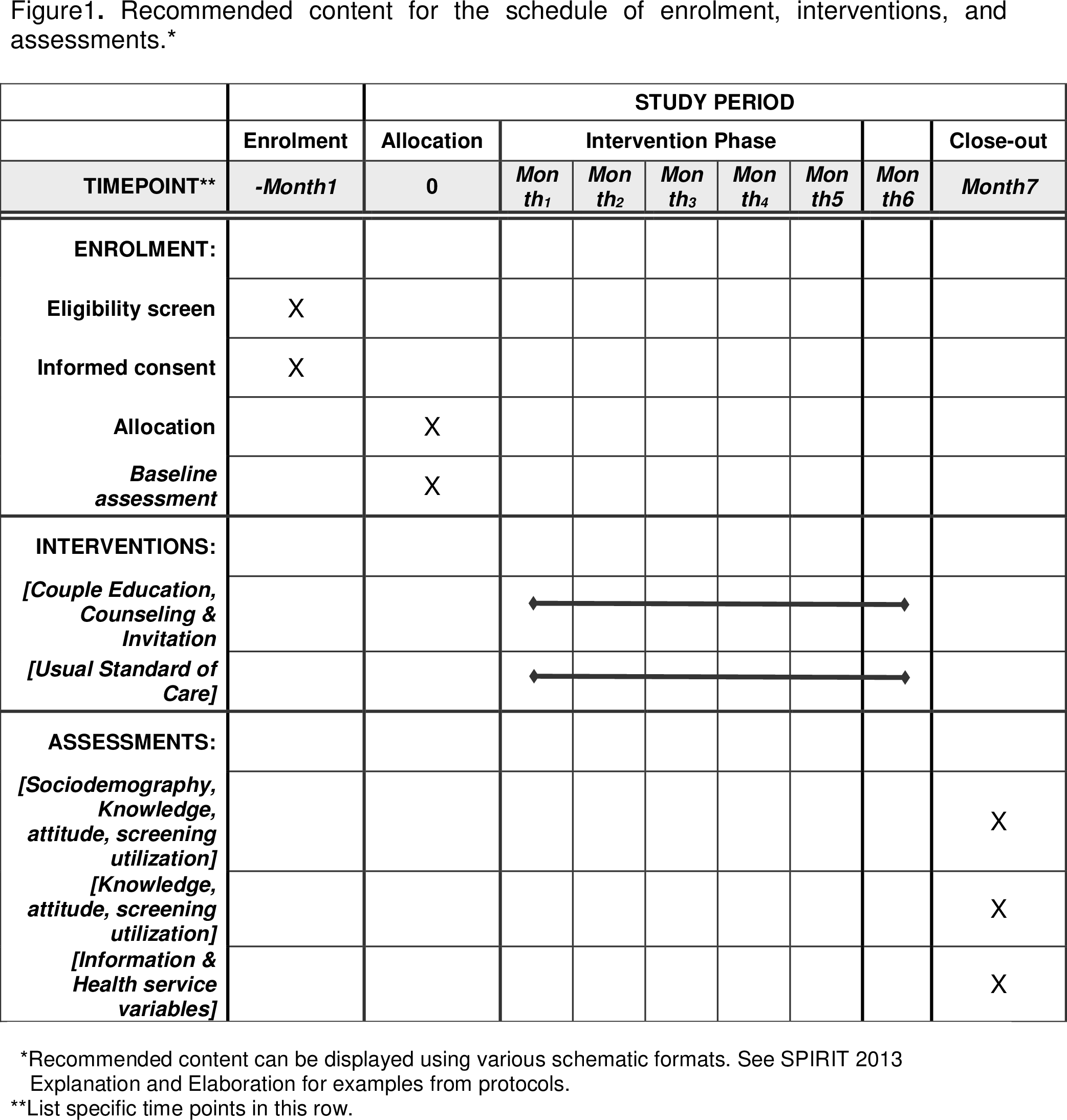

Supplement: S1 Fig — (TIF) [file pone.0270663.s002.tif]
