# Supplementary material for: Effectiveness of couple education and counseling on knowledge, attitude and uptake of cervical cancer screening service among women of child bearing age in Southern Ethiopia: A cluster randomized trial protocol
Source: PLoS One. 2022 Jul 21;17(7):e0270663. doi: 10.1371/journal.pone.0270663 (PMC9302843; doi:10.1371/journal.pone.0270663)
Supplement: S1 Appendix — (DOCX) [file pone.0270663.s003.docx]

**Appendices**

Annex I: Information sheet and informed consent form

Dear Respondent:

My name is ____________. I am working for the research project entitled “*effect of couple education on knowledge, attitude and uptake of cervical cancer screening service among women of child bearing age in southern Ethiopia*” as a data collector. The purpose of the study is to determine the effect of couple education on knowledge, attitude and uptake of cervical cancer screening services among women of child bearing age. Participation in this research will benefit individual woman by increasing her knowledge and encourage behavioral change to utilize the screening services. It also gives an opportunity to ask questions and learn more about the topic confidentially after the interview. Currently I am working with the research team of Jimma University to realize such objectives. Now, I am going to interview you & collect information which is required for the research purposes. You are randomly selected to be included in the study as part of the sample population to complete the questionnaire designed by the research team. The information obtained in this study will be used only for research purposes. The information you will provide is helpful to achieve the intended objectives of the study so that you and your community will benefit from. Any information obtained will be kept strictly confidential and will not be exposed to any other body. No personal identifiers will be attached to the information you will provide. Involvement in this study is voluntary and you can drop any individual question or the whole questionnaire at any time without giving a reason. Your refusal to participate or drop out will not produce penalty. However, your participation and contribution in the study is very helpful to come up with important findings to intervene the problem. The questionnaire will probably take 30-40 minutes to complete. For any information you can contact Mr. Samuel Yohannes; phone ______

Do you have any opinion regarding this study? Do you agree to participate in this study?

Yes, continue No, thank you!

**Consent form**

I, the undersigned have been informed, in the language I can understand, and understood the purpose of this particular research project. I have been informed that the information I give will be used only for the purpose of the study; my identity, the information I give will be treated confidentially. I have also been informed that I can refuse to participate in the study, not to respond to question if I am not interested or stop responding to question at any time in the process. Based on the above information I agree to participate in the research voluntarily.

Participant’s Sign____________ Person in charge of the informed consent, sign _____________
