# Supplementary material for: Effectiveness of couple education and counseling on knowledge, attitude and uptake of cervical cancer screening service among women of child bearing age in Southern Ethiopia: A cluster randomized trial protocol
Source: PLoS One. 2022 Jul 21;17(7):e0270663. doi: 10.1371/journal.pone.0270663 (PMC9302843; doi:10.1371/journal.pone.0270663)
Supplement: S1 Protocol — (DOCX) [file pone.0270663.s004.docx]

CERVICAL CANCER SCREENING IN ETHIOPIA: EFFECT OF COUPLE EDUCATION ON KNOWLEDGE, ATTITUDE AND UPTAKE OF CERVICAL CANCER SCREENING SERVICE AMONG WOMEN OF CHILD BEARING AGE IN SOUTHERN ETHIOPIA

BY: SAMUEL YOHANNES (BSc, MPH/RH, PhD FELLOW)

A PhD RESEARCH PROPOSAL SUBMITTED TO THE SCHOOL OF GRADUATE STUDIES OF JIMMA UNIVERSITY FOR THE FULFILLMENT OF THE REQUIREMENTS FOR THE DEGREE OF DOCTOR OF PHILOSOPHY (PhD) IN REPRODUCTIVE HEALTH

JULY 2021,

JIMMA UNIVERSITY

Cervical Cancer Screening In Ethiopia: Effect of Couple Education on Knowledge, Attitude and Uptake of Cervical Cancer Screening Service among Women of Child Bearing Age in Southern Ethiopia

BY: *Samuel Yohannes (BSc, MPH/RH, PhD Fellow)*

ADVISORS: - *Muluemebet Abera Wordofa (BSc, MPH/RH, PhD, Associate Professor)*

*Tefera Belachew Lema (MD, MSc, DLSHTM, PhD, Professor)*

A PhD Research Proposal Submitted to the School of Graduate Studies of Jimma University for the Fulfillment of the Requirements for the Degree of Doctor of Philosophy (Phd) in Reproductive Health

July 2021,

Jimma University

# DECLARATION FORM

Letter for Declaration (Dissertation proposal work)

I, the under signed, declared that this is my bona fide original work, has never been presented in this or any other University, and that all the resources and materials used for the thesis, have been fully acknowledged.

Name: *Samuel Yohannes Ayanto*

Signature: _________________________________________

Date: __________________________________________

Place: Jimma University, Jimma Ethiopia

Date of submission: _____________________________________

This dissertation proposal has been submitted for examination with my approval as Candidate’s Promoter (supervisor).

Name: *Muluemebet Abera Wordofa* Signature: ____________ Date: _________

Co-promoter (Co supervisor)

Name: *Tefera Belachew Lema* Signature: ____________ Date: ___________

Team Leader, Dep’t of Population & Family Health Director, Research Office, IOH, JU

Name: ____________________ Name: ______________________

Signature: ________________ Signature: ________________

Date: ____________________ Date: ____________________

# TABLE OF CONTENTS

[DECLARATION FORM iii](#_Toc78807283)

[TABLE OF CONTENTS iv](#_Toc78807284)

[LIST OF TABLES v](#_Toc78807285)

[LIST OF FIGURES vi](#_Toc78807286)

[LIST OF APPENDICES vii](#_Toc78807287)

[ABBREVIATIONS viii](#_Toc78807288)

[SUMMARY x](#_Toc78807289)

[INTRODUCTION 1](#_Toc78807290)

[Statement of the problem 3](#_Toc78807291)

[LITERATURE REVIEW 11](#_Toc78807292)

[CONCEPTUAL FRAMEWORK 20](#_Toc78807293)

[SIGNIFICANCE OF THE STUDY 21](#_Toc78807294)

[RESEARCH QUESTIONS 22](#_Toc78807295)

[AIMS OF THE THESIS /GENERAL OBJECTIVES 23](#_Toc78807296)

[RESEARCH METHODS AND MATERIALS 24](#_Toc78807297)

[A SUMMARY (BRIEF OUTLINES) OF THE DISSERTATION WORK 36](#_Toc78807298)

[BUDGET BREAK DOWN 37](#_Toc78807299)

[RESEARCH WORK PLAN 39](#_Toc78807300)

[REFERENCES 40](#_Toc78807301)

[ANNEXES 49](#_Toc78807302)

# LIST OF TABLES

[Table 1 Summary of the intervention modality 29](#_Toc79402897)

[Table 2 Brief summary of the dissertation work 36](#_Toc79402898)

[Table 3 Detailed budget breakdown 37](#_Toc79402899)

[Table 4 Research work plan and timelines 39](#_Toc79402900)

# LIST OF FIGURES

[Figure 1 Conceptual frame work of the study developed from the literature review 20](#_Toc78807358)

[Figure 2 Map of Kembata Tembaro Zone; Source Zonal Health Department 24](file:///C:\Users\admin\Desktop\PhD%20Research%20Project%20Documents%202020\1_PhD%20Research%20Project%20Proposal%202020\1_Research%20Project%20Proposal\PhD%20Project%20Version_7_Final\PhD%20Research%20Project%20Proposal_Final.docx#_Toc78807359)

[Figure 3 the mechanism how the health belief model works 29](#_Toc78807360)

[Figure 4 Implementation frame work for the intervention project 30](#_Toc78807361)

[Figure 5 Sampling frame for the intervention study 32](#_Toc78807362)

# LIST OF APPENDICES

[Annex 1Participant’s Information and informed consent agreement form /English/ 49](#_Toc78807380)

[Annex 2 Questionnaire for cluster randomized controlled trial 50](#_Toc78807381)

[Annex 3 Questionnaire for the case control study **Error! Bookmark not defined.**](#_Toc78807382)

[Annex 4 Qualitative Research Interview Guide **Error! Bookmark not defined.**](#_Toc78807383)

[Annex 5 Health Education Brochure /English Version/ 55](#_Toc78807384)

[Annex 6 CV of Principal Investigator 65](#_Toc78807385)

[Annex 7 CV of Advisors 68](#_Toc78807386)

# ABBREVIATIONS

AORTIC African Organization for Research and Training in Cancer

ASIR Age Specific Incidence Rate

ASMR Age Specific Mortality Rate

BCC Behavior Change Communication

CC Cervical Cancer

CCS Cervical Cancer Screening

CIN Cervical Intraepithelial Neoplasia

CIS Carcinoma In Situ

cm Centimeter

CRCT Cluster Randomized Control Trial

CRT Cluster Randomized Trial

DNA Deoxyribonucleic Acid

Dr. Doctor

ECHO Extension for Community Healthcare Outcomes

FGA Family Guidance Association

FGDs Focus Group Discussion

FMOH Federal Ministry of Health

GATHER Greeet, Ask, Tell, Help, Explain, Return

GLOBOCAN Global Cancer Observatory

HAART Highly Active Anti-Retroviral Therapy

HBM Health Belief Model

HDI human development index

HIV Human Immune Deficiency Virus

HP Health Professionals

HPV Human Papilloma Virus

hrHPV High Risk Human Papilloma Virus

ICC Invasive Cervical Cancer

IUD Intra Uterine Device

kg Kilogram

KI Key Informants

LHA Lay Health Advisors

LMICs Low- and Middle-Income Countries

NCCP National Cancer Control Plan

NCD Non-Communicable Disease

OR Odds Ratio

RCT Randomized Controlled Trial

SMS Short Message Service

SNNPR Southern Nations Nationalities and Peoples' Regional State

sq. km Square Kilo Meter

SRS Simple Random Sampling

SSA Sub-Saharan Africa

STD Sexually Transmitted Diseases

STI Sexually Transmitted Infection

TASH Tikur Anbesa Specialized hospital

US United States

VIA Visual Inspection with Acetic Acid

WCBA Women of Child Bearing Age

WHO World Health Organization

# SUMMARY

**Background**

Majority of Human Papilloma Virus infections resolve naturally, but progress to precancerous lesions whenever there is persistent infection. When treatment is delayed, the precancerous lesions progress to the cancerous stage. In 2018, cervical cancer occurred nearly in 570 000 women and 311 000 women died of the disease worldwide. Nearly 84% of all cervical cancer cases and 88% of all deaths due to cervical cancer took place in resource-limited nations. Majority of the cases over 80% in sub-Saharan Africa including Ethiopia are detected at a late stage mainly due to lack of information & effective measures at early stage exacerbated by poor uptake of available screening services. Implementation of effective screening programs aimed at increasing screening uptake to early detect and treat precancerous changes can lead to a significant reduction in the morbidity & mortality associated with advanced disease.

**Objectives**

The objectives of this study are to identify the determinants of cervical cancer screening uptake and evaluate the effect of couple education on knowledge, attitude and uptake of cervical cancer screening services among eligible women of child bearing age in Southern Ethiopia.

**Methods**

In this intervention study, we will randomly assign clusters in to two groups, the intervention arm and control arm. Health education will be given to the intervention group, but the control group receives standard of care. Base line and end line surveys will be completed by 288 eligible women to evaluate the effect of the health education on the knowledge, attitude and screening uptake between the two arms & the results are compared.

**Expected outcomes**

The effect of education intervention on the knowledge, attitude & screening services uptake will be evaluated.

**Work plan**

The research project will be implemented from June 2021 to December 2021

**Budget**

The total budget for the research project will be **208026.00 Ethiopian Birr**

# INTRODUCTION

**Background**

Human Papilloma Virus (HPV) belongs to a group of deoxyribonucleic acid (DNA) viruses that mainly affects epithelial cells of the body (Stanley, Lowy and Frazer, 2006). It is one of the most common causes of sexually transmitted infections in the world (Scheurer, Tortolero-Luna and Adler-Storthz, 2005). There are over 170 known HPV genotypes worldwide (Ghittoni *et al.*, 2015). Fifteen of the known HPV genotypes are mainly recognized as high-risk HPV types (hr-HPV) which are responsible for the development of HPV-related cancers (Haedicke and Iftner, 2013). More than 4% of all types of cancer cases worldwide occur due to infection by high risk HPV strains. It accounts almost 2% of all cancer cases in developed nations and nearly 8% of all cancer cases in low income countries (Parkin, 2006). The most common strains of HPV in Africa are HPV 16 and 18 which are identified to cause over 65% of cervical cancer cases in the SSA (Bruni *et al.*, 2010).

Human Papilloma Virus (HPV) is the necessary cause for the development of cervical cancer which is mainly transmitted through sexual intercourse. Majority of HPV infections resolve naturally, but it progresses to precancerous lesions whenever there is persistent infection. When treatment is delayed beyond the precancerous stage, the lesions progress to advanced cancerous stage. The precancerous lesion usually takes 10 to 20 years to advance in to invasive cervical cancer. This, in majority of the cases, gives a good opportunity to prevent the development of advanced cervical cancer through early detection and treatment of precancerous lesions (WHO, 2014).

In 2018, cervical cancer occurred nearly in 570 000 women and 311 000 women died from the disease worldwide. Globally, cervical cancer was the fourth most common cancer and the fourth leading cause of cancer death among women. Nearly 84% of all cervical cancer cases and 88% of all deaths due to cervical cancer took place in resource-limited nations. But in resource-rich countries the incidence and mortality due to cervical cancer were two to four times lower than what had been seen in resource scarce countries. The highest disease burden was demonstrated in southern and eastern Africa (Arbyn *et al.*, 2020).

In its incidence and mortality rates, cervical cancer ranks the second after breast cancer in lower human development index (HDI) regions of the world. Larger proportions of cervical cancer cases and deaths occurred in Sub-Saharan African countries. Of the highest regional incidence and mortality rates observed in Africa, Eastern Africa shared the highest mortality rate due to cervical cancer in 2018 (Bray *et al.*, 2018). Also it has been reported that the highest prevalence of cervical infection with HPV is recorded in Sub-Saharan Africa (SSA) countries (De Vuyst *et al.*, 2013). According to the projections made by World Health Organization (WHO), cervical cancer will be responsible for 443,000 deaths in 2030 globally (WHO, 2012). Of these deaths, 98% will occur in low income countries, with SSA facing the highest number of deaths (Ferlay *et al.*, 2015).

Cervical cancer incidence and mortality have been considerably reduced in high resource countries during the last few decades. This is mainly due to the implementation of screening packages for the detection of precancerous cervical lesions and HPV. The availability of improved treatment options also played their role in this regard (Denny *et al.*, 2017) . However, in low- and middle income countries where access to screening and treatment measures is limited, cervical cancer remains a significant public health problem (Bray *et al.*, 2018). In Ethiopia, nearly 6,300 new cases are identified, and about 4,884 women die from cervical cancer each year. This makes cervical cancer the second-most common, and the second-most deadly cancer in the country among women (Bruni *et al.*, 2019).

# Statement of the problem

**Global context**

In 2008 Global Cancer Observatory (GLOBOCAN) estimated that nearly 530 000 cases and 275 000 deaths were caused by cervical cancer worldwide. The less developed world accounted 85% of the cancer burden in the year. The global age standardized incidence rate and mortality rate were estimated to be 15 and 8/100, 000 women respectively. Cervical cancer ranked the third in magnitude among cancers and the fourth in causing cancer death globally. Women in developing countries experienced 86% of all cases and 88% of all deaths caused by cervical cancer worldwide. In developing countries, the age specific incidence rate (ASIR) and age specific mortality rate (ASMR) were 18 and 10 per 100 000, respectively, whereas in well developed countries it is 9 and 3 per 100 000, respectively (Arbyn *et al.*, 2011).

In 2012, the global cancer statistics showed that there were an estimated 527,600 new cervical cancer cases and 265,700 deaths worldwide. It was identified to be the second most common cancer and third leading cause of cancer death among women in less developed nations (Torre *et al.*, 2015). Cervical cancer in low- and middle-income countries (LMICs) accounted for approximately 85% of new cases diagnosed globally in 2012. Also approximately 87% of the deaths from cervical cancer worldwide occurred in LMICs in the year (Ferlay *et al.*, 2015). All these statistics obviously describes that there have been disproportionately heavy burden of cervical cancer among women in less developed regions of the world. Women living in LMICs are increasingly at high risk of morbidity and mortality caused by cervical cancer which could be both preventable and treatable (WHO, 2014).

World Health organization (WHO) estimated that greater than 500,000 new cases and more than 270,000 deaths caused by cervical cancer occur each year worldwide which accounts 9% of all deaths due to female cancers (World Health Organization, 2013). Nearly 570, 000 women developed cervical cancer and 311, 000 women experienced cervical cancer deaths in 2018 worldwide. Globally it was the fourth most common cancer and the fourth major cause of cancer death among women in the year (Arbyn *et al.*, 2020; Bray *et al.*, 2018). Nearly 84% of all cervical cancers and 88% of all deaths caused by cervical cancer occurred in lower-resource countries but the incidence and mortality was two to four times lower in well developed countries (Arbyn *et al.*, 2020).

**Regional context**

In Sub-Saharan Africa (SSA), although incidence rates vary from country to country, approximately 93,225 new cases of cervical cancer occurred in 2012, making it the second most common cancer of reproductive-age women in the region (Black and Richmond, 2018). Southern and Eastern Africa share the highest burden of cervical cancer, where age-standardized incidence rates were 43.1 and 40.1 per 100,000, respectively (Bruni et al., 2019). In 2008, cervical cancer was identified as the most common cancer in Eastern Africa and the leading cause of cancer related deaths among women in sub-Saharan Africa (Arbyn et al., 2011). Also, in 2012, the highest incidence rates of cervical cancer were observed in sub-Saharan Africa (Torre et al., 2015).

In 2012, cervical cancer ranked the second in causing cancer morbidity among women in sub Saharan Africa which accounted 25.2% of all cancer cases following breast cancer (Harvey, Adeyoju and Brough, 2017). In 2018, the highest disease burden due to cervical cancer was observed in eastern and southern Africa. For example, the age specific incidence rates (ASIR) were the highest in countries from eastern, southern, or western Africa which was more than 40 per 100 000 women (Arbyn et al., 2020). In this year cervical cancer was the most commonly diagnosed cancer in 28 countries and the leading cause of cancer death in 42 countries, of which the vast majority are found in Sub-Saharan Africa and South- Eastern Asia. Eastern Africa is known to have the highest regional incidence and mortality rate (Bray et al., 2018). Cervical cancer is the most common cancer in women younger than age 50 years (Lou *et al.*, 2018).

**Epidemiology and risk factors**

Cross sectional studies have repeatedly reported that subclinical HPV infections are highly prevalent in young individuals, whereas invasive cervical cancer typically develops in the third decade and later. The cross sectional prevalence of HPV DNA decreases spontaneously to a background level of 2–8% in most populations in groups that are 40 years old and above. In countries where intensive screening of young women takes place, part of the HPV prevalence reduction could be attributable to aggressive treatment of HPV related cervical lesions. Women who remain chronic HPV carriers are currently described as the true high risk group for cervical cancer (Bosch *et al.*, 2001).

Women with high viral loads for HPV-16 had a 30 fold greater risk of developing cervical cancer than did HPV negative women. This also applies to women under the age of 25. A related article which used the same population showed that 20% of the population with the highest viral loads for HPV-16 had a 60 fold higher risk of developing carcinoma in situ when compared with HPV negative women (Josefsson *et al.*, 2000).

In a case control study conducted in Thiland significant difference in the risk of HPV was observed with respect to smoking exposure, age at first delivery (<18 years), age at first sexual intercourse (<16 years), prolonged use of injectable contraceptives (>2years), prolonged use of oral contraceptive pills (>2years), multiple sexual partners (>1), multiple pregnancies (>3) and multiple parities (>3). But smoking and HPV infection were significantly associated with the risk of cervical cancer. It also demonstrated that women who had sexual activity within 5 years of menarche were at increased risk for cervical cancer compared with those women who postponed it six years beyond menarche (Natphopsuk *et al.*, 2012).

In a crossectional study conducted in Spain different characteristics of study participants showed statistically significant association with the risk of HPV infection which include age 18–25 years; living in an urban community; unmarried status; lower level of educational attainment; current smoker; more than one lifetime sexual partner; and a history of genital warts. A high number of lifetime sexual partners, a history of genital warts, and unmarried status were the risk factors most strongly associated with cervical HPV infection and women who had four or more sexual partners had a 4-fold higher risk of being HPV-positive than women who had only one sexual partner (Roura et al., 2012).

In a Hospital based case control study conducted in Addis Ababa Ethiopia regarding the risk factors for cervical precancerous lesions, age group (40-49), history of sexually transmitted diseases (STD), lifetime sexual partners of the women and the husband were identified as significantly associated risk factors (Teame *et al.*, 2018). In a crossectional study carried out in Paraguay it was found that the risk of hrHPV infection decreased with the age of the woman and increased with the number of lifetime sexual partners. The risk of hrHPV infection increased with the number of previous female partners of their current male partner. Whereas, education level, age at first intercourse, having had different sexual partners during the last year, number of pregnancies, cigarette use, condom use and hormonal contraceptive use were not significantly associated with hrHPV infection (Kasamatsu *et al.*, 2018).

A case control study conducted in Shirdi Sai Baba Cancer Hospital and Research Centre, Manipal, Udupi District, identified marital status (married), history of alcohol use, age at menarche of 13-14, age at first coitus (<18 years), history of abortion and parity of >3 to be the risk factors for cervical cancer (Sharma and Pattanshetty, 2018).

**Prevention and control efforts and challenges**

The incidence and mortality associated with cervical cancer has been considerably declined in resource rich countries during the last decades. This is mainly due to the execution of screening services for the detection of precancerous lesions and HPV and the introduction of better therapeutic options (Denny *et al.*, 2017). Cervical cytology (Papanicolaou) played a significant role in detecting cervical cancer lesions mainly in high income countries though it has been less effective in low and middle income countries (Sankaranarayanan, 2014).

In low and middle income countries, where majority of deaths occur, cervical cancer remains a major public health problem due to limited access to screening and treatment services (Bray *et al.*, 2018). With the advance of technology, new screening technologies like visual inspection with acetic acid (VIA) and HPV-based molecular detection have been introduced by the global oncology community to improve cervical cancer detection (Sahasrabuddhe *et al.*, 2012). In 2013, WHO also suggested HPV-based screening programs as first-line approach, after then many LMICs endorsed HPV-based screening techniques (WHO, 2013).

In African context, though there is policy support in different countries, there have been many challenges facing the prevention and control efforts of cervical cancer which include poor government’s financial commitment, poorly implemented guidelines, low program coverage and low services quality (Tsu *et al.*, 2018). Many countries in sub-Saharan Africa spend less than US$100 per head on all health costs which is very far lower compared to high income countries. Treatment of high number of patients with cancer will be impossible for most health-care systems in sub-Saharan Africa as it generally needs a considerable investment in infrastructure, which is not a reality in many sub-Saharan Africa countries (Abdel-Wahab *et al.*, 2013).

Competing health needs particularly for tuberculosis, malaria, maternal mortality, and HIV epidemic are continuing challenges for cancer control in sub-Saharan Africa. Another important challenge in Africa, identified by WHO, is the scarcity of health care human power which is more prominent in sub-Saharan Africa. In addition, no reliable data exist regarding the number and type of health care providers in sub-Saharan Africa. Lack of adequately trained health-care providers and the brain drain of significant numbers of trained professionals out of Africa is a major problem for cancer control in sub-Saharan Africa (Morhason-Bello *et al.*, 2013).

Though more and better population based cancer registries are the priority for cancer control, in Africa only about 1% of the populations is covered (Sankaranarayanan et al., 2010) . Access to prevention, early diagnosis, treatment, and palliative care for cancer-related disease is insufficient and the availability of cancer treatment is especially poor in Africa. Only 45.3% of African countries reported availability of radiation centers in 2010, even though the number of centers is inadequate in those countries (Abdel-Wahab et al., 2013). Despite the importance of early detection to reduce cancer morbidity and mortality, most people diagnosed with cancer in Africa have advanced disease, which is only suitable for palliative care (Adesina et al., 2013).

Sambo and colleagues in their study also identified eight key cancer prevention and control challenges that are responsible for the cancer burden in sub-Saharan Africa. These include absence of cancer prevention and control policy, strategies, and programs; insufficient cancer data; heavy economic and psychosocial burden of cancer; inadequate or no information about cancer for sustainable research; high cost of immunization against human papillomavirus; unavailability of secondary prevention for cancer; unaffordability of treatment resources and neglect of palliative care; absence of collaboration in stakeholders and donors to combat cancer (Sambo *et al.*, 2012).

One of the main reasons of high cancer mortality in sub Saharan Africa is poor public knowledge and awareness about cancer. Literatures about cancer show that awareness and knowledge in sub-Saharan Africa are at lower levels. For example in a study conducted in Nigeria in 2011 among women attending gynecology clinics, 12.6% were aware of cervical cancer screening and only 8% were knowledgeable about cervical cancer prevention (Mbamara *et al.*, 2011). In another study conducted in South Africa in 2010 among female students, 33·0% were aware of cervical cancer and 31·0% had heard of Pap smear test (Hoque, 2010). In a study conducted in Tanzania among nurses, low level of awareness has demonstrated which could negatively affects accurate diagnosis at the primary care level and causes delays in referrals to specialists, and late diagnosis (Urasa and Darj, 2011).

Cancer advocacy is also an important area of weakness in sub-Saharan Africa (CISN, 2014). In 2011, an analysis of cancer advocacy was undertaken in preparation for the first African Organization for Research and Training in Cancer (AORTIC) and the weaknesses in cancer advocacy in Africa have been identified. Some of the identified internal harmful conditions to the establishment of cancer advocacy in Africa were: competing health priorities; financial barriers; insufficient health-care resources; low awareness and knowledge of cancer advocacy; absence of cancer registry; absence of national cancer plans; lack of collaboration; and poor health systems (Morhason-bello *et al.*, 2013).

The inadequate infrastructure of the health systems in sub-Saharan Africa is a basic reason for poor outcomes, and this contributed undesirably to outcomes of care in cancer conditions (Denny and Anorlu, 2012). The health systems of several African countries are weak due to poor organizational structure manifested by uncoordinated activities at all levels of care (Linden *et al.*, 2012). The most common challenge identified for cancer-control in Africa is the lack of essential health system capacity and infrastructure needed to prevent, detect and treat cancer which generally contributed to poor outcomes in cancer control (NCCPs) (Morhason-Bello *et al.*, 2013). This is also evident in sub Saharan Africa (Petereit and Coleman, 2015).

There are locally operating contextual factors which made cancer control challenging. These include poverty and the financial barriers to treatment, religious and cultural beliefs, stigma around cancer and other medical morbidities. Ultimately, success of any cancer treatment program must take into account the burden of treatment for the patient and her family (Randall and Ghebre, 2016).

Poor mobilization of resources and partners has challenged the control of cancer in Africa. In 2001, according to Abuja declaration, African countries committed to spend at least 15% of the government budget on public health, but only four countries in the region met this target by 2014 (Bhakta *et al.*, 2016). Problem in the organization of national cancer programs within the ministries of Health in African countries is an additional barrier. In most African countries, the structure is inconsistent, creating an obstacle to mobilize resources and partners for NCCP implementation. National cancer control programs are part of the non-communicable disease (NCD) program which is usually coordinated by cancer-control or an NCD program officer with no dedicated cancer-control budget line (Duncan *et al.*, 2019).

There is an increasing movement in medical schools and universities in Africa to improve specialized oncology-related health human power (Rosen *et al.*, 2017). In the meantime, there are promising knowledge-transfer opportunities through technology and partnership which complement the training efforts. In this regard, the development of project Extension for Community Healthcare Outcomes (ECHO) tele-mentoring model by the University of New Mexico which utilizes low-cost technology to promote knowledge sharing and network building through a virtual platform is mentionable (Ryan *et al.*, 2014).

**Current situation of the disease in Ethiopia**

According to world’s summary report of HPV and related diseases in 2019, almost 6,300 new cervical cancer cases and about 4,884 deaths due to cervical cancer occurred among women each year in Ethiopia. This makes cervical cancer the second-most common, and the second-most deadly cancer in the country among Ethiopian women (Bruni *et al.*, 2019).

In Ethiopia, according to the study conducted to assess the pattern of cancer from 1998 to 2010 in Tikur Anbesa specialized hospital (TASH), gynecology malignancy involving cervix, ovary, endometrium and vulva, is found to be the leading malignancies and accounted for 36.6% (Wondemagegnhu, 2015). In the country, the estimated number of new cases of cervical cancer was 6047 with age specific incidence rate of 22% in 2015. Cervical cancer accounts for about 20% of all identified female cancer cases in 2015 (Tessema *et al.*, 2018). In a trend analysis of the TASH cancer registry data, 5293 cervical cancer cases were diagnosed between 1997 and 2012. Cervical cancer cases accounted for 31.8% of all new cases of cancer and showed numeric increment during this period (Abate, 2015).

In Ethiopia the Federal Ministry of Health (FMOH) in collaboration with Pathfinder piloted VIA screening services combined with access to cryotherapy. The service was introduced in 2009 as a single-visit approach to cervical cancer prevention integrated into a comprehensive care package for people living with HIV at 14 Hospitals. Later, based on the findings, the Federal Ministry of Health has scaled up the service further into public healthcare facilities. Accordingly, the Ministry has developed comprehensive cervical cancer prevention and control guideline along with preparation of VIA and cryotherapy training materials (FDRE MOH, 2015).

The national guideline was developed to standardize the implementation of the prevention and control efforts of cervical cancer countrywide. Accordingly, due to the difficulty of sustaining a high quality cervical cytology-based screening program, and the proven effectiveness of the VIA approach, the ministry has considered VIA combined with cryotherapy to be the best approach for cervical cancer prevention and control in the country. The guideline mainly focuses on secondary prevention efforts of the cancer (FDRE M OH, 2015).

Before the implementation of this guideline, nearly 1% of age-eligible women ever received screening (Gakidou, Nordhagen and Obermeyer, 2008). But more recent studies reported an uptake of 9.9% –15.5% in selected populations in Southern and Southwest Ethiopia (Yitagesu, Samuel and Tariku, 2017, Dulla, Daka and Wakgari, 2017, Nigussie, Admassu and Nigussie, 2019). The progress shows a promising trend, though it is far away from 80% target coverage nationally set for the 30-49 years target population by 2020 (FMoH, 2015).

According to the SNNPRG regional projection, currently there are nearly 1,620,774 women aged 30-49 years who are eligible for cervical cancer screening of which cervical screening plan of the region constitutes 206,238 for 2020/21. This represents 12.7% of the eligible women in the region. The 2020/21 nine months’ performance report shows that the screening performance was only 7% (14,108) of the regional plan (206,238) i.e. on average only one woman from twenty women received screening service. The performance varies from zone to zone and Kembata Tembaro zone has been labeled ammong the low performing zones of the region with regard to cervical screening uptake. The zone contributes only 1.2% of the regional performance in the year which generally seeks close attention. On the other hand, the prevalence of positivity for precervical cancer in the reporting period was 4.7% for the zone which is relatively large number capable of calling for public health intervention. This scenario drew our attention to select this area as our study site to understand the contexts, determinants and effect of couple education on the uptake of cervical cancer screening services among eligible women.

# LITERATURE REVIEW

**Effect of health education on knowledge, attitude and uptake of screening**

In the community based clinical trial conducted in Barcelona, three intervention groups (13,886 women) and one control group (2079 women) have participated with the first intervention group receiving a personalized invitation letter, the second intervention group a personalized invitation letter plus informative leaflet, the third intervention group a personalized invitation letter plus the informative leaflet and a personalized phone call three days before the appointment. The control group consisted women whose screening was based on spontaneous demand during the study period. Importantly all the intervention strategies significantly increased women’s participation to screening services compared to the control group. After the intervention screening coverage reached 84.1%, while 64.8% in the control group. The final impact of the study resulted in an increase of 20% from the rescue components in the three IGs and of 9% in the control group (Acera *et al.*, 2017).

A quasi experimental before and after study was conducted among 350 women in each arm in rural Nigeria employing a didactic lectures and video assisted health education intervention followed by a hand bill provided on cervical cancer and screening. In the post intervention, the awareness of cervical cancer and screening and the knowledge and perception was relatively better among intervention group compared to control group and the difference was statistically significant. There was a 4% increase in the uptake of cervical screening which is statistically significant among the intervention group whereas, the change among control group remained nearly the same (Abiodun *et al.*, 2014).

A before and after study was carried out in urban setting of South-East Nigeria among 300 women using trained peer health educators in the delivery of health education intervention on cervical cancer and its prevention. The proportion of participants who screened for cervical cancer increased from 10.5% before the intervention to 17.3% after the intervention which showed statistically significant increase in the rate of screening for cervical cancer (Mbachu, Dim and Ezeoke, 2017). Though the results are significant the study lacks the comparison group to ascribe the results solely to the intervention activities. The study setting is also confined to churches in which findings may however not be inferred to women outside because the characteristics of women in this study may differ from those of other religious groups.

A non-equivalent control group design was used in Ghana to determine the impact of health education intervention on knowledge and perception of cervical cancer and screening among women aged 11 to 70 years (396 in the intervention group and 386 in the control group). A comparison of the mean differences between the pre-post-test of the intervention and control groups suggests a higher mean for knowledge of cervical cancer in the intervention group compared to the control group. The mean difference was statistically significant for knowledge of cervical cancer. Again, the intervention group showed significantly higher mean for knowledge of cervical cancer screening compared to the control group (Ebu *et al.*, 2019).

In the quasi-experimental study conducted in Niger state Nigeria among 93 market women in each arm revealed that the level of awareness about cervical cancer after health education intervention was significantly different when compared to control group. But, after the health education intervention there was no statistically significant difference in the rates of the uptake of the Pap smear test for cervical cancer screening between the groups (Gana *et al.*, 2016).

In a randomized trial conducted to evaluate the effect of a brief health talk on cervical cancer knowledge, attitudes and screening rates in rural Kenya among 419 women attending government clinics for health care services. In the study, a mean knowledge scores increased significantly after the educational intervention. Over half of all participants in both the intervention and the control arms got screened during the study period, with no significant difference in screening rates between the groups. Awareness scores increased significantly more in the intervention compared to control arm at three months follow-up (Rosser, Njoroge and Huchko, 2015).

Even though the level of awareness among the study participants has changed, the study did not explicitly frame the sample size & sampling procedures how the participants were included in the study. This could hinder the validity of the findings to generalize to the target women. Also, behavioral change regarding screening practices would be unexpected in this very brief time period, 30 minutes, because the duration employed for health talks would be too short to bring the desired behavioral changes for screening among the participants.

In the quasi experimental study conducted to determine the effect of health education on the knowledge, attitude and uptake of pap smear among 200 female teachers in Nigeria, the proportion of the respondents with adequate knowledge (mean knowledge score) of both cancer of the cervix and pap smear differed significantly between the intervention and control groups after the intervention. But the rate of uptake was not statistically different between the two arms (Adamu, Abiola and Ibrahim, 2012). The study participants in this study had not been followed between the last date of intervention and the date of data collection three months later post intervention. The study participants have only been provided coupon for the Pap smear services on the last date of intervention and left free of follow up at which time data was collected three months after the intervention.

A quasi experimental study was conducted to examine changes in knowledge and behavior after a community based cervical cancer education program in Honduras among 600 women. In this study the effectiveness of the radio broadcasts was assessed and women surveyed after the radio broadcasts knew more than women surveyed before the radio broadcasts on all questions related to cervical cancer and Pap smears with statistically significant results (Perkins *et al.*, 2007). The measurement was taken in two different time frames between two different groups of women which ignored the possible effect of time on the outcomes of the study.

In the effectiveness study of theory based education intervention aimed at improving awareness and screening for cervical cancer in Jamaica, which is crossectional pre-post study, among 225 women, the intervention significantly increased knowledge of and intention to screen for cervical cancer, but did not bring significant changes in screening rates for cervical cancer (Interis *et al.*, 2015). The effect of the intervention was not controlled in this study in that it only measured and compared the changes over knowledge, attitude and screening rates before and after the intervention. This does not guarantee the causal inference between the intervention & the outcome.

A crossectional descriptive interventional study was conducted among 380 secondary school teachers in Nigeria in order to evaluate the impact of health education on knowledge, attitude and practice of cervical cancer screening. Accordingly, knowledge on cervical cancer and cervical cancer screening significantly improved after intervention. Also, health education as the intervention in this study has brought significant impact on the practice of cervical cancer screening among the study group (Chinwe R, 2015). This is a pre-post type of study where measurements are undertaken before and after health education. Though the results are statistically significant it is difficult to ascribe the changes to the intervention in the absence of the control group.

In a randomized controlled trial conducted to evaluate the effectiveness of interventions in increasing screening participation among 356 women aged 30-65 years in Turkey, three modalities of interventions namely brochure and education, brochure only and invitation only were employed. Accordingly, the cervical cancer screening (CCS) rates were higher in the brochure + education group and the difference between the groups was statistically highly significant. A statistically significant difference was identified between the intervention groups in terms of post-intervention knowledge scores. With regard to intervention related differences, the post-intervention knowledge scores in the brochure + education and brochure only groups were significantly higher compared to the invitation-only group. When the change between the pre-intervention and post intervention knowledge was assessed by group, the difference in the brochure + education group was significantly higher than in both the brochure-only group and the invitation-only group. Furthermore, the knowledge increase in the brochure-only group was significantly higher than in the invitation-only group (Kurt and Akyuz, 2019).

A hospital based interventional study was conducted in Ibadan, Nigeria to evaluate its effect on women’s knowledge and uptake of cervical cancer screening among 904 women. An educational intervention was provided in the form of focused health information on cervical cancer and screening given to women attending antenatal clinics. Both the awareness and knowledge levels were significantly increased in the intervention group after the intervention compared to control group. The actual practice of utilizing screening services did not show significant improvement after the intervention between groups (Ndikom *et al.*, 2017). In this study participants were not properly invited in a clear way to have cervical cancer screening. There is also methodological gap identified in terms of explicitly explaining and emphasizing about the uptake of cervical cancer screening.

A pilot randomized controlled trial was conducted among 42 south Asian women to evaluate effects of a community health worker led multimedia intervention on the uptake of cervical cancer screening. The pilot study revealed that, no significant difference was noted in screening uptake and screening intention between the arms (Wong *et al.*, 2019).

In a study conducted among rural chines women to assess the effect of a group education intervention on the knowledge about human papilloma virus and cervical cancer, the intervention significantly increases the awareness regarding HPV and cervical cancer (Li *et al.*, 2015). In this particular study the sample size issue was not addressed to enable readers evaluate its adequacy for the objectives and weigh the external validity of the findings. It is the same day research, as all the procedures i.e. pre-intervention assessment, intervention administration and post intervention assessment were completed on the same day which could have hampered the quality of the overall research process. This quality of the study would be questionable.

A randomized controlled trial was carried out in Cameron among 302 women between age 25 to 65 to evaluate impact of an educational intervention on knowledge and acceptability of HPV self-sampling. Video based group health education was provided to the intervention group in addition to basic information provided to both groups. Consequently, participants who received the educational intervention had a significantly higher knowledge about HPV and cervical cancer than the control group (Sossauer *et al.*, 2014). In this study sampling techniques employed to recruit study participants were non probabilistic in that only those women who are interested and avail themselves on the days of sampling were included in the study. This would lead to problem in the external validity of the findings.

A cluster randomized trial was conducted to evaluate the effect of a call recall program in enhancing a Pap smear screening practice among 398 participants in 40 cluster schools using a trans-theoretical model. The trial included a personal invitation letter with an information pamphlet of cervical cancer screening, followed by a telephone reminder with counseling after four weeks. Significantly higher proportion of participants in the intervention arm were found to be in action stage than the control group (Abdullah and Su, 2013).

In a quasi-experimental study evaluating the effects of group counseling based on the Health Belief Model (HBM) on cervical cancer screening practices among rural Iranian women, there had been a significant increase in the proportion of women underwent screening practices in the intervention group compared to control group (Parsa *et al.*, 2017).

In a Turkish randomized controlled trial where the effect of Health Education about cervical cancer and papanicolaou testing on the behavior, knowledge, and beliefs was evaluated among 148 women aged 20 years and above, the difference in test scores, which represented knowledge about cervical cancer testing, was statistically significant between the control group and the study group (Bebis *et al.*, 2012). The study was limited to a certain group of women living in the apartments which might not represent the general women living in other locations. In addition, appropriate sampling techniques were not employed for participants’ selection in a way that ensures probabilistic approaches. Therefore, the findings could not be generalized.

The effectiveness of behavior change communication (BCC) messages delivered via short message service (SMS) on the uptake of CCS in the Kilimanjaro and Arusha regions was assessed by randomized controlled trial. The study revealed that participants in the intervention groups were more likely to attend CCS than participants in the control group and the effect of both interventions were significant in both urban and rural areas in the uptake of the services (Erwin *et al.*, 2019). This study involved only women who had access to mobile phones which excluded those women without access to mobile phones who otherwise can participate in the study. Excluding those women on the bases of mobile phones’ ownership might create ethical issues and the findings could not be utilized among such women groups.

According to a quasi-randomized controlled trial conducted among 240 women to determine the effects of an educational intervention based on the protection motivation theory and implementation intentions on first and second Pap test practice in Iran, significantly higher percent of women in the intervention group had obtained first and second Pap test compared to the controls (Dehdari *et al.*, 2014). A convenience sample of women was taken to randomly assign to the intervention and control group which might hamper the findings of the study to be inferred to the target women.

A three stages effectiveness quasi-experimental study was conducted to increase Pap smear test screening among 2500 women in Ankara, Turkey. Cervical cancer, Pap smear test, and total knowledge scores of women significantly improved after receiving the study’s educational brochure. The intervention also effectively increased participation in cervical cancer screening with free Pap smear testing (Guvenc, Akyuz and Yenen, 2013). In this study women, with available names and telephone numbers in a certain locality were targeted for the study which systematically excluded those women whose name was not in the list. This could have created characteristically non representative sample for the study.

Mobile phone text messaging intervention for cervical cancer screening was evaluated for its effectiveness on knowledge among Korean American women aged 21-29 using quasi-experimental research design. As a result significant improvements were observed for general knowledge about cervical cancer and the Pap test (Lee *et al.*, 2014).

In a prospective randomized controlled study conducted in Japan to see the effect of providing risk information on undergoing cervical cancer screening among women aged 20-39, significant proportion of women in the intervention group participated in the screening when compared to control group (Fujiwara *et al.*, 2015).

A quasi experimental study was conducted in Losangeles among 504 Thai women aged 18 and above to compare the effectiveness of video based education with brochure on knowledge. After the study it was concluded that no uniform differences in knowledge were found between the intervention group (video) and the control group (brochure). Both educational modalities demonstrated in selected increases in knowledge and attitudes (Love and Tanjasiri, 2012). Though the study employed strong study design, on the other hand it demonstrated non probabilistic techniques in recruiting the study participants which would introduce nuisance in the external validity of the results and consequently leads to limited utilization.

A video and brochure based educational intervention study was conducted among 41 pairs of mother/daughter using a quasi-experimental design to see its effectiveness on the knowledge regarding cervical cancer. The study revealed that there was a significant knowledge-level increase in response to the information presented during the intervention (Obulaney, Gilliland and Cassells, 2016). The study involved quasi experimental with pre-post assessments without employing control group. This raises doubts in the validity of the results whether the results are purely ascribed to the intervention alone.

In Ohio, a randomized controlled trial was carried out to evaluate the efficacy of lay health advisors (LHA) in increasing risk appropriate Pap test screening. Participants in the intervention arm received 2 in-person visits, 2 telephone calls, and 4 postcards from an LHA for over 10 months period. According to medical record review, a greater proportion of women in the intervention group had a Pap test at the study completion than women in the control group, but the findings are not significant (Paskett *et al.*, 2011).

The effects of two different invitation models on participation in cervical cancer screening were investigated in a randomized population based cohort study in Germany among 7,758 eligible women aged 30–65 years. Participants were randomly selected via population registries and randomized into intervention arm A (invitation letter) and B (invitation letter and information brochure) or control arm C (no invitation). Accordingly, women in the intervention group showed significantly highier proportion, 2.62-fold, of participating in CCS after being invited compared to women in the control group with no invitation (Radde *et al.*, 2016).

A three arms randomized controlled trial was carried out to evaluate the effectiveness of video only intervention with video plus home based education session among Latinas aged 21 to 64 years who were non adherent to Pap test screening schedule. The study demonstrated that significantly more women in the high intensity arm /video plus education/ received a Pap test in comparison with the low-intensity arm and the usual-care arm (Thompson *et al.*, 2017).

**Summary of the evidence**

Literature review shows that different studies employed different health education intervention modalities to examine their effectiveness in different settings and target groups. The studies used different designs which mainly included quasi-experimental and cluster RCT. They also used different sample size requirements though some studies employed no comparison group and non-probabilistic sampling procedures for participant recruitment. In these studies, the researchers followed the study participants for varied lengths of the intervention period. The studies generated different outcomes with respect to their intended outcomes of interest. Some studies showed that the intended interventions worked well and resulted in significant effects on the outcome between the arms whereas other studies showed that the health intervention modalities did not work well to affect the outcome positively. The available published evidence shows that studies have not been conducted yet in Ethiopian context to determine the effectiveness of education intervention.

This shows that, health education interventions have not been tested in Ethiopian context whether it works well or not in increasing cervical cancer screening. Therefore, studies involving strong designs are required to bridge the observed information gaps in this area. Also, none of the published studies elsewhere have tested the effect of couple education & counseling in promoting cervical cancer screening uptake in their educational interventions. Moreover, the existing evidence demonstrates only the effects of interventions targeting at individual or group level. None of the reviewed trials showed the effect of couple based education and conseling on the uptake of cervical cancer screening which remained a gap area in this regard. Therefore, employing couple based education intervention can be considered as an inovative approach in our study. The researchers also belive that testing the effectiveness of couple education and counseling in the women’s uptake of screning services in such male dominant cultures might bring an important input to design relevant public health intervention strategies.

# CONCEPTUAL FRAMEWORK

Knowledge & Beliefs about Cervical Cancer & its Screening

Couple Education

&

Counselling

Cervical

Cancer

Screening

Uptake

**Socio-demographic Factors**

Age, Education, Occupation, Marital Status, Age at marriage, Family income, Family size, Ethnicity, Parity, Religion, WDA member

**Exposure to Cervical Cancer & Screening Information**

Awareness, Exposure to health information, Get advice from HPs, Know someone screened, Know someone with CCa, Know screening location

**Health Service Related factors**

Health insurance, Distance from HF, Visit to HF within 12 months, Use of maternal services, Visits by HEWs

Figure 1 Conceptual frame work of the study developed from the literature review

Women’s knowledge about cervical cancer influences their belief system and consequently leads to positive behavior change and uptake of screening services.

# SIGNIFICANCE OF THE STUDY

In low- and middle-income countries, including Ethiopia, cervical cancer is the commonest cancer and the leading cause of cancer deaths next to breast cancer among women. Also, the majority of cases (over 80%) in sub-Saharan Africa including Ethiopia are detected at a late stage of disease where treatment modalities may be limited, expensive or inaccessible, for many women in such countries. This is predominantly due to lack of information & poor preventive measures like screening and treatment at early stages. Effective screening programs can lead to a significant reduction in the morbidity and mortality associated with advanced disease through early detection and treatment as seen in developed world. Despite the importace and availability of screening services, only few of eligible women underwent screening for cervical cancer in low-resource countries, including Ethiopia. Also researches have not been conducted in the effectiveness of education interventions related to cervical cancer screening uptake among eligible women. Our research, therefore, aims to test the effectiveness of education intervention in increasing screening services uptake among age eligible women.

We also believe that contextual factors operate & could influence the effect of health education intervention on cervical cancer screening uptake. As a result basic studies need to be conducted in Ethiopian setting in this regard to evaluate the effect of interventions on screening uptake rather than solely relying on findings of systematic review of international evidence. Therefore, this research produces an important insight in this regard and contributes to the scientific knowledge as far as Ethiopian context is concerned. The study also contributes to bridge an observed information gap in Ethiopia during literature review as far as effectiveness studies are concerned and contributes to the national cervical cancer prevention and control efforts. The findings of our research, when implemented, benefit risky women by averting the burden of the disease through early detection and treatment modalities. Identification of determinant factors affecting the uptake of cervical screening will drive the linkage of modifiable factors with appropriate intervention modalities.

In general, the findings of this research enable local and national health authorities to evaluate policy directions and plan effective health education interventions through escaling up the intervention in the health sector particularly at the health post level to increase cervical screening uptake and reduce the disease burden through early detection and treatment.

# RESEARCH QUESTIONS

**Research Questions**

1. Does couple education and counseling about cervical cancer and its screening increase women’s level of knowledge regarding cervical cancer and its screening?
2. Does couple education and counseling about cervical cancer and its screening improve women’s attitude regarding cervical cancer and its screening?
3. Does couple education and counseling about cervical cancer & its screening and invitation increase cervical cancer screening uptake among women?

# AIMS OF THE THESIS /GENERAL OBJECTIVES

**General Objective**

The general objective of this study is to determine the effect of couple education on the knowledge, attitude and uptake of cervical cancer screening services among women of child bearing age in Southern Ethiopia

**Specific Objectives**

1. To determine the effect of couple education and counseling about cervical cancer and its screening on the level of women’s knowledge regarding cervical cancer and screening practices
2. To determine the effect of couple education and counseling about cervical cancer and its screening on the women’s attitude regarding cervical cancer and screening practices
3. To evaluate the effect of couple education and counseling about cervical cancer and its screening and invitation on cervical cancer screening uptake among eligible women of child bearing age

# RESEARCH METHODS AND MATERIALS

**Study setting /area/context**

The geographic location for our study is Kembata Tembaro and Hadiya zones which are located in the Southern Nations Nationalities and Peoples' Regional State. The total number of age eligible women (30-49 years) for cervical cancer screening in the study zones constitutes 19.2% of women of reproductive age group residing in the zones. Kembata Tembaro zone comprises of 8 districts and 3 city administrations with the total number of 150 clusters or kebeles, the smallest administrative units, of which 9 are located in the city administrations. The zone has one general hospital, four primary hospitals, 33 health centers and 138 health posts. Hadiya zone is administratively organized in to 13 districts and 4 city administrations having a total number of 329 clusters. The zone has one comprehensive specialized hospital, three primary hospitals, 61 health centers and 317 health posts.

The health care of women of child bearing age in general and maternal & child health care services in particular are of the main strategic pillars of the health service delivery efforts in the zones. Cervical cancer prevention and control activities are being integrated in to the routine health care delivery as an important health service package for the women’s health. Currently, cervical cancer screening services are being implemented at selected hospitals and health centers of the zones where our research activities will base these facilities.

The study will be conducted in 2021.


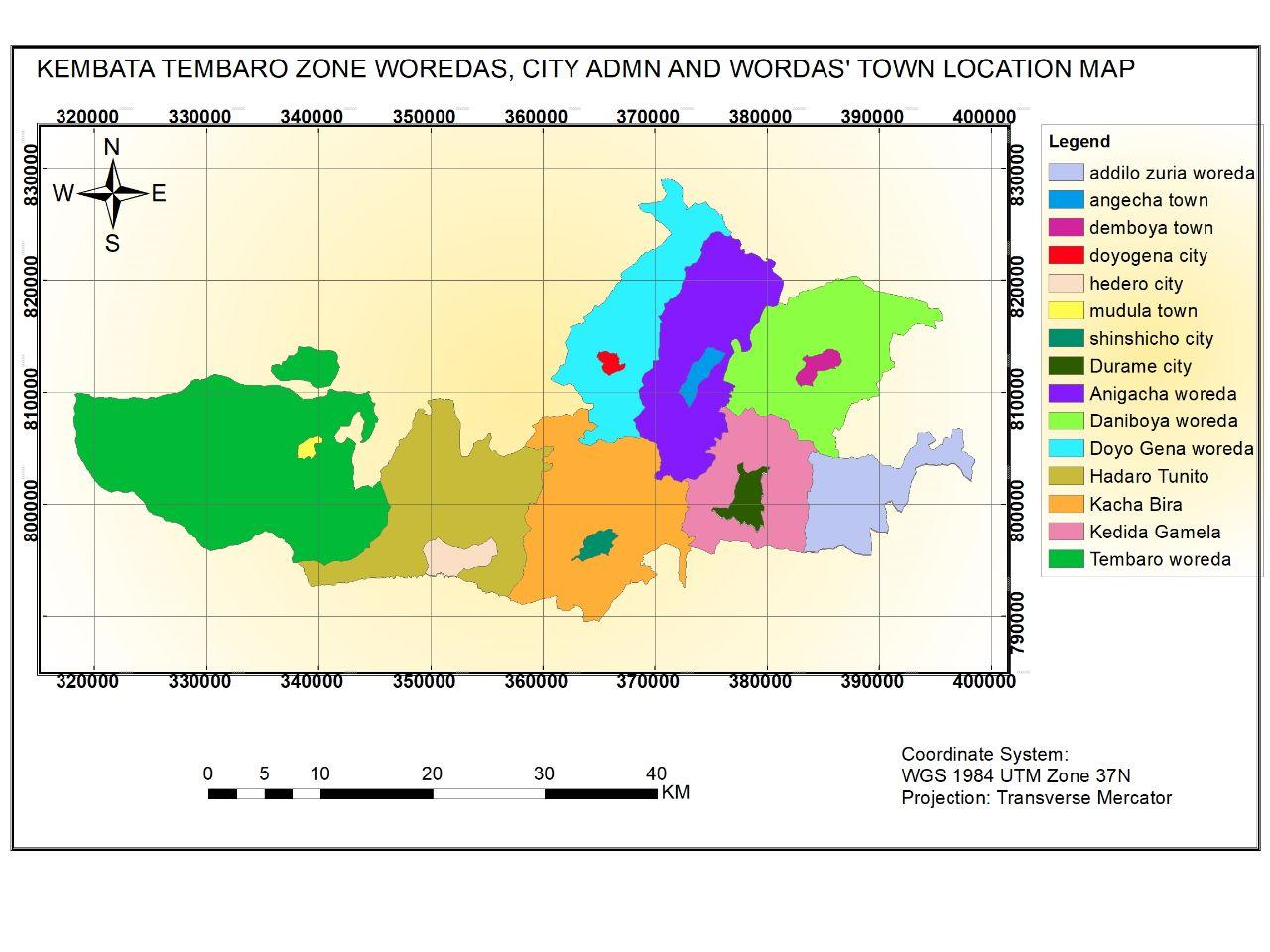


Figure 2 Map of study zone; Source Zonal Health Department

**Study Design**

We will employ cluster randomized controlled study design.

**Source population**

The source population for our study will be entirely age eligible women (30-49 years) of child bearing age living in the zones who had history of sexual experience.

**Study population with eligibility criteria**

The study population will be women of child bearing age who are eligible for cervical cancer screening according to the national guideline. Accordingly women aged 30-49 years are targets for cervical cancer screening program and our research will be carried out within this functional framework. Women should also satisfy the requirements of legal residency within their respective living quarters that is at least six months of residence. Our study population needs to satisfy additional eligibility criteria such as having been engaged in sexual relations, have not had received the services within the last 5 years period and have not been diagnosed for cervical cancer and can provide informed consent.

Women with any gynecologic cancer, who had hysterectomy, received screening test before, who are pregnant and in the period of three months postpartum will be excluded from the study. Study participants represent women population who will systematically be selected based on the appropriate probabilistic sampling techniques. Eligible women will be identified by censusing.

**Randomization**

The clusters or kebeles are the units of randomization in our study. Initially, sixteen non-adjacent clusters, eight from each district, were identified from the two study districts. We stratified the clusters based on the study districts and created separate list of eight clusters alphabetically for each district. The stratification was done to evenly distribute any known and unknown district level confounders across the study arms. Each cluster was assigned a unique cluster code. Then, the statistician assigned the eight clusters in to two blocks of size 4 according to the order they appeared alphabetically. The statistician randomly selected the randomization sequence of clusters for each block using the sealed lots of the six possible permutations within the block.

The clusters in each block were randomly assigned to intervention and control arms according to the randomization sequence evident by the selected permutation within in the block for the stratum. We repeated the same process for blocks of clusters in the other stratum. Consequently, four clusters were obtained from each district which formed eight clusters to the intervention arm and eight clusters to the control arm maintaining 1:1 random allocation ratio. The statistician was made unaware of the actual study arms to mask the knowledge about which group will receive the intervention and which group will receive the usual care. This can be achieved by representing the study arms and clusters by confidential codes.

**Contamination reduction strategy**

When women from intervention kebeles join those women in the control kebeles, the control kebeles will be contaminated with the intervention messages. In the rural areas this may happen when different social groups come in to contact due to different social occasions including funeral, wedding, marketing etc. Informal discussions of ideas regarding the intervention activities may arise on such occasions and individuals from control kebeles may gain some health messages which may affect the intervention effect negatively. To reduce such an undesirable effect of information contamination, a buffer zone will be created to separate clusters of the two arms by making use of kebeles that will not take part in our study. As a result 8 non-adjacent kebeles will be selected from each study district.

**Intervention description**

The proposed intervention modality for our trial will be brochure assisted home based couple education and counseling followed by formal invitation for cervical cancer screening. All age eligible women in the intervention clusters who consented to participate will be exposed to the proposed intervention. The woman and her husband will be educated about cervical cancer and its screening and counseled on the importance of screening for cervical cancer. The husband will also be counseled on the importance of providing support and encouragement to his wife. In addition, the woman will receive formal letter of invitation which reinforces key messages of education intervention and importance of getting screened for cervical cancer.

The educational and counseling material is organized to address susceptibility to cervical cancer, its seriousness, the benefits of screening and barriers to screening with the objective to build cues to action and develop woman’s self-efficacy. The intervention is organized and designed to bridge the knowledge gap and positively influence women’s belief system related to cervical cancer and its screening. This, in effect would bring positive behavior change among women to use cervical cancer screening services.

The educational brochure consists of four sections. Section one provides general information on the definitions, magnitude and incidence of the disease. Section two gives information and knowledge of risk factors, signs and symptoms, complications, and preventive modalities of the disease. Section three offers an explanation of the eligibility criteria, screening schedule, benefits and barriers to screening services to encourage participants to adopt positive behaviors. Section four explains the meanings of screening results, available treatment options, the cost, location of the service and importance of male involvement in woman’s cervical cancer screening uptake.

After the development of the educational material, its face and content validity were checked and assessed by professional experts from behavioural sciences and their comments were incorporated to further increase the effectiveness and efficiency of the material with respect to study objectives.

**Implementation of the intervention**

Each woman will receive a total of three contacts during the intervention period. Accordingly, each eligible woman in the intervention clusters will be physically visited three times at her residential home. During the imitial contact, a 45 minutes leaflet guided education & counseling session will be held with the woman in her residential home to convey information on cervical cancer and its screening and counseling to encourage the uptake of screening. At the end of the session, discussion will be held with the woman to address any questions and concerns. The woman will also be provided with the educational brochure for further reading at least once per week by themselves or to be read by any litrate person within the family or neighbourhood. The date of the first follow up visit will be made consensually with the woman to attend the session together with her husband.

A follow up visit will be made to each woman one month after the initial visit with the objectives to re-emphasize important points of the material and make encouragement for screening. During this visit both the woman and her husband together will receive key information on cervical cancer and the importance of its screening. The husband will also be counseled on the importance and the way how the woman receives his support and encoyuragement to get screened for cervical cancer. Additionally any misconceptions related with the information will be corrected and barriers to screening will be addressed during this visit. Repeated follo up visits will be made in a case of absence of the couples.

Finally, the second follow up visit, which is the last visit of the our intervention modality, will be made one month after the first follow up visit to convey key messages about cervical cancer and its screening and address any unresolved concerns related to cervical cancer and its screening. Also, a formal letter of invitation will be granted during this visit for free screening services available in the nearby health facility. During all the visits elgible women will be asked key questions at the end of education and counseling session to check their comprehension and also encouraged to get screened for cervical cancer.

The intervention period will last for six months in two phases. Phase one involves the first three months of active delivery of the intervention at which time intervention is actively provided to each woman in a monthly bases. The second phase consists of the last three months of passive period. During the last three months women will not receive any intervention scheme but left unvisited with the objective to provide a period to translate their knowledge in to practice. The health extension workers of the respective intervention clusters will be trained on the provision of the intervention to couples at their homes. The control group will receive the usual standard of care. The control group will be exposed to the same intervention after the trial period through the routine services delivery schemes.

**Implementation of screening service**

Those women who visit the health facilities will rceive the screening service according to the national guideline. Women will be provided with the specific information whom they will contact and how they will acces the service delivery point within the health facility. Women who are interested to be screened and come from the study clusters will be linked to the administrative procedures of the health fcaility to receive the screening service. A trained health professional will conduct the screening procedure using visual inspection techniques according to the implementation guideline after completing assessment and counseling. Any woman with positive precancerous screening results will receive appropriate treatment immediately after the results become available on the same day visit. Those women who are positive for cervical cancer will be counseled to get screened after one year but after five years for those who screened negative. All women who rceive the screening will be registered on the format prepared for study purpose.

**Compliance parameter**

All the eligible women in general and study participants in particular in the intervention clusters may not fully comply to the proposed intervention as recommended due to different participant and provider related factors. This might have undesirable consequences on the uptake of the screning services. The impact of variability in the compliance to the intervention will be considered during analysis as a dose response function. Therefore, compliance checklist will be developed and used to record the level of women’s exposure to our proposed intervention to account for during analysis.

Table 1 Summary of the intervention modality

| Content of the intervention | Dosage | Frequency | Duration | Compliance parameter |
| --- | --- | --- | --- | --- |
| Leaflet guided couple education & counseling | 45 minutes | Every month | Two months | No of couples educated & counseled |
| Leaflet guided couple education & counseling and formal invitation | 45 minutes | Once a month | One month | No of couples educated & counseled; and formally invited |

**Mechanism of the Health Belief Model**


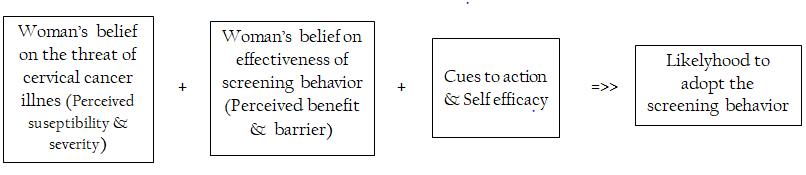


Figure 3 the mechanism how the health belief model works

The model shows that a woman’s belief on a personal threat of cervical cancer together with her belief in the effectiveness of the screening behavior will predict the likelihood she will practice the behavior. The woman’s course of action depends on her perceptions of the benefits and barriers related to screening behavior. When the woman’s awareness, attitude & self efficacy is positively influenced by the health intervention and cues to action are made available, she will likely put the desired behaviour in to practice.

**Project implementation framework**


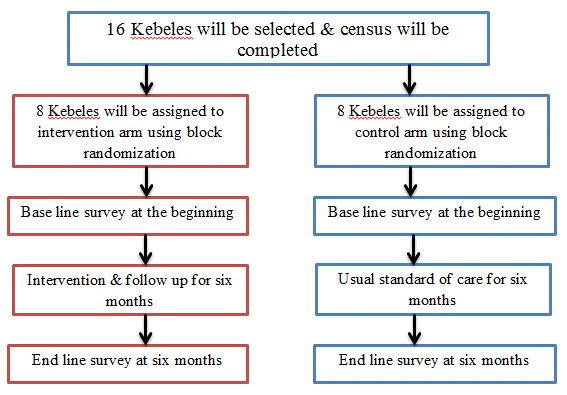


Figure 4 Implementation frame work for the intervention project

**Sample size determination**

The required number of study participants per arm for our cluster randomized control trial was calculated using the following formula (Rutterford, Copas and Eldridge, 2015):

| **n** = (*zα/2+ zβ***) ^2^ [**p1 (1-p1) + p2 (1-p2)**]** x [1+ (m-1) *ρ*]  (p1-p2)^2^ |
| --- |

Where

*zα/2 is the value of z at α =0.05 significance level which is 1.96*

*zβ is the value of z at 1-β power of 80% which is 0.84*

p1 is the proportion of outcome in the control group (15.5%)

p2 is the proportion of outcome in the intervention group (35.5% which is p1 plus effect size)

m is the cluster size

*ρ* is the intra cluster correlation coefficient

1+ (m-1) *ρ* is taken as the design effect which is 2 in our case

Based on published cervical cancer screening rates, we considered cervical cancer screening rate of 15.5% among age eligible women /30-49 years/ conducted in Jimma town (Nigussie, Admassu and Nigussie, 2019) as the proportion of outcome in the control group.

We expect to detect an absolute difference of 20% increase in screening proportions between the groups with the power of 80%, and 5% significance level for one-tailed test. We also considered the design effect of 2 to adjust for the loss of variability due to clusting and 5% compensation for incomplete and non-response rates that would happen during data collection. The difference of 20% is an average that was taken from review of different published studies that showed an increase in cervical cancer screening proportion ranging from 6% to 50% following health education interventions.

Eventhoug we indicated formula for sample size above to show the parameters, we used the G*power software to calculate the required sample size. As a result the number of participants per arm will be 66. Therefore, our total sample size will be 132 study participants. Using the design effect of 2 and non-response rate of 5% will produce the total participants’ size of 288 women age 30-49 years. The required number of clusters we will use for our study will be sixteen.

**Sampling technique/procedure**

Sixteen clusters (kebeles) will be selected from the two study districts where cervical cancer screening service is currently available. Sixteen clusters are taken based on the recommendation that taking fewer subjects from many cluster give better represenatation of the sample than taking many participants from fewer clusters. The clusters will be randomly assigned in to intervention and control groups. Census will be carried out in the selected Kebeles to identify women who are eligible for screening and to create sampling frame. Then, simple random sampling technique will be performed to select study participants in each arm. We will select equal number of participants from each cluster which consequently leads to selection of 18 participants per cluster.

The same sample of participants will be used at the end of the intervention phase, six months later, to measure outcome variables.

**Simple RS**

**Block randomization**

Figure 5 Sampling frame for the intervention study

**Data collection and measurement**

Data will be collected using structured questionnaire designed for the purpose of meeting specific research objectives. The tool has been developed based on the research objectives from relevant literature sources. The data collection instrument will be pretested to check for its clarity, logical sequence, cultural appropriateness etc. and appropriate modification will be made accordingly. For CRT, participants will be interviewed to complete baseline and end line survey about their characteristics, knowledge, attitudes, and cervical cancer screening uptake.

Data will be collected by face to face interviewing technique with each woman at her home. Data collectors will go to each selected woman’s home physically by carrying all the data collection tools and communicate verbally to get the relevant information as per the questionnaire. Allocation of intervention will be masked from data collectors by employing two different groups for intervention administration and data collection. Both the baseline and end line data will be collected by individuals who partcipated in the collection of censusing data. Health extension workers facilitate the census work and data collection process.

**Operational definitions and measurements**

Educational intervention is to mean leaflet assisted home based couple education and counseling followed by official invitation and reminder phone calls to help receive cervical cancer screening

Couple education and counseling in this study referes to the process of communicating relevant information on cervical cancer and screening with couples to ensure male involvment in assisting and encouraging the woman to get screened for cervical cancer.

Precancerous cervical lession is an abnormal cellular change located around the cervix, which has not yet regressed or progressed to invasive cervical cancer

Cervical cancer means malignant, autonomous, and uncontrolled growth of cells of the cervix

Cervical cancer screening is a procedure that is performed to identify the presence or growth of abnormal cell in the cervical tissue.

Cervical cancer screening uptake means having experience of cervical cancer screening before or within the study period reported by the participant during the interview

Visual inspection of the cervix with acetic acid (VIA): Screening method which involves naked-eye inspection of the uterine cervix 1 min after application of a 3–5% solution of acetic acid using a cotton swab or a spray.

Knowledge: Questions will be delivered to study participants in multiple choice options, and they will agreed or disagree the options based on their awareness. Each correct response will be given a score of 1 and a wrong answer given a score of 0. We will compute the mean score to determine the overall knowledge of cervical cancer screening of respondents and will be classified as poor and good based on the mean score.

Attitude: we will assess the attitude using a Likert scale using three options disagree = 1, neutral = 2 and agree = 3. The responses will be summed up and a total score will be obtained. Then we will calculate the mean score. Those who will score the mean and above will be considered as having a positive attitude, whereas those women who will score below the mean will be categorized as having negative attitudes towards cervical cancer & its screening.

Male involvement is the extent to which men are involved and supportive of women's needs, choices, and rights in getting screened for cervical cancer as determined by composite score. The composite score will be calculated from participants’ responses, where 1 represents male involvement and 0 not. The scores will be summed up and those who scored mean value and above are considered as involved and below the mean considered not involved.

**Data processing and management**

Specific code will be assigned for each of the completed questionnaire. Cluster and individual code will be assigned for questionnaire used for CRCT. The data will be entered using Epi info 3.5.3 version. The data will be edited using the entry software. Then the data will be transported to either SPSS or STATA to carry the desired statistical analysis.

**Data analysis**

Base line screening coverage will be measured after randomization and before the initiation of intervention for the last 5 years screening experience of women for both arms. We will also measure factors associated with screening practices, their knowledge and attitude regarding cervical cancer and its screening. The groups will be examined and compared at base line for any statistical differences in terms of base line participants’ characteristics and their screening experiences using Chi-square test for categorical variables and independent sample t-tests for continuous variables. That is, we will compare the primary outcome measure (screening uptake) in the intervention and control groups using x^2^ tests and secondary outcome measures (i.e. knowledge and attitude) using the t-test. A one-sided P value of 0.05 will be used to determine statistical significance. Logistic regression analysis of factors associated with screening practices for cervical cancer will be carried out at baseline.

End line or follow up outcome assessment will be done to measure women’s knowledge, attitude and screening practices at sixth month of the intervention for both the intervention and control groups. Screening proportions after the intervention will be calculated by taking proportions of women screened during the trial period plus within the last 5 years before the intervention. Intention to treat approach will be employed to analyze the data. We will use paired sample t-test to test knowledge about cervical cancer and cervical cancer screening, within the intervention group by comparing the before and after intervention scores. A similar analysis will be done for the control group to compare before and after intervention scores. Then we will employ the independent-sample t-test to determine the effect of the intervention on knowledge and attitude by comparing participants’ scores on knowledge and atitude about cervical cancer and its screening, between the two groups.

Finally, we will use Generalized Estimating Equation (GEE) analysis technique to test the independent effect of the intervention on the primary outcome of the study. The impact of age on intervention will be analyzed across different age groups.

**Quality assurance**

The questionnaire has been translated in to the local language and back translated to English to increase its validity. Two days training will be provided for the data collectors and supervisors before data collection process. The questionnaire will be pretested and checked by taking 5% of the sample size to increase the validity of the measurement and check for their clarity, logical sequence, cultural appropriateness etc. and will be revised accordingly. Questionnaire will be checked for its consistency and completeness by the supervisors on daily basis during the entire data collection period. Incomplete questionnaire will be made complete by making repeated visits to the home of the woman. Qualitative data collectors and supervisors will be oriented particularly on how to interview, take notes and manage audio recording sessions of the data collection.

**Ethical considerations**

Ethical approval for the research will be first obtained from the Jimma University ethical review Board. Written permission will also be obtained from the regional health bureau, Zonal health departments, respective districts and kebele administrations. The objectives of the study will be explained for each participant and informed consent will be obtained to ensure voluntary participation. Participants will also be assured of the confidentiality of the information they will provide.

Participants’ information sheet and consent form will be attached to the questionnaire for appropriate guidance of the data collectors and address ethical issues appropriately. Woman who has any concern about the study subject can be counseled and appropriate information will be provided. Women with any apparent health problem will be counseled and referred to health facility for appropriate care. Participants who will be positive for cervical cancer will be linked to the appropriate care and treatment modalities based on the nature of the cancer identified. Those who are positive for cervical cancer will be appointed after one year for checkup but negative women will be informed about the next follow up visit after 5years.

**Plan for dissemination of the findings**

The findings of these studies will be disseminated through presentations to the scientific community of Jimma University. It will be communicated with stake holders elsewhere including regional & Zonal health authorities during review meetings. The research findings will also be published in national or international reputable and peer reviewed publishing journals.

# A SUMMARY OF THE PROJECT WORK

Table 2 Brief summary of the project work

| Objectives | Study Design | Sample Size | Sampling method | Population | Data Source | Outcome measure | Analytic Model |
| --- | --- | --- | --- | --- | --- | --- | --- |
| Effect of couple Education on knowledge, attitude & CC screening uptake | Cluster RCT | 288 | Simple random sampling | WCBA | Baseline & end line Survey | Effectiveness of couple education (change in KAP) | Generalized Estimating Equation (GEE) |

# BUDGET BREAK DOWN

Table 3 Detailed budget breakdown

| **Project one: Interventional study** | | | | | | | | | | | | | |  |
| --- | --- | --- | --- | --- | --- | --- | --- | --- | --- | --- | --- | --- | --- | --- |
|  | 1. Survey cost (two surveys, baseline and end line, will be conducted) | | | | | | | | | | |  |  |  |
| SN | Budget Category | | Unit Cost | | Multiplying factor | | | | | Total Cost | | Remark |  |  |
| 1 | Training & pretest cost | |  | | Quantity | | No of days | | |  | |  |  |  |
|  | Per-diem | | 325.00 | | 10+2 pers | | 3 days | | | 11700.00 | | 2supervisors |  |  |
|  | Coffee/tea | | 3ETB | | 12persons | | 3 days | | | 108.00 | |  |  |  |
|  | Soft drinks | | 10ETB | | 12persons | | 3 days | | | 360.00 | |  |  |  |
|  | Cookies | | 120/kg | | 12persons | | 3 days | | | 360.00 | | 3kg |  |  |
| 2 | Stationary cost | |  | | Quantity | |  | | |  | |  |  |  |
|  | Pen | | 10ETB | | 12 items | | -- | | | 120.00 | |  |  |  |
|  | Note book | | 25ETB | | 12 items | | -- | | | 300.00 | |  |  |  |
|  | Pencil | | 5ETB | | 12 items | | -- | | | 60.00 | |  |  |  |
|  | Eraser | | 5ETB | | 12 items | | -- | | | 60.00 | |  |  |  |
|  | Case book | | 45ETB | | 12 items | | -- | | | 540.00 | |  |  |  |
|  | Sharpener | | 10ETB | | 12 items | | -- | | | 120.00 | |  |  |  |
|  | Flip chart | | 150ETB | | 1 pad | | -- | | | 150.00 | |  |  |  |
|  | Marker | | 120ETB | | 1pack | | -- | | | 120.00 | |  |  |  |
|  | Duplicating paper | | 150ETB | | 3realm | | -- | | | 450.00 | |  |  |  |
|  | Duplication and binding | | 1ETB | | 280Ques | | -- | | | 280.00 | |  |  |  |
| 3 | Carrying bag | | 50ETB | | 12 items | | -- | | | 600.00 | |  |  |  |
| 4 | Data collection cost | |  | |  | |  | | |  | |  |  |  |
|  | Data collection per-diem | | 325.00 | | 10 persons | | 8 days | | | 26000.00 | | 5ques/day |  |  |
|  | Transportation cost | | 50.00 | | 12 persons | | 6 days | | | 3600.00 | |  |  |  |
| 5 | Supervision per-diem | | 325.00 | | 2 persons | | 8 days | | | 5200.00 | |  |  |  |
|  | Sub Total cost | |  | |  | |  | | | 50128.00 | |  |  |  |
| 6 | Enumeration /census/ | | 325.00 | | 10 persons | | 7days | | | 22750.00 | | 12kebeles |  |  |
| ***N.B*** | ***The total cost will be doubled, except enumeration cost, since two surveys will be carried out at the baseline and end line. Therefore, the cost will be 50128.00*2 = 100256 +* 22750** = **123006.00** | | | | | | | | | | | | |  |
|  | 1. Intervention cost (intervention is carried out in 8 clusters for 6 months) | | | | | | | | | | |  | | |
| SN | Budget Category | | Unit Cost | | Multiplying factor | | | | | Total Cost | | Remark | | |
| 1 | Training and pretest cost | |  | | Quantity | | No of days | | |  | |  | | |
|  | Per-diem | | 325.00 | | 7 persons | | 3 days | | | 6825.00 | | 1supervisor | | |
|  | Coffee/tea | | 5ETB | | 7 persons | | 3 days | | | 105.00 | |  | | |
|  | Soft drinks | | 10ETB | | 7 persons | | 3 days | | | 210.00 | |  | | |
|  | Cookies | | 120/kg | | 7 persons | | 3 days | | | 240.00 | | 2kg | | |
| 2 | Stationary cost | |  | | Quantity | |  | | |  | |  | | |
|  | Pen | | 10ETB | | 7 items | | -- | | | 70.00 | |  | | |
|  | Note book | | 25ETB | | 7 items | | -- | | | 175.00 | |  | | |
|  | Pencil | | 5ETB | | 7 items | | -- | | | 35.00 | |  | | |
|  | Eraser | | 5ETB | | 7 items | | -- | | | 35.00 | |  | | |
|  | Case book | | 45ETB | | 7 items | | -- | | | 315.00 | |  | | |
|  | Sharpener | | 10ETB | | 7 items | | -- | | | 70.00 | |  | | |
|  | Flip chart | | 150ETB | | 1 pad | | -- | | | 150.00 | |  | | |
|  | Marker | | 120ETB | | 1pack | | -- | | | 120.00 | |  | | |
|  | Colored paper | | 250ETB | | 2realms | | -- | | | 500.00 | | 3sheets/book | | |
|  | Printing | | 1ETB | | 420sheets | |  | | | 420.00 | | 140 booklets | | |
| 3 | Intervention delivery cost | |  | |  | |  | | |  | |  | | |
|  | Per-diem (4HH/day) | | 325.00 | | 7 persons | | 8dys/round | | | 36400.00 | | 2rounds | | |
|  | Transportation cost | | 50.00/trip | | 7 persons | | 6 dys/round | | | 4200.00 | | 2rounds | | |
|  | Mobile card | | 1ETB/min | | 140clients | | 10minutes | | | 1400.00 | | 10min call | | |
| 4 | Patient screening cost | | 100ETB/cl | | 140clients | | -- | | | 14000.00 | |  | | |
|  | Subtotal cost | |  | |  | |  | | | 65270.00 | |  | | |
|  | 1. Data management cost | | | | | | | | | | |  | | |
|  | Budget Category | | Unit Cost | | Multiplying factor | | | | Total Cost | | | Remark | | |
| 1 | Translation-Questions | | 100ETB/pg | | 9pages | | -- | | 900.00 | | |  | | |
| 2 | Translation-Manual | | 100ETB/pg | | 3pages | | -- | | 300.00 | | |  | | |
| 3 | Coding & data entry | | 5ETB/bk | | 280booklet | | 2rounds | | 2800.00 | | |  | | |
|  | Subtotal cost | |  | |  | |  | | 4000.00 | | |  | | |
|  | **Project 1 Grand total (A+B+C)** | | | |  | |  | | **192276.00** | | |  | | |
|  | | Crosscutting issue for all projects | |  | |  | |  | |  |  | | | |
| 1 | | Fuel | | 20ETB/lit | | 50lit/trip | | 3projects | | 6000.00 | 2trips/project | | | |
| 2 | | Driver’s per-diem | | 325ETB | | 1person | | 5days/trip | | 9750.00 | 2trips/project | | | |
|  | | Subtotal cost | |  | |  | |  | | 15750.00 |  | | | |
|  | | **Project 3 Grand Total cost** | | | |  | |  | | **208026.00** |  | | | |

RESEARCH WORK PLAN (TIME TABLE)

Table 4 Research work plan and timelines

| S.n | Activity | 2021-2022 | | | | | | | | | | | | |  |  |
| --- | --- | --- | --- | --- | --- | --- | --- | --- | --- | --- | --- | --- | --- | --- | --- | --- |
|  |  | Apri | May | Jun | Jul | Aug | Sept | Oct | Nov | Dec | Jan | Feb | Mar | Apr | May | June-Aug |
| 1 | Proposal defense & Ethical clearance |  |  |  |  |  |  |  |  |  |  |  |  |  |  |  |
| 2 | Training of data collectors |  |  |  |  |  |  |  |  |  |  |  |  |  |  |  |
| 3 | Census & baseline data collection |  |  |  |  |  |  |  |  |  |  |  |  |  |  |  |
| 4 | Intervention phase |  |  |  |  |  |  |  |  |  |  |  |  |  |  |  |
| 5 | End line survey |  |  |  |  |  |  |  |  |  |  |  |  |  |  |  |
| 6 | Data entry, analysis & report writing |  |  |  |  |  |  |  |  |  |  |  |  |  |  |  |

# REFERENCES

1. Abate, S. M. (2015) ‘Gynecology & Obstetrics Trends of Cervical Cancer in Ethiopia’. doi: 10.4172/2161-0932.S3.
2. Abdel-Wahab, M. *et al.* (2013) ‘Status of radiotherapy resources in Africa: An International Atomic Energy Agency analysis’, *The Lancet Oncology*, 14(4), pp. e168–e175. doi: 10.1016/S1470-2045(12)70532-6.
3. Abdullah, F. and Su, T. T. (2013) ‘Applying the Transtheoretical Model to evaluate the effect of a call-recall program in enhancing Pap smear practice: A cluster randomized trial’, *Preventive Medicine*, 57(SUPPL), pp. S83–S86. doi: 10.1016/j.ypmed.2013.02.001.
4. Abiodun, O. A. *et al.* (2014) ‘Impact of health education intervention on knowledge and perception of cervical cancer and cervical screening uptake among adult women in rural communities in Nigeria’, *BMC Public Health*, 14(1). doi: 10.1186/1471-2458-14-814.
5. Acera, A. *et al.* (2017) ‘Increasing cervical cancer screening coverage: A randomised, community-based clinical trial’, *PLoS ONE*, 12(1), pp. 1–11. doi: 10.1371/journal.pone.0170371.
6. Adamu, A. N., Abiola, A. O. and Ibrahim, M. T. O. (2012) ‘The effect of health education on the knowledge, attitude, and uptake of free Pap smear among female teachers in Birnin-Kebbi, North-Western Nigeria’, *Nigerian Journal of Clinical Practice*, 15(3), pp. 326–332. doi: 10.4103/1119-3077.100632.
7. Adesina, A. *et al.* (2013) ‘Improvement of pathology in sub-Saharan Africa’, *The Lancet Oncology*, 14(4), pp. e152–e157. doi: 10.1016/S1470-2045(12)70598-3.
8. Arbyn, M. *et al.* (2011) ‘Worldwide burden of cervical cancer in 2008’, *Annals of Oncology*, 22(12), pp. 2675–2686. doi: 10.1093/annonc/mdr015.
9. Arbyn, M. *et al.* (2020) ‘Estimates of incidence and mortality of cervical cancer in 2018: a worldwide analysis’, *The Lancet Global Health*, 8(2), pp. e191–e203. doi: 10.1016/S2214-109X(19)30482-6.
10. Bebis, H. *et al.* (2012) ‘Effect of health education about cervical cancer and papanicolaou testing on the behavior, knowledge, and beliefs of turkish women’, *International Journal of Gynecological Cancer*, 22(8), pp. 1407–1412. doi: 10.1097/IGC.0b013e318263f04c.
11. Bhakta, N. *et al.* (2016) ‘UHC in Africa : A Framework for Action Executive Summary’, *West African journal of medicine*, 35(2), pp. 155–160. doi: 10.1136/archdischild-2011-301419.
12. Black, E. and Richmond, R. (2018) ‘Prevention of cervical cancer in sub-saharan Africa: The advantages and challenges of HPV vaccination’, *Vaccines*, 6(3). doi: 10.3390/vaccines6030061.
13. Bosch, F. X. *et al.* (2001) ‘Papillomavirus research update: Highlights of the Barcelona HPV 2000 international papillomavirus conference’, *Journal of Clinical Pathology*, 54(3), pp. 163–175. doi: 10.1136/jcp.54.3.163.
14. Bray, F. *et al.* (2018) ‘Global cancer statistics 2018: GLOBOCAN estimates of incidence and mortality worldwide for 36 cancers in 185 countries’, *CA: A Cancer Journal for Clinicians*, 68(6), pp. 394–424. doi: 10.3322/caac.21492.
15. Bruni, L. *et al.* (2010) ‘Cervical human papillomavirus prevalence in 5 continents: Meta-analysis of 1 million women with normal cytological findings’, *Journal of Infectious Diseases*, 202(12), pp. 1789–1799. doi: 10.1086/657321.
16. Bruni, L. *et al.* (2019) ‘Human Papillomavirus and Related Diseases in the World- Summary report’, *ICO/IARC Information Centre on HPV and Cancer (HPV Information Centre)*, (June), p. 307. Available at: https://hpvcentre.net/statistics/reports/XWX.pdf.
17. Chinwe R, E. (2015) ‘Impact of Health Education on Knowledge, Attitude and Practice of Cervical Cancer Screening Among Secondary School Teachers in Enugu State’, *Journal of Womens Health Care*, 04(04). doi: 10.4172/2167-0420.1000241.
18. CISN (2014) *Emerging Solution cancer information and support network*.
19. Dehdari, T. *et al.* (2014) ‘Effects of an educational intervention based on the protection motivation theory and implementation intentions on first and second pap test practice in Iran’, *Asian Pacific Journal of Cancer Prevention*, 15(17), pp. 7257–7261. doi: 10.7314/APJCP.2014.15.17.7257.
20. Denny, L. *et al.* (2017) ‘Interventions to close the divide for women with breast and cervical cancer between low-income and middle-income countries and high-income countries’, *The Lancet*, 389(10071), pp. 861–870. doi: 10.1016/S0140-6736(16)31795-0.
21. Denny, L. and Anorlu, R. (2012) ‘Cervical cancer in Africa’, *Cancer Epidemiology Biomarkers and Prevention*, 21(9), pp. 1434–1438. doi: 10.1158/1055-9965.EPI-12-0334.
22. Dulla, D., Daka, D. and Wakgari, N. (2017) ‘Knowledge about cervical cancer screening and its practice among female health care workers in Southern Ethiopia: A cross-sectional study’, *International Journal of Women’s Health*, 9, pp. 365–372. doi: 10.2147/IJWH.S132202.
23. Duncan, K. *et al.* (2019) ‘Challenges and opportunities in the creation and implementation of cancer-control plans in Africa’, *Ecancermedicalscience*, 13, pp. 1–6. doi: 10.3332/ecancer.2019.938.
24. Ebu, N. I. *et al.* (2019) ‘Impact of health education intervention on knowledge and perception of cervical cancer and screening for women in Ghana’, *BMC Public Health*, 19(1), pp. 1–11. doi: 10.1186/s12889-019-7867-x.
25. Erwin, E. *et al.* (2019) ‘SMS behaviour change communication and eVoucher interventions to increase uptake of cervical cancer screening in the Kilimanjaro and Arusha regions of Tanzania: A randomised, double-blind, controlled trial of effectiveness’, *BMJ Innovations*, 5(1), pp. 28–34. doi: 10.1136/bmjinnov-2018-000276.
26. FDRE M OH (2015) ‘Guideline for Cervical Cancer Prevention and Control in Ethiopia’.
27. Ferlay, J. *et al.* (2015) ‘Cancer incidence and mortality worldwide: Sources, methods and major patterns in GLOBOCAN 2012’, *International Journal of Cancer*, 136(5), pp. E359–E386. doi: 10.1002/ijc.29210.
28. FMoH (2015) ‘National Cancer Control Plan 2016 - 2020 of Ethiopia’, *Disease Prevention and Control Directoriate*, (October 2015), p. 83.
29. Fujiwara, H. *et al.* (2015) ‘Effect of providing risk information on undergoing cervical cancer screening: A randomized controlled trial’, *Archives of Public Health*, 73(1), pp. 1–5. doi: 10.1186/s13690-014-0055-7.
30. Gakidou, E., Nordhagen, S. and Obermeyer, Z. (2008) ‘Coverage of cervical cancer screening in 57 countries: Low average levels and large inequalities’, *PLoS Medicine*, 5(6), pp. 0863–0868. doi: 10.1371/journal.pmed.0050132.
31. Gana, G. J. *et al.* (2016) ‘Effect of an educational program on awareness of cervical cancer and uptake of Pap smear among market women in Niger State, North Central Nigeria’, *Journal of Public Health and Epidemiology*, 8(10), pp. 211–219. doi: 10.5897/jphe2016.0849.
32. Ghittoni, R. *et al.* (2015) ‘Role of human papillomaviruses in carcinogenesis’, *Ecancermedicalscience*, 9, pp. 1–9. doi: 10.3332/ecancer.2015.526.
33. Guvenc, G., Akyuz, A. and Yenen, M. C. (2013) ‘Effectiveness of nursing interventions to increase pap smear test screening’, *Research in Nursing and Health*, 36(2), pp. 146–157. doi: 10.1002/nur.21526.
34. Haedicke, J. and Iftner, T. (2013) ‘Human papillomaviruses and cancer’, *Radiotherapy and Oncology*, 108(3), pp. 397–402. doi: 10.1016/j.radonc.2013.06.004.
35. Harvey, N., Adeyoju, A. and Brough, R. (2017) *Prostate cancer in Sub-Saharan Africa: Diagnosis and management*, *Cancer in Sub-Saharan Africa: Current Practice and Future*. doi: 10.1007/978-3-319-52554-9_7.
36. Hoque, M. E. (2010) ‘Cervical cancer awareness and preventive behaviour among female university students in South Africa’, *Asian Pacific Journal of Cancer Prevention*, 11(1), pp. 127–130.
37. Interis, E. C. *et al.* (2015) ‘Increasing cervical cancer awareness and screening in Jamaica: Effectiveness of a theory-based educational intervention’, *International Journal of Environmental Research and Public Health*, 13(1), pp. 1–11. doi: 10.3390/ijerph13010053.
38. Josefsson, A. M. *et al.* (2000) ‘2000 jesefsson HPV VL CxCa’, 355, pp. 2189–2193.
39. Kasamatsu, E. *et al.* (2018) ‘Factors associated with high-risk human papillomavirus infection and high-grade cervical neoplasia: A population-based study in Paraguay’, *PLoS ONE*, 14(6), pp. 1–21. doi: 10.1371/journal.pone.0218016.
40. Kurt, G. and Akyuz, A. (2019) ‘Evaluating the Effectiveness of Interventions on Increasing Participation in Cervical Cancer Screening’, *Journal of Nursing Research*, 27(5), pp. 1–11. doi: 10.1097/jnr.0000000000000317.
41. Lee, H. Y. *et al.* (2014) ‘Mobile phone text messaging intervention for cervical cancer screening: Changes in knowledge and behavior pre-post intervention’, *Journal of Medical Internet Research*, 16(8), p. e196. doi: 10.2196/jmir.3576.
42. Li, J. *et al.* (2015) ‘Effect of a group educational intervention on rural Chinese women’s knowledge and attitudes about human papillomavirus (HPV) and HPV vaccines’, *BMC Cancer*, 15(1), pp. 1–11. doi: 10.1186/s12885-015-1682-2.
43. Linden, A. F. *et al.* (2012) ‘Challenges of surgery in developing countries: A survey of surgical and anesthesia capacity in uganda’s public hospitals’, *World Journal of Surgery*, 36(5), pp. 1056–1065. doi: 10.1007/s00268-012-1482-7.
44. Lou, H. *et al.* (2018) ‘Low-cost HPV testing and the prevalence of cervical infection in asymptomatic populations in Guatemala’, *BMC Cancer*, 18(1), pp. 1–7. doi: 10.1186/s12885-018-4438-y.
45. Love, G. D. and Tanjasiri, S. P. (2012) ‘Using Entertainment-education to promote cervical cancer screening in Thai women’, *Journal of Cancer Education*, 27(3), pp. 585–590. doi: 10.1007/s13187-012-0369-5.
46. Mbachu, C., Dim, C. and Ezeoke, U. (2017) ‘Effects of peer health education on perception and practice of screening for cervical cancer among urban residential women in south-east Nigeria: A before and after study’, *BMC Women’s Health*, 17(1), pp. 1–8. doi: 10.1186/s12905-017-0399-6.
47. Mbamara, S. U. *et al.* (2011) ‘Knowledge, Attitude and Practice of Cervical Cancer Screening Among Women Attending Gynecology Clinics in a Tertiary Level Medical Care Center in Southeastern Nigeria’, *Journal of Reproductive Medicine for the Obstetrician and Gynecologist*, 56(6), pp. 491–496.
48. Morhason-bello, I. O. *et al.* (2013) ‘Cancer Control in Africa 1 Challenges and opportunities in cancer control in Africa : a perspective from the African Organisation for Research and Training in Cancer’, *Lancet Oncology*, 14(4), pp. e142–e151. doi: 10.1016/S1470-2045(12)70482-5.
49. Morhason-Bello, I. O. *et al.* (2013) ‘Challenges and opportunities in cancer control in Africa: A perspective from the African Organisation for Research and Training in Cancer’, *The Lancet Oncology*, 14(4), pp. e142–e151. doi: 10.1016/S1470-2045(12)70482-5.
50. Natphopsuk, S. *et al.* (2012) ‘Risk Factors for Cervical Cancer in Northeastern Thailand: Detailed Analyses of Sexual and Smoking Behavior’, *Asian Pacific Journal of Cancer Prevention*, 13(11), pp. 5489–5495. doi: 10.7314/APJCP.2012.13.11.5489.
51. Ndikom, C. M. *et al.* (2017) ‘Effects of educational intervention on women’s knowledge and uptake of cervical cancer screening in selected hospitals in Ibadan, Nigeria’, *International Journal of Health Promotion and Education*, 55(5–6), pp. 259–271. doi: 10.1080/14635240.2017.1372693.
52. Nigussie, T., Admassu, B. and Nigussie, A. (2019) ‘Cervical cancer screening service utilization and associated factors among age-eligible women in Jimma town using health belief model, South West Ethiopia’, *BMC Women’s Health*, 19(1), pp. 1–10. doi: 10.1186/s12905-019-0826-y.
53. Obulaney, P. A., Gilliland, I. and Cassells, H. (2016) ‘Increasing Cervical Cancer and Human Papillomavirus Prevention Knowledge and HPV Vaccine Uptake through Mother/Daughter Education’, *Journal of Community Health Nursing*, 33(1), pp. 54–67. doi: 10.1080/07370016.2016.1120595.
54. Parkin, D. M. (2006) ‘The global health burden of infection-associated cancers in the year 2002’, 3044(October 2005), pp. 3030–3044. doi: 10.1002/ijc.21731.
55. Parsa, P. *et al.* (2017) ‘Effects of group counseling based on health belief model on cervical cancer screening beliefs and performance of rural women in Kaboudrahang, Iran’, *Asian Pacific Journal of Cancer Prevention*, 18(6), pp. 1525–1530. doi: 10.22034/APJCP.2017.18.6.1525.
56. Paskett, E. D. *et al.* (2011) ‘Evaluating the efficacy of lay health advisors for increasing risk-appropriate pap test screening: A randomized controlled trial among Ohio appalachian women’, *Cancer Epidemiology Biomarkers and Prevention*, 20(5), pp. 835–843. doi: 10.1158/1055-9965.EPI-10-0880.
57. Perkins, R. B. *et al.* (2007) ‘A community-based education program about cervical cancer improves knowledge and screening behavior in Honduran women’, *Revista Panamericana de Salud Publica/Pan American Journal of Public Health*, 22(3), pp. 187–193. doi: 10.1590/S1020-49892007000800005.
58. Petereit, D. G. and Coleman, C. N. (2015) ‘Editorial: “Global challenges in radiation oncology”’, *Frontiers in Oncology*, 5(MAY), pp. 15–18. doi: 10.3389/fonc.2015.00103.
59. Radde, K. *et al.* (2016) ‘Invitation to cervical cancer screening does increase participation in Germany: Results from the MARZY study’, *International Journal of Cancer*, 139(5), pp. 1018–1030. doi: 10.1002/ijc.30146.
60. Randall, T. C. and Ghebre, R. (2016) ‘Challenges in prevention and care delivery for women with cervical cancer in sub-Saharan Africa’, *Frontiers in Oncology*, 6(JUN), pp. 1–7. doi: 10.3389/fonc.2016.00160.
61. Rosen, B. *et al.* (2017) ‘PT US Hills , Michigan United States’, *Gynecologic Oncology Reports*. doi: 10.1016/j.gore.2017.06.014.
62. Rosser, J. I., Njoroge, B. and Huchko, M. J. (2015) ‘Changing knowledge, attitudes, and behaviors regarding cervical cancer screening: The effects of an educational intervention in rural Kenya’, *Patient Education and Counseling*, 98(7), pp. 884–889. doi: 10.1016/j.pec.2015.03.017.
63. Roura, E. *et al.* (2012) ‘Predictors of human papillomavirus infection in women undergoing routine cervical cancer screening in Spain: the CLEOPATRE study’, *BMC Infectious Diseases*, 12. doi: 10.1186/1471-2334-12-145.
64. Rutterford, C., Copas, A. and Eldridge, S. (2015) ‘Methods for sample size determination in cluster randomized trials’, *International Journal of Epidemiology*, 44(3), pp. 1051–1067. doi: 10.1093/ije/dyv113.
65. Ryan, G. W. *et al.* (2014) ‘Downloaded from content.healthaffairs.org by Health Affairs on December 6, 2014 at Kenya: HEALTH AFFAIRS Sponsored’. doi: 10.1377/hlthaff.2013.1155.
66. Sahasrabuddhe, V. V. *et al.* (2012) ‘Cervical cancer prevention in low- and middle-income countries: Feasible, affordable, essential’, *Cancer Prevention Research*, 5(1), pp. 11–17. doi: 10.1158/1940-6207.CAPR-11-0540.
67. Sambo, L. G. *et al.* (2012) ‘Cancer in Africa: A preventable public health crisis’, *Journal Africain du Cancer*, 4(2), pp. 127–136. doi: 10.1007/s12558-012-0212-2.
68. Sankaranarayanan, R. *et al.* (2010) ‘Cancer survival in Africa, Asia, and Central America: a population-based study’, *The Lancet Oncology*, 11(2), pp. 165–173. doi: 10.1016/S1470-2045(09)70335-3.
69. Sankaranarayanan, R. (2014) ‘Screening for cancer in low- and middle-income countries’, *Annals of Global Health*, 80(5), pp. 412–417. doi: 10.1016/j.aogh.2014.09.014.
70. Scheurer, M. E., Tortolero-Luna, G. and Adler-Storthz, K. (2005) ‘Human papillomavirus infection: Biology, epidemiology, and prevention’, *International Journal of Gynecological Cancer*, 15(5), pp. 727–746. doi: 10.1111/j.1525-1438.2005.00246.x.
71. Sharma, P. and Pattanshetty, S. M. (2018) ‘A study on risk factors of cervical cancer among patients attending a tertiary care hospital: A case-control study’, *Clinical Epidemiology and Global Health*, 6(2), pp. 83–87. doi: 10.1016/j.cegh.2017.10.001.
72. Sossauer, G. *et al.* (2014) ‘Impact of an educational intervention on women’s knowledge and acceptability of human papillomavirus self-sampling: A randomized controlled trial in cameroon’, *PLoS ONE*, 9(10), pp. 1–8. doi: 10.1371/journal.pone.0109788.
73. Stanley, M., Lowy, D. R. and Frazer, I. (2006) ‘Chapter 12: Prophylactic HPV vaccines: Underlying mechanisms’, *Vaccine*, 24(SUPPL. 3), pp. 106–113. doi: 10.1016/j.vaccine.2006.05.110.
74. Teame, H. *et al.* (2018) ‘Factors associated with cervical precancerous lesions among women screened for cervical cancer in Addis Ababa, Ethiopia: A case control study’, *PLoS ONE*, 13(1), pp. 1–13. doi: 10.1371/journal.pone.0191506.
75. Tessema, S. *et al.* (2018) ‘Estimates of Cancer Incidence in Ethiopia in 2015 Using Population-Based Registry Data’, *Journal of Global Oncology*. doi: : https://doi.org/10.1200/JGO.17.00175.
76. Thompson, B. *et al.* (2017) ‘Results of a randomized controlled trial to increase cervical cancer screening among rural Latinas’, *Cancer*, 123(4), pp. 666–674. doi: 10.1002/cncr.30399.
77. Torre, L. A. *et al.* (2015) ‘Global cancer statistics, 2012’, *CA: A Cancer Journal for Clinicians*, 65(2), pp. 87–108. doi: 10.3322/caac.21262.
78. Tsu, V. D. *et al.* (2018) ‘Opportunities and challenges for introducing HPV testing for cervical cancer screening in sub-Saharan Africa’, *Preventive Medicine*, 114(March), pp. 205–208. doi: 10.1016/j.ypmed.2018.07.012.
79. Urasa, M. and Darj, E. (2011) ‘Knowledge of cervical cancer and screening practices of nurses at a regional hospital in Tanzania’, *African Health Sciences*, 11(1), pp. 48–57.
80. De Vuyst, H. *et al.* (2013) ‘The burden of human papillomavirus infections and related diseases in sub-saharan Africa’, *Vaccine*, 31(S5), pp. F32–F46. doi: 10.1016/j.vaccine.2012.07.092.
81. WHO (2012) *Projections of mortality and causes of death, 2015 and 2030*.
82. WHO (2013) ‘WHO guidelines for screening and treatment of precancerous lesions for cervical cancer prevention’, *Guideline*.
83. WHO (2014) *Comprehensive Cervical Cancer Control*. 2nd ed, *Guideline*. 2nd ed. Geneva, Switzerland.
84. Wondemagegnhu, T. (2015) ‘Pattern of Cancer in Tikur Anbessa Specialized Hospital Oncology Center in Ethiopia from 1998 to 2010’, *International Journal of Cancer Research and Molecular Mechanisms ( ISSN 2381-3318 )*, 1(1), pp. 1–5. doi: 10.16966/2381-3318.103.
85. Wong, C. L. *et al.* (2019) ‘Effects of a community health worker-led multimedia intervention on the uptake of cervical cancer screening among south Asian women: A pilot randomized controlled trial’, *International Journal of Environmental Research and Public Health*, 16(17). doi: 10.3390/ijerph16173072.
86. World Health Organization (2013) ‘Comprehensive cervical cancer prevention and control: a healthier future for girls and women WHO GUIDANCE NOTE WHO Library Cataloguing-in-Publication Data’, *World Health Organization*, p. 12. Available at: www.who.int/about/licensing/.
87. Yitagesu, H., Samuel, Y. and Tariku, L. (2017) ‘Knowledge, attitude and practice for cervical cancer prevention and control among women of childbearing age in Hossana Town, Hadiya zone, Southern Ethiopia: Community-based cross-sectional study’, *PLoS One*, 12(7), pp. 181–200.

# ANNEXES

Annex 1Participant’s Information and informed consent agreement form /English/

***Project Title***: *CERVICAL CANCER SCREENING IN ETHIOPIA: UNDERSTANDING THE CONTEXTS, DETERMINANTS AND EFFECT OF COUPLE EDUCATION ON KNOWLEDGE, ATTITUDE AND UPTAKE OF CERVICAL CANCER SCREENING SERVICE AMONG WOMEN OF CHILD BEARING AGE IN SOUTHERN ETHIOPIA*

**Information Sheet**

Dear Respondent:

My name is ____________. I am working for the research project entitled “*understanding the contexts, determinants and effect of couple education on knowledge, attitude and uptake of cervical cancer screening service among women of child bearing age in southern Ethiopia*”. The study involves research and its purpose is to determine the effect of couple education on knowledge, attitude and uptake of cervical cancer screening services among women of child bearing age. Participation in this research will benefit individual woman by increasing her knowledge and encourage behavioral change to utilize the screening services. It also gives an opportunity to ask questions and learn more about the topic confidentially after the interview. Currently I am working with the research team of Jimma University to realize such objectives. Now, I am going to interview you & collect information which is required for the research purposes. You are randomly selected to be included in the study as part of the sample population to complete the questionnaire designed by the research team. The information obtained in this study will be used only for research purposes. The information you will provide is helpful to achieve the intended objectives of the study so that you and your community will benefit from. Any information obtained will be kept strictly confidential and will not be exposed to any other body. No personal identifiers will be attached to the information you will provide. Involvement in this study is voluntary and you can drop any individual question or the whole questionnaire at any time without giving a reason. Your refusal to participate or drop out will not produce penalty. However, your participation and contribution in the study is very helpful to come up with important findings to intervene the problem. The questionnaire will probably take 30-40 minutes to complete. For any information you can contact Mr. Samuel Yohannes; phone 0912009158/0933711704

Do you have any opinion regarding this study? Do you agree to participate in this study?

Yes, continue No, thank you!

**Consent form**

I, the undersigned have been informed, in the language I can understand, and understood the purpose of this particular research project. I have been informed that the information I give will be used only for the purpose of this study; my identity, the information I give will be treated confidentially. I have also been informed that I can refuse to participate in the study, not to respond to question if I am not interested or stop responding to question at any time in the process. Based on the above information I agree to participate in the research voluntarily.

Participant’s Sign____________ Person in charge of the informed consent, sign _____________

Annex 2 Questionnaire for cluster randomized controlled trial

We will ask you about general information about your household addresses

| **Household Identification** |
| --- |
| Woreda :_________________Kebele ID: \|____\|____\| household code : \|______\|______\|______\|  Kebele Name________________________________Village Name: ______________________________  Date of interview (dd/mm/yyyy)\|__________\|__________\|___________\|  Time at the beginning of the interview _____:______ |

**SECTION-I: SOCIO-DEMOGRAPHIC CHARACTERISTICS OF THE RESPONDENTS**

We are going to ask you about your sociodemographic characteristics

| **S.N** | **Questions** | **Response and Coding** | **Skip** |
| --- | --- | --- | --- |
| **101** | What is your age in completed years? | ____________years |  |
| **102** | What is your current marital status? | 1. Married 2. Single 3. Divorced 4. Widowed 5. Separated |  |
| **103** | What is your religion? | 1. Protestant 2. Orthodox 3. Muslim 4. Catholic 5. Others (specify)___________ |  |
| **104** | What is your ethnicity? | 1. Kembata 2. Tembaro 3. Hadiya 4. Wolayita 5. Amhara 6. Others (specify)___________ |  |
| **105** | What is your highest educational grade? | __________ grade |  |
| **106** | What is your occupational status? | 1. House wife /domestic roles/ 2. Employee (GO/NGO) 3. Merchant 4. Student 5. Farmer 6. Daily worker 7. Others (Specify)_________ |  |
| **107** | What is the occupation of your husband? (if any) | 1. Employee (GO/NGO) 2. Merchant 3. Student 4. Farmer 5. Daily worker 6. Others (Specify)_________ |  |
| **108** | What is the highest educational status attained by your husband? (if any) | _____________ grade |  |
| **109** | How much is the monthly income of your house hold? (salary, farming, trade, rental etc) | __________ in ETB |  |
| **110** | Are you a member of the health insurance? | 1. Yes 2. No |  |
| **111** | Are you a member of women development army? | 1. Yes 2. No |  |
| **112** | What was your age at your first marriage? | ________years |  |
| **113** | How many times did you give birth? | __________ |  |
| **114** | How many family members do you have? | __________ |  |
| **115** | What is the age of your husband? | ________years |  |

**SECTION II: WOMEN’s KNOWLEDGE ABOUT CERVICAL CANCER**

We are going to ask you about your knowledge regarding cervical cancer

| **S.N** | **Questions** | **Response and Coding** | **Skip** |
| --- | --- | --- | --- |
| **201** | Have you ever heard of the cervical cancer? | 1. Yes 2. No |  |
| **202** | Do you think that woman with multiple sexual partners is at higher risk of getting cervical cancer? | 1. Yes 2. No 3. Do not know |  |
| **203** | Which of the following do you think causes cervical cancer? | 1. Sexually transmitted germs 2. Curse of God 3. Do not know |  |
| **204** | Do you think that any woman who has engaged in sexual relations can be at risk of getting cervical cancer? | 1. Yes 2. No 3. I don’t know |  |
| **205** | Which of the following symptoms is of cervical cancer? (*Multiple response is possible*) | 1. Bleeding in between periods 2. Foul smelling vaginal discharge 3. Pain during sexual contact 4. Heavier and longer periods 5. Do not know |  |
| **206** | Do you think cervical cancer is preventable? | 1. Yes 2. No 3. Do not know |  |
| **207** | Do you know the ways how you can prevent cervical cancer? (*More than one answer is possible)* | 1. Regular screening & treatment 2. Minimizing risky behavior 3. Vaccination og girls at age 9-13 4. Do not know |  |
| **208** | Do you think that early detection of cervical pre-cancer has treatment? | 1. Yes 2. No 3. Do not know |  |

**SECTION III: WOMEN’S KNOWLEDGE ABOUT CERVICAL CANCER SCREENING**

We are going to ask you about your knowledge regarding cervical cancer screening

| **S.N** | **Questions** | **Response and Coding** | **Skip** |
| --- | --- | --- | --- |
| **301** | Have you heard about cervical cancer screening? | 1. Yes 2. No |  |
| **302** | If yes, for 301 where did you get the information from?  (*More than one answer is possible)* | 1. Health workers 2. Mass media 3. Women in the social network (screened/unscreened) 4. Others______________________ |  |
| **303** | Which of the following age groups of women do you think is eligible for screening of cervical pre-cancer in Ethiopia? | 1. Less than 30 years 2. 30 to 49 years 3. 50 years and above 4. Do not know |  |
| **304** | Do you think that married woman like you should have cervical pre-cancer screening? | 1. Yes 2. No 3. Do not know |  |
| **305** | Do you think that cervical pre-cancer screening is more important for woman having multiple sex partners? | 1. Yes 2. No 3. Do not know |  |
| **306** | Which of the following do you think is the advantage of receiving early screening for cervical pre-cancer? (*Multiple answer is possible*) | 1. Early detection of pre-cancer cells 2. Help receive early treatment option 3. Help prevent advanced cancer stage 4. Do not know |  |
| **307** | Do you think that women should receive screening tests only when they experience problems such as pain or vaginal bleeding? | 1. Yes 2. No 3. Do not know |  |
| **308** | Which of the following is the regular screening interval for cervical pre-cancer following normal results? | 1. Every 3 years 2. Every 4 years 3. Every 5 years 4. Do not know |  |
| **309** | At which health facility do you think cervical pre-cancer screening service is being provided? | 1. _______________________ 2. Do not know |  |

**SECTION IV: WOMEN’S CERVICAL CANCER SCREENING PRACTICES**

We are going to ask you about your cervical cancer screening practice

| **401** | Have you ever been screened for cervical pre-cancer? | 1. Yes 2. No | **Skip** |
| --- | --- | --- | --- |
| **402** | If yes for question 401 when was last time you screened? | 1. Within the last 5 years 2. More than 5 years ago |  |
| **403** | If yes for question 401, who initiated you to get screened? | 1. Health professionals 2. Mass media 3. Friends/relatives 4. Others_______________________ |  |
| **404** | If yes for question 401, where did you get the screening service? | 1. Government hospital 2. Private hospital |  |
| **405** | If no for question 401, why haven’t you been screened for cervical pre-cancer? | 1. Lack of awareness /not being informed/ 2. Fear of the procedure & bad results 3. Lack of family support & encouragement 4. Others (distance, stigma) ____________ |  |
| **406** | If you have screened before five years, do you have plan to be screened in the future? | 1. Yes 2. No |  |
| **407** | If not planned in the future, why? | 1. I thought one time test is enough 2. I am busy with other responsibilities 3. I tested negative before 4. Others__________________________ |  |
| **408** | How likely do you think that you will develop cervical cancer disease? | 1. Likely 2. Not likely |  |

**SECTION V: WOMEN’s ATTITUDE ABOUT CERVICAL CANCER & ITS SCREENING**

We are going to ask you about your attitude & beliefs regarding cervical cancer screening and its uptake

| **S.N** | **Questions** | **Response Categories** | | |
| --- | --- | --- | --- | --- |
|  |  | **Agree=1** | **Neutral=2** | **Disagree=3** |
|  | **Perceived susceptibility towards the disease (***an individual’s subjective perception of being at risk of cervical cancer***)** | | | |
|  | Women are very worried of getting cervical cancer when they get pain in their womb & vaginal discharge | Agree | Neutral | Disagree |
|  | Women should not worry about cervical cancer because God protects them | Agree | Neutral | Disagree |
|  | Women believe that any woman can acquire cervical cancer in their lifetime | Agree | Neutral | Disagree |
|  | If women look healthy, they will not get cervical cancer | Agree | Neutral | Disagree |
|  | Some women feel that they are likely to get cervical cancer in the future | Agree | Neutral | Disagree |
|  | **Perceived severity of the disease (***subjective evaluation of an individual to perceive the seriousness or the possible consequences of cervical cancer***)** | | | |
|  | Women believe that cervical cancer is serious disease & affects daily activities | Agree | Neutral | Disagree |
|  | Women feel that having cervical cancer does not put a woman to grave | Agree | Neutral | Disagree |
|  | Cervical cancer worries woman about missing her social network with her friends | Agree | Neutral | Disagree |
|  | Women feel that there is no medical treatment and cure for cervical cancer | Agree | Neutral | Disagree |
|  | Women feel that cervical cancer would threaten a relationship with their husbands | Agree | Neutral | Disagree |
|  | Women who experience cervical cancer would suffer for a longer time | Agree | Neutral | Disagree |
|  | **Perceived benefit (***the subjective perception of the benefits of engaging in cervical cancer screening to prevent cervical cancer***)** | | | |
|  | Women believe that having cervical pre-cancer screening is the best option to identify the disease early | Agree | Neutral | Disagree |
|  | Women feel that their participation in screening cannot help prevent cervical cancer disease | Agree | Neutral | Disagree |
|  | Women believe that being screened for cervical pre-cancer benefit them through early treatment options | Agree | Neutral | Disagree |
|  | Women feel that having cervical pre-cancer screening would make difference even in the absence of any symptom | Agree | Neutral | Disagree |
|  | Women feel that having regular cervical pre-cancer screening will not decrease their chances of dying from cervical cancer | Agree | Neutral | Disagree |
|  | Women believe that having regular cervical pre-cancer screening decreases worry about cervical cancer | Agree | Neutral | Disagree |
|  | **Perceived barrier (***factors perceived to be hindering one’s ability to engage in cervical cancer screening or overcoming possible factors associated with seeking cervical cancer screening***)** | | | |
|  | Women believe that screening would not be helpful, since cervical cancer has no cure | Agree | Neutral | Disagree |
|  | Women do not want to get screened because they feel the procedure is painful | Agree | Neutral | Disagree |
|  | Women are ashamed of lying on examination bed and show their private parts to have a cervical pre-cancer screening | Agree | Neutral | Disagree |
|  | Women feel that their husband might not be willing & interested when they expose their private body parts | Agree | Neutral | Disagree |
|  | Women feel busy with their family responsibilities to go to the health facility for screening services | Agree | Neutral | Disagree |
|  | Women are afraid of having a cervical pre-cancer screening for fear of a bad result & social stigma | Agree | Neutral | Disagree |
|  | Women may have other problems more important than having a cervical pre-cancer screening in their life | Agree | Neutral | Disagree |
|  | There is no health facility nearby to have a cervical pre-cancer screening | Agree | Neutral | Disagree |
|  | **Cues to action (***the stimulus needed to trigger the decision-making process to take a recommended health action***)** | | | |
|  | I understand that early detection provides chance of cure | Agree | Neutral | Disagree |
|  | I know where to get screened for cervical pre-cancer | Agree | Neutral | Disagree |
|  | **Perceived self-efficacy (***the woman’s level of confidence to engage in cervical cancer screening***)** | | | |
|  | I feel confident enough to get screened for cervical pre-cancer | Agree | Neutral | Disagree |

Name of data collector __________________________________ sig.__________ date _______

Name of Supervisor ____________________________________ sig. __________ date _______

Annex 3 Health Education Brochure /English Version/

**“Cervical Cancer Screening Saves Women’s Lives”**

Educational brochure prepared to increase cervical cancer knowledge and promote positive attitude and screening uptake among eligible women

**Section One**

**What is cervical cancer?**

Cervical cancer is a type of cancer that occurs in the tissues of the cervix — the organ in the lower part of the uterus and the entrance to the uterus from the vagina. Various strains of the human papillomavirus (HPV), a sexually transmitted infection, play a role in causing most cervical cancer.

**Current disease burden (incidence & mortality)**

Nearly 6,300 new cases are diagnosed annually, and about 4,884 women die from cervical cancer each year in Ethiopia. This makes cervical cancer the second-most common cancer in the country, and the second-most deadly cancer among Ethiopian women.

**Section Two**

**Risk factors for cervical cancer**

The risk of developing cervical cancer markedly increases with infection with HPV, the number of lifetime sexual partners, co-infection with other sexually transmitted infections, history of smoking, younger age before 18 at first intercourse and at first pregnancy, high parity 4 and more children, and long-term use of oral contraceptives.

**Signs, symptoms and complications of cervical cancer**

During precancerous stage cervical cancer may have no symptoms. But symptoms of early stage cervical cancer may include: Irregular light bleeding between periods in women of reproductive age; bleeding after sexual intercourse; increased vaginal discharge, sometimes foul smelling; pelvic pain or pain during sexual intercourse. Some of the complications that can occur in advanced cervical cancer are severe bone or muscle pain, kidney failure, blood clots, bleeding, fistula etc.

**Methods of prevention**

Cervical cancer is one of the most preventable female cancers. It can be prevented by vaccination and cervical screening methods. Two vaccine doses provided six months apart are helpful for adolescents aged 14 years in Ethiopia. It is not helpful once a woman has been engaged in sex.

**Section Three**

**Benefits of screening**

Regular screening is the most important practice you can perform to prevent cervical cancer. Screening tests detect precancerous cells in the cervix, which can be treated before cancer ever develops. In fact, most invasive cancers are found in women who have not had regular screening.

**Screening Eligibility and Schedule**

Screening of cervix for pre-cancer cells is recommended for women aged 30 to 49 years old who have been engaged in sexual relations. Every woman has the right to be screened for cervical cancer at least once in her lifetime. But the regular schedule for cervical cancer screening is every five years.

**Barriers to screening /fears or stigma associated with screening/**

The major factors identified by the women that influence screening utilization were lack of knowledge about the need for cervical screening, fatalistic attitudes about cervical cancer, low perceived susceptibility, having many contending issues, financial constraint, and emotional barriers like fear of having a positive result, embarrassment and anticipated shame, lack of time, absence of symptoms. Counseling will be tailored & focuses on specific barriers to each woman.

**Section Four**

**Meaning of screening results**

The available screening technique in Ethiopia is VIA. When VIA is positive it means the woman has developed abnormal pre-cancerous cells of the cervix. When VIA is negative it means the woman did not develop any abnormal cervical pre-cancer cells. This does not guarantee future condition.

**Treatment options**

The treatment option for pre-cervical cancer is cryotheraphy. But once it advances treatment depends on the stage of the cancer and options include surgery, radiotherapy and chemotherapy. Palliative care is also an essential element of cancer management to relive unnecessary pain and suffering due the disease.

**The cost of screening & where to get the service**

Cervical cancer screening is free of charge. It is available at nearby public health facilities during the working days. The Health facilities currently providing screening services are Mudula primary hospital and Dr. Bogalech Gebre general hospital.

**Importance of male involvement in woman’s cervical cancer screening uptake**

As you know, males are major decision makers in our society on family matters. Conversely females are voiceless majority of the population. Also, women are biologically more vulnerable for different health problems including cervical cancer. They need close support and encouragement from their husbands. Cervical cancer disease is associated with social and economic problems not only for the woman but also a burden for the husband and the family as a whole. Without the participation of males, it is difficult for women to get screening services by their own to early detect the disease and prevent death. Therefore, your wife needs your help & support to get cervical cancer screening service provided in the nearby health facility.

***N.B****: Ask key questions in each section to check wome’s comprehension.*

*Key messages to summarize each session: ‘Screening is the key to preventing cervical cancer’, ‘Women aged 30 and over are most at risk for cancerous changes’ and ’Screening saves women’s lives’.*

*Contact person in charge of the intervention: Samuel Yohannes (Cell phone: 0912009150)*

**Data collection tool in local language**

አኔክስ 1ሴሬኬት መረጀ አስታ መናካ አሴኖ መረጀሀ አጌኖ እተ ውለ /ከምባትሰ ላጋን/

***ፕሮጄክት ሱዕመ***: ማህፀን ከንሰርን እለንሰት ዮ ጠዋከተ፣ ማህፀን ከንሰርነ ምርመርስተኔ ሜንቱ ተማርሱ ሜንት ደጎን፣ ጡደንቻኒ ምርመረ አቅ ዱሀን ኤወኖ አነኖመተ ጡዲ ወሮ ባድ ብሔረሰብነ ምናደብ ክልላን ሁጀቴኖ ሴሬኬቱ

**ሜንቲ አሴኖ መረጅ ዱሀተ**

እትሰት ፈንቀሻንቹት:

ኢ ሱዕሙ ____________ የመመኖ. አን ማህፀን ከንሰርን እለንሰት ዮ ጠዋከተ፣ ማህፀን ከንሰርነ ምርመርስተኔ ሜንቱ ተማርሱ ሜንት ደጎን፣ ጡደንቻኒ ምርመረ አቅ ዱሀን ኤወኖ አነኖመተ ጡዲ ወሮ ባድ ብሔረሰብነ ምናደብ ክልላን ሁጀቴኖ ሴሬኬቲ ሁጀተዮሜ፡፡ ሴሬኬትስ ዋኑስ መሽከኡ ማህፀን ከንሰርን እለንሰት ዮ ጠዋከተ አነነ አሴን ደጊሀ፣ ሞስስነ ምርመር ተኔ ሜንቱ ተማርሱ ኤወኖ አነኖመተ ጡዲሀት፡፡ ከን ሴሬኬቶን አጌን ቆደ ፉሸቁ ሜንትስ ደጉ ሌዕሲችን ህግ ኬእ መረመረንቱንተ አመል ዶር እግመት አሰኖሰ፡፡ አሞ ጠዊስ ተኔ አጉንቡሩ ዮሰደ ጠዕመቱቲ ፈንቀሹት ጎፎ አኒች ዘኪን ጠዕምት ሮሸት አቅቱንተ ሳመ ቆጨኖሰ፡፡ ከ ጄቾን አን ሁጅስ ዘካንቹ እልሲ ጅም ዩንቨርሲቴ ሴሬኬትስ ጠጵቲን ሜጦመን ሁጀተዮም፡፡ ቴሱ አን ሴሬኬቲሀስ ካዕለኖ መረጀ ጠጰኦተ ኬስ ጠዕሞኬታት፡፡ ሴሬኬቶንተስ አጉንታ ጠዕመቶሀስ መረጀ አቱንታ አት ሳምን መረጠንቴንትተ መንቹ፡፡ አት አታንት መረጁ ሴሬኬቲ ጠሊ ሁጄን ሆስሲ አቄኗ፡፡ አት አታንት መረጁ ሴሬኬቶስ ዓላመተ ኢልሲሀ ሂከኒች ዘኪኒ ኬሳሀ ከ አከባቤ መኒሀ ካለቱተ ኤበኗ፡፡ አት አስታንት መረጁ አብሽ ምስጥሪን ቆረበመኗኒ አዬሀ ህግ አሰሙምቧ፡፡ ሱሙሁ መኖመት ኩለኖ መረጁሁ ከ ወረቀት አሌን ፃፈሙምቧ፡፡ ከን ሴሬኬቶን ቆደ አቁ ኢቴን አቄኗኒ፣ሀተ ጠዕመቱታ ፈንቀሹ ሆጊ ደንዴኗኒ ሀከ ሰዓተሀ መሽከአስ ኩሌኑዕነ አጉሬን ፉሊ ደንዴኗ፡፡ ሴሬኬቶን አጉ ድቡሁ አጉሬን ፉሉሁ ሜጡ ቅጣት ኤበኖበአ፡፡ ጠው እኮዳ ሴሬኬቶንተስ አጉክ መለ አጴን ዋልሃ ሀወስ ከሚሀ አብሽ ካዕለኗ፡፡ ጠዕመቱስ ጦንፈን ቀጤ ሰጀዱሚች ሾለዱመ ደቂቀ ጠዕመኖ፡፡

ሀሶት መረጀ ጠዕምት አቂ አቶ ሳሜኤል ዮሐንሴ ጠዕሚ ደንድታንት፣ ስልኪ ቁጡሩ 0912009158

ሴሬኬትስ ተኔ ሰዊቱ ዮሄንዶ? ከ ሴሬኬቶን ቆደ አቂ እትተንዶ?

አ እታም, ቀጠል አይ, ገለጣም!

**ላገተ አጌኖ ፎርመ**

ከኒች ወሮዲን ፈረሞሚት ሴሬኬትስ ዱሀተ ኢ ላጋን ኩሌናን አጌኤ፡፡ አን አሳም መረጁ ሴሬኬቲ ጠሊ ሆሰኖገ ኩሌማኤ፡፡ ኢ ሱዕሙሁ መኖመቲ ደገንቱዕነ አሳም መረጀስ ምስጥሪን ሁጄ አሌን ሆስሴኖገ ኩሌማኤ፡፡ አሞ ሴሬኬቶንተስ አጉ ሆጊ ደንዳምገ፣ ግባም ጠዕመቶሀ ፈንቀሹተ አሱ ሆጊ ደንዳምጋ ሀከ ሰዓተሀ አጉር ፉሊ ደንዳምገ ኩሌማኤ፡፡ ህከን ተኔ ገግዕ እታን ከ ሴሬኬት አዜን አጊ እተሜም፡፡

ሜንትቾስ ፍርመ____________ ሜንትቾሀንስ መረጀ አሴ መንች ፍርመ _____________

**አኔክስ 2** **እንቴርቬንሺን ሴርኬት ጠዕመቱተ** **/ከምባትሰ ላጋን ቅጠንቴዕተ/**

| **ቤተሰቡ ለለኖቤቹ** |
| --- |
| **ወረደ :_________________ቀበሌ ኮደ**: \|____\|____\| ቤተሰብ ኮደ : \|____\|____\|\|____\|  ቀበሌ ሱዕመ________________________________ሰፈር ሱዕመ: ______________________________  መረጀ መጤኖ በር (dd/mm/yyyy)\|__________\|__________\|\|___________\|  መረጀ መጡ ጀመር ሰዓተ _____:______ |

**ሴክሺን መቶ: ጠዕመንታ መንቾ ሶሲዮዴሞግራፌ ዱዱቡ**

| **ዎሉተ** | **ጠዕመቱተ** | **መልሰሃ ኮደሃ** | **ህግ** |
| --- | --- | --- | --- |
| **101** | መንቾ ኡሙር ወጌ ዎሎን ሜኦት? | ____________ወጋ |  |
| **102** | አግጭንች ዱዱቡ ሀትጉተ? | 1. አግሴታ 2. አግሱንቡተ 3. ህዕረንቴታ 4. ምንሴ አኑ ሬሔታ 5. አነነ እክ ሄኣታ |  |
| **103** | ሀይማኖቱዕኔ መሃን? | 1. አመዕናንቹተ 2. ኦርቶዶክስቹተ 3. እስላንቹተ 4. ካቶሊክቹተ 5. ወሉ እኬደ___________ |  |
| **104** | ብሔረሰቡ መሃን? | 1. ከምባትቹተ 2. ጠምበርቹተ 3. ሀዲቹተ 4. ዎላንቹተ 5. አማርቹተ 6. ወሉ እኬደ___________ |  |
| **105** | ሜኡ ክፍለ ዕለንቀጤ ሮሴንታ? | _____________ ክፍለ |  |
| **106** | ዋነት ሁጅዕኔ መሃን? | 1. ምንተ አመ 2. ቀጠረንት ሁጀታታ 3. ዘዘላንቹተ 4. ሮሳንቹተ 5. ገበሬቹተ 6. በሬ ሁጅተ ሁጀታታ 7. ወሉ እኬደ ፃፍ __________ |  |
| **107** | ምንዕኔ አን ሁጅት መሃን? | 1. ቀጠረም ሁጀተኗ 2. ዘዘላንቿ 3. ሮሳንቿ 4. ገበሬቿ 5. በሬ ሁጀታንቹ 6. ወሉ ዮደ_________ |  |
| **108** | ምንዕኔ አኑ ሜኡ ሮሴማሃ? | _______________ ክፍላ |  |
| **109** | ቤተሰብ አገን ገብት ሀባንካ? | __________ ቶጴ ብረ |  |
| **110** | ፈይመ እንሹራንሳን አጌንታእንዶ? | 1. አ አንጌም 2. አንግምበአ |  |
| **111** | ሜንት ልማት ቡዱናን ሀንቀፈንቴንተዕንዶ? | 1. አ ሀንቀፈሜም 2. ሀንቀፈምምበአ |  |
| **112** | ሜኤ ዕድሜኔት አግሴንታእ? | ________years |  |
| **113** | ሜእተ ኦሱተ እልቴንታእ? | ________years |  |
| **114** | ቤተሰብ ወሉት ሜኦት? | ________years |  |
| **115** | ምንዕኔ አን ዕድሜት ሜኤት? | ________years |  |

**ሴክሺን ለሞ፡ ማህፀን ሉድ ከንሰር ዱዱቡ ደግ ዱሀተ**

| **ዎሉተ** | **ጠዕመቱተ** | **መልሰሃ ኮደሃ** | **ህግ** |
| --- | --- | --- | --- |
| **201** | ማህፀን ሉድ ከንሰር ተኔ መጮት ከሰንዶ? | 1. አ መጮጬም 2. መጮጭምበአ |  |
| **202** | ሆለመ ጎኒን ኦሰኣ ሜንቱ ማህፀን ሉድ ከንሰር ሞሶን አብስ ኡባዕንዶ? | 1. ገርተ 2. ቆጰና 3. ደጋምበአ |  |
| **203** | ማህፀን ሉድ ከንሰረ መሃን ዋሸኖ? | 1. ጎኒን ኦሳዕን ዋልታ ጀርማከት 2. መገን አሙርት 3. ደጋምበአ |  |
| **204** | ጎኒን ኦሳኦ ሀትት ሜንትቹቲ ማህፃን ሉድ ከንሰር ሞሶን ኡቢ ደንድታእ? | 1. ገርተ 2. ቆጰና 3. ደጋምበአ |  |
| **205** | ማህፀን ሉድ ከንሰር ሞስ ምልክቱ መሃን?  (መቲች አበተ ፈንቀሹተ አሲ ደንድታንት) | 1. ወግ ሜሬሮን ቄጉ ፉለኖ 2. ማህፀኒች ቦበኖሩ ፉለኖ 3. ኤጨቀንች ሰዓተ ጥደኖ 4. ኬዕማሹ ወጉ ሆለመ በር ፉለኖ 5. ደጋምበአ |  |
| **206** | ማህፀን ሉድ ከንሰረ ከሚ ደንዴኖንዶ? | 1. አ ከሚ ደንዴኖ 2. ከሚ ደንዴኖበአ 3. ደጋምበአ |  |
| **207** | ማህፃን ሉድ ከንሰረ ሀትተ አሴን ከዕሚ ደንዴኖ? (መቲች አበተ ፈንቀሹተ አሲ ደንድታንት) | 1. ጄቾን ጄቾን መረመረሚን አከመሚኒ 2. ሞሶን ኡጂታ ጠዋካች ቆረጲን 3. 14 ዕድሜን ክትባት አቂን 4. ደጋምበአ |  |
| **208** | ማህፀን ሉድ ከንሰሪ ጄቾን ደጌመደ ህክምኑ ዮሀንዶ? | 1. አ ዮሃ 2. ዮበኤ 3. ደጋምበአ |  |

**ሴክሺን ሰሶ፡ ማህፀን ሉድ ከንሰር ምርመር ዱዱቡ ደግ ዱሀተ**

| **ዎሉተ** | **ጠዕመቱተ** | **መልሰሃ ኮደሃ** | **ህግ** |
| --- | --- | --- | --- |
| **301** | ማሕፀን ሉድ ከንሰር ምርመር ተኔ መጮት ከሰንዶ? | 1. አ 2. መጮጭምበአ |  |
| **302** | መጮቴንታደ ሀኖች መጮቴን? | 1. ፈይመ ሁጀታኒች 2. ሬዶኒቺ ቴሌቪጂኒቺ /Mass media/ 3. ደጋም ሜንቲች (screened/unscreened) 4. ወሉ ዮደ______________________ |  |
| **303** | ሜኦ ኡሙሮን ዮ ሜንቱ ማሕፀን ሉድ ከንሰሪ መረመረሚ ደንድታ? | 1. 30 ወጌች ዎሮዱ 2. 30 - 49 ወጌ አዜን ዮሩ 3. 50 ወጌች አብቶሩ 4. ደጋምበአ |  |
| **304** | አግስ ሄኣ ኪ ደረቡ ሜንቱ ማህፀን ሉድ ከንሰር ምርመረ አቁ ሀስሰኖሰንዶ? | 1. አ አቁ ሀስሰኖሰ 2. ሀስሰኖሰበአ 3. ደጋምበአ |  |
| **305** | ሆለመ ጎኒን ኦሳኣ ሜንቲ ምርመረ አቁ አብሽ ሀስሰኖሰ ይት ሰዊተንዶ? | 1. አ ሰዊያም 2. ሰዊያምበአ 3. ደጋምበአ |  |
| **306** | ጄቾን ምርመረ አቅ ካለቱት መሃን ይተን?  (መቲች አበተ ፈንቀሹተ አሲ ደንድታንት) | 1. ከንሰሩ ጀመሩዕነን ጄቾን ደጊ ካዕለኖ 2. ጄቾን ህክምነ አቄን ፈዪ ካዕለኖ 3. ከንሰሩ ኬዕማሸ ደረጀ ዕሉምቦገ ከሚ ካዕለኖ 4. ደጋምበአ |  |
| **307** | ምርመረ አቁ ሀስሰኖሰ ሜንቱ ማህፀን ጥደቲ ዮሰሩ ማህፀኒች ቄጉ ፉለኗ ሜንቱ? | 1. አ ገርተ 2. ገርተበአ 3. ደጋምበአ |  |
| **308** | ሜሜኦ ወጋኔት ሜንቱ ማህፀን ሉድ ከንሰሪ ምርመረ አሱ ሀስሰኖሳ? | 1. ሰሰሶ ወጋን 2. ሾሾሎ ወጋን 3. ኦኦንቶ ወጋን 4. ደጋምበአ |  |
| **309** | ማህፀን ሉድ ከንሰር ምርመረ ሀከኔኔት አሴኖ? | 1. ዱራሜ ሆስፒታላን 2. ሙዱል ሆስፒታላን 3. ወሉ ዮደ___________________________________ 4. ደጋምበአ |  |

**ሴክሺን ሾሎ፡ ማህፀን ሉድ ከንሰር ምርመር ዱሀተ ምን አናከ ካለቱተ**

| **401** | ከኒች ብሬ ማህፀን ሉድ ከንሰር ምርመረ አቅቴን ከሰንዶ? | 1. አ 2. አቅምበአ |  |
| --- | --- | --- | --- |
| **402** | ከኒች ብሬ አቅቴንታደ ሀከዳት? | 1. ኦንቶ ወጌች አዚን 2. ኦንቶ ወጌች ህጌእ |  |
| **403** | ምርመረ አቅቴንትደ አዬት መረመረሜ ይ ኩሌዕኔ? | 1. ሀኪመተ 2. ራዶንች/ ቴሌቪዢኒች መጮጭ /Mass media/ 3. ኢ ደረቡ ኩልቴን 4. ወሉ ዮደ ______________________ |  |
| **404** | ምርመረ አቅቴንተደ ሀከኔ አቅቴን? | 1. መንግስት ሆስፒታላን 2. ግል ሆስፒታላን |  |
| **405** | ከኒች ብሬ መረመረንቴን ከሰበዕደ፤ ምሃት ምርመረስ አቅቴንበእ? | 1. ምርመርስተኔ ደጉ ሆጊን 2. ምርመረስ ውጤተሲ ወጃምተኔ 3. ቤተሰቡ ድክሱ ሆጊን 4. ወሉ ዮደ___________________________ |  |
| **406** | መረመረንቴንታች ኦንቶ ወጌች ህጌደ ከኒች ዘኪን መረመረሚ ሰዊቴንተንዶ? | 1. አ 2. ሰዊ ይምበአ |  |
| **407** | መረመረሚ ሰዊቴኑምቦደ, ሚሃት ሰዊቴንበዕ? | 1. ከኒች ብሬዊ እሀኖ ይኔት 2. ምን አዜን ሁጅት በተአ ብኪሃት 3. ከኒች ብሬ ነፀተ ዬመቢሃት 4. ወሉ ዮደ__________________________ |  |
| **408** | ማህፀን ሉድ ከንሰሩ አፈኖኤ ይቴን ሀወንከ ሰዊቴነን? | 1. ሰዊያም 2. ሰዊያምበአ |  |

**ሴክሺን ኦንቶ፡ ማህፀን ሉድ ከንሰር ተኔ ሜንት መጮጨንቸሃ አመንጨንቸሃ /WOMEN’s ATTITUDE ABOUT CERVICAL CANCER & ITS SCREENING/**

| **ዎሉተ** | **ጠዕመቱተ** | **መልሰሃ ኮደሃ** | | |
| --- | --- | --- | --- | --- |
|  |  | ዕተማም=1 | ሜሬራንቹተ=2 | ዕተማምበአ=3 |
|  | **ሞሶንተስ ኡብ ዱሀተ /*Perceived susceptibility*/** |  |  |  |
|  | ማህፀን ጥደቲ ቦበኖሩ ቆጨሞ ሳተ ሜንቱ ማህፀን ሉድ ከንሰር አፎኤታትንዶ ይት አብስ ክቼአእ /ህከኒ አት መ ይተን/ | ዕተማም | ሜሬራንቹተ | ዕተማምበአ |
|  | መገኑ ቆረበኖሰተኔ ማህፀን ሉድ ከንሰሩ አፈኖ ይት ሜንቱ ክቼኡ ሀስሰኖሰበአ | ዕተማም | ሜሬራንቹተ | ዕተማምበአ |
|  | ሜንትቹተ መንቹተ ማህፀን ሉድ ከንሰሩ ሀከዳ አፊ ደንደኖ ይት ሜንቱ አመዕንታእ | ዕተማም | ሜሬራንቹተ | ዕተማምበአ |
|  | ጥደት መጮጨንቱምቦች ማህፀን ሉድ ከንሰሩ ሜንቱ አፊ ደንዴኖበአ | ዕተማም | ሜሬራንቹተ | ዕተማምበአ |
|  | ማህፀን ሉድ ከንሰሩ ብርተ ለቅ አፊ ደንደኖኔ ይት መመቱ ሜንቱ ወጅታእ | ዕተማም | ሜሬራንቹተ | ዕተማምበአ |
|  | **ሞስስ ኬዕማሽመተኔ መጮጨመኖሰረ /*Perceived severity of the disease*/** |  |  |  |
|  | ማህፀን ሉድ ከንሰሩ ኬዕማሻ እኮተኔ ሁጅተ ከመኖ ይት ሜንቱ አመዕንታእ | ዕተማም | ሜሬራንቹተ | ዕተማምበአ |
|  | ሜንቱ ማህፀን ሉድ ከንሰር ሞሲን ሬናምበአ ይት ሰዊታዕ | ዕተማም | ሜሬራንቹተ | ዕተማምበአ |
|  | ማህፀን ሉድ ከንሴር ሞሱ ሜንት ሜጦመተ ጎደአኖተኔ ማህበራዌ ሄቸሴ ሆጋተኔ ክቼኢ ደንድታእ | ዕተማም | ሜሬራንቹተ | ዕተማምበአ |
|  | ሆለሙ ሜንቱ ማህፀን ሉድ ከንሰሩ ህክምኒን ፈዩምቧ ይት አመዕንታእ | ዕተማም | ሜሬራንቹተ | ዕተማምበአ |
|  | ማህፀን ሉድ ከንሰር ሞሱ ምኒሰ መንቺን ዮ ሜጦመተ ጎደአኖ ይት ሜንቱ አብስ ዋጅታእ | ዕተማም | ሜሬራንቹተ | ዕተማምበአ |
|  | ማህፀን ሉድ ከንሰሩ ዮ ሜንቱ ሆለመ ወጋ ሞስንተስ ሀዊታእ | ዕተማም | ሜሬራንቹተ | ዕተማምበአ |
|  | **ምርመሩስ አሰኖ ካለቶ ዱሀተ /Perceived benefit/** |  |  |  |
|  | መረመረሜን ጡደቁ ሞሱስ አበ ደረጀ እሉዕነ ጄቾን ደቂ ካዕለኖ ይት ሜንቱ አመዕንታእ | ዕተማም | ሜሬራንቹተ | ዕተማምበአ |
|  | ሜንቱ ምርመራ አሰቁሰ ማህፀን ሉድ ከንሰረ ከዕመሚ ካዕለኖበአ ይት ሰዊታእ | ዕተማም | ሜሬራንቹተ | ዕተማምበአ |
|  | ማህፀን ምርመረ አሱ ጄቾን ህክምነ አቂ ካዕለኖ ይት አመዕንታዕ | ዕተማም | ሜሬራንቹተ | ዕተማምበአ |
|  | ሜንቱ ጥደ ስሜቱ ዮበእች ምርመረ አሱስ ሆጉስ ሜጡ ካለቱት ዮበዕ ይት ሰዊታዕ | ዕተማም | ሜሬራንቹተ | ዕተማምበአ |
|  | ሜንቱ ጄቾን ጄቾን ማህፀን ምርመረ አሱ ሞሲንተስ ሬሄኖ ሳመ ቀነሰኖበአ ይት አመዕኒታዕ | ዕተማም | ሜሬራንቹተ | ዕተማምበአ |
|  | ሜንቱ ጄቾን ጄቾን ማህፀን ምርመረ አሱ ሞስስ ተኔ ክቼኤኖ ክቼቸ ቀነሰኖ ይት ሰዊታዕ | ዕተማም | ሜሬራንቹተ | ዕተማምበአ |
|  | **ምርመር ጉፍቹ ዕካ ጠዋካተኔ አማከ አመዕነቱ /Perceived barrier/** |  |  |  |
|  | ሜንቱ ማህፀን ሉድ ከንሰር ሞሱ ፈዩምቦ ተኔ ምርመረ አሱስ ሜጡረ ካዕለኖበአ ይታእ | ዕተማም | ሜሬራንቹተ | ዕተማምበአ |
|  | ሜንቱ ማህፀን ምርመረ አሴኖጃተ ጥደኖ ይታ ብኪ ምርመረስ አቂ ሀሳበአ | ዕተማም | ሜሬራንቹተ | ዕተማምበአ |
|  | ሜንቱ ሀኪም ምን መዕነ አሌን ኦሰዕ ፎልሰ እረ ጡድሱ ሳልስሰኖሰተኔ ምርመረስ አቂ ሀሳበአ | ዕተማም | ሜሬራንቹተ | ዕተማምበአ |
|  | ሜንቱ ምርመረ አስታ ጄቹተ አልሰ እረ ቀዕርታ ብኪ ምንሰ አኑ በጅገኖበአ ይት ምርመረስ አቂ ሀሳበአ | ዕተማም | ሜሬራንቹተ | ዕተማምበአ |
|  | ሜንቱ ምን ሁጄን መቴመተ እኮገ ሰዊታተኔ ማህፀን ምርመረ አሲ ሀኪም ምን መርታበአ | ዕተማም | ሜሬራንቹተ | ዕተማምበአ |
|  | ሜንቱ ምርመርስ ውጤተሀ ዋለኖ ጬመሙሃ ወጅታብኪ ማህፀን ምርመረ አሲ ዋጅታእ | ዕተማም | ሜሬራንቹተ | ዕተማምበአ |
|  | ሜንቱ ማህፀን ምርመሪች ባሼ ብርሰኖ ጠው ሄቻንተሰ ሄአኖ ብኪ ምርመረስ አቂ ሀሳበአ | ዕተማም | ሜሬራንቹተ | ዕተማምበአ |
|  | ሜንቱ ምርመረስ አቄኖ ሀኪም ምኑ ኦንጠኔ ዮበእተኔ ምርመረስ አቂ ሀሳበአ | ዕተማም | ሜሬራንቹተ | ዕተማምበአ |
|  | **ሜንቱ ምርመረስ አቅቱንተ ቃግሳ ጠዋከተ /Cues to action/** |  |  |  |
|  | ማህፀን ሉድ ከንሰር ምርመረ ጄቾን አሱ ሞሲችስ ቆረበኖገ ደጌም | ዕተማም | ሜሬራንቹተ | ዕተማምበአ |
|  | ማህፀን ሉድ ከንሰር ምርመረ ሀከኔ አቄኖንዶ ደጌም | ዕተማም | ሜሬራንቹተ | ዕተማምበአ |
|  | **ምርመረስ አቂ ሜንት ቅጠንች ገርደበ /Perceived self-efficacy/** |  |  |  |
|  | ማህፀን ምርመረ አቂ ቅጠሜም | ዕተማም | ሜሬራንቹተ | ዕተማምበአ |

**ሴክሺን ሌሆ፡ ምን አናከት አስታ ካለቱተ ጡዳ ጠዕመንቸተ**

| **601** | ማህፀን ሉድ ከንሰርነ ምርመር ተኔ ምንዕኔ አኑ ሀሳዊሽ ከሰንዶ? | 1. አ ሃሳዊሽ ከሼእ 2. ሀሳዊሽ ከሰበአ |  |
| --- | --- | --- | --- |
| **602** | ማህፀን ሉድ ከንሰርነ ምርመር ተኔ ምንዕኔ አኑ ሃሳዊ ፈቃደኛንዶ? | 1. አ ፈቃደኛ 2. ፈቃደኛበአ |  |
| **603** | ማህፀን ሉድ ከንሰርነ ምርመር ተኔ ምንዕኔ አኑ ፋሬእሴኒ ድክሴኒ ከሰንዶ? | 1. አ ፋሬእሽ ከሰ 2. ፈሬእስምበአ |  |
| **604** | ማህፀን ሉድ ከንሰርነ ምርመር ተኔ ኪዕኔ ምርጨ አብሴን ከሰንዶ? | 1. አ አብሰኗ 2. ኢ ምርጨ አብሱምቧ |  |
| **605** | ምንዕኔ አኑ ክዕኔን በርገሜን ማህፀን ምርመሪ መሪ ፈቃደኛንዶ? | 1. አ ፈቃደኛ 2. ፈቃደኛበአ |  |

መረጀ መጠኖ መንች ሱዕመ __________________________________ ፊርመ.___________ በር _________

ካዕለኖ መንች ሱዕመ _____________________________________ ፊርመ. ____________ በር ________

**አኔክስ 5 ፈይመ ሮሸተ አሴኖ ብሮሼረ /ከምባትሰ ላጋን/**

**“ማህፀን ሉድ ከንሰር ምርመረ አቁ ሆለመ ሜንት ፎላከተ ፈዕሰኖ”**

ሜንት ደጉ በርጊ፣ ጡደንቸ መክሲሀ ምርመረ አቅቱንተ ካዕሊሃ ሮሸተ አሲ ቅጠሜ ሮሸ ብሮሼረ

**ወነ ሴክሺነ**

**ማህፀን ሉድ ከንሰሩ መሃን?**

ማህፀን ሉድ ከንሴሩ ማህፀን ሉድ ማላን ሌአኗ ካንሴረ፡፡ ማህፀን ሉደ የመንታ መኖመት ሜንት ፎል እሪች ኬኤን ጪለት አፉዕልታ ማህፀን አዝ አጌኗ ጎጨ፡፡ ጎኒን ኦሰዕን ህጋ መን ፓፕሎም ቨይረሰ የኖ ሆለሙ ሀገረት ዮ እሌን ለልቱምቡ ጀርማከት ማህፀን ሉድ ከንሴሩ ቆጨሙንተ ቆደሰ አለጵታእ፡፡

**ቴሱ ዮ ሞስሲ ኬዕማሽመተ፣ ሞስነ ሬህ ዱዱቡ**

ቶጴ አዜን ወጋን ወጋን 6,300 ሀሮ ማህፀን ሉድ ከንሴር ሞሳኑሁ 4,884 ማህፀን ሉድ ከንሴር ሞሲን ዋልታ ሬሁት ቆጨንታእ፡፡ ኩን አሞ ሜንቱ ጥዲኒ ሺኒ ባዶንተኔ ለንክ ከንሴረ እክ ኬዕ መዘገበሙንተ አሴእ፡፡

**ለንክ ሴክሺነ**

**ማህፀን ሉድ ከንሴር ሞሶን ኡጅታ ጠዋከተ**

ከኒች ወሮዲን ዮሩ ማህፀን ሉድ ከንሴር ሞሶን ኡጅታታ ጠዋከ፡- መን ፓፕሎም ቫይረስ ሞሱን ኡቡ፣ ሄቸ ዶላን ሆለመተ ጎን ጃላከተ አፉ፣ ጎኒን ኦሰዕን ዋልታ ማህፀን ሞሳከት ቆጨሙ፣ ቱምቤኡ ዊሊሹ፣ 18 ኡሙሪች ብርሴን ጎኒን ኦሰኡ ጀመሩ፣ 18 ኡሙሪች ብርሴን ነፍሰ ጡረተ እሁ፣ ሆለመተ ጪለት እሉ (›, 4)፣ አሞ እሉ ከመኖ ክኒነ ሆለመተ ጄቹተ አቁ፡፡

**ማህፀን ሉድ ከንሴር ምልክታከታ ባሰ ገርደበሀ**

ከንሴር ገርደበ እለን ቀጤ ሞስ ምልክተ ሜጡረ ጡድሰኖበአ፡፡ ጠው እኮዳ ወነ ገርደባን ዮ ሳተ ጡድሰኖ ምልክታከት ከሮዕታ፡- አገን ወግ ሜሬሮን ቀቀው ቄጉ ፉለኖ፣ ጎኒን ኦሰኤመ አኒች ዘኪን ቄጉ ፉለኖ፣ በተኤ ፈሳሹ ማህፀኒች ፉለኖ፣ መመቴ አሞ ቦቢ ደንደኖ፣ ማህፀን ጥደት ሆጎደ አሞ ጎኒን ኦሰኤመ ጄቹተ ጥደት መጮጨንታኡ፡፡ ማህፀን ሉድ ከንሴሩ ቃር ኬዕ ባሰ ገርደበ እሌ ጃተ ኬዕማሹ ምቅችነ ጡንች ሞሱ፣ ሙሉ ሁጅተ ኡርሱ፣ ቄጉ ጌኡ፣ ቄጉ ፉሉሁ ማህፀን ግድግዱ ዘረም ጠላዕለ ፉሊ ደንደኖ፡፡

**ማህፀን ሉድ ከንሴረ ከዕመሜኖ ወቃከተ**

ማህፀን ሉድ ከንሴሩ ሜንቶን ቆጨንታ ከንሴር ሞሳካች ከዕሚ ደንዴኖ ከንሴሪች መቶት፡፡ ጄቾን ክትባት አቂኒ ማህፀን ሉደ መረመረሚኒ ሞሱስ ቆጨሙምቦገ ከዕሚ ደንዴኖ፡፡ 14 ወጌ ሜሴላካ 6 አገን አነኖማን አሴኖ ለሙ ክትባቱ ሞሱስ ቆጨሙምቦገ ከሚ ደንደኖ፡፡ ክትባትስ አቂች ብሬ ጎኒን ኦሰኡ ጀመርቶ ሜሴላካ ካዕሉ ሆጊ ደንደኖ፡፡

**ሰክ ሴክሺነ**

**ማህፀን ሉድ ከንሴር ምርመረ አቅ ካለቱተ**

ጄቾን ጄቾን ማህፀን ሉድ ምርመረ አቁ ማህፀን ሉድ ከንሴረ ከዕመሚ አብሽ ካዕለኗ ወቃ፡፡ ምርመሩስ ከንሴሩ ቆጨመኖ አኒች ብሬን ማህፀን ሉዳን ሌአ ጠዋከተ ደጊሃ ህክምነ ብርሴን አቂሃ ካዕለኖ፡፡ ህኩን አሞ ሞሱስ ቆጨመኖበ ከመኖ፡፡ ወሎ መኖመን ብን ይ ህገኖ ከንሴሩ ሆለመተ ጄቹተ ደገመኖ ምርመረ ጄቾን ጄቾን አቅቱምቦ ሜንት አሌኔት፡፡.

**ምርመረ አቅታ ሜንቱሃ አቄኖ ጄቹታ**

ማህፀን ሉድ ከንሴር ምርመረ አቅቱንተ ሰዜኖ ሜንቱ ከኒች ብሬ ጎኒን ኦሰዕ ከሳሪሃ ኡሙሩሰ 30-49 ወጌ አዜን ዮሃርሃት፡፡ ሁንዱንኩ ሜንቱ ሄቸሰ ዶላን ኮች ኮች ዬደ መቴ መረመረም መብቱ ዮሰ፡፡ ጠው እኮዳ መትት ሜንትቹት ኦኦንቶ ወጋን ምርመረ አቂ ደንድታኡ፡፡

**ሜንቱ መረመረንቱምቦገ ከንታ ጉፍቻከተ**

ሜንቱ ምርመረስ አቁ ከንታ ዋነስ ጉፍቻከት ምርመረስ አቅ ተኔ ደግ ኮንትት፣ ማህፀን ሉድ ከንሴሩ ሻኗ ይት ሰዊታ ሰዊቱ፣ ማህፀን ከንሴሩ ኤስ አፈኖኤበአ ይት ሰዊታ ሰዊቱ፣ ብርሴኑንተ ሀሳ ሆለመት ጠዋከት ሄኡ፣ ብር ኮንትት፣ ምርመራንተስ ከንሴሩስ ደገሞደ ሀትተ እሃም ይት ዋጂታጉ፣ ደገሞደ ዋለኖ ሳለሃ ቱሩሃ ዋጁ፣ መቴምኑሁ ጄቹት በጩሁ፣ ሞስስ ምልክቱ በጩ፡፡ ሰዘነስ አነንት አነንት ሜንትቹት ዮ ዱዱቦኒ ከመኖሴ ጠዋካኒ ቀርሰሙ ሀስሰኖ፡፡

**ሾልክ ሴክሺነ**

**ምርመርስ ውጤት ሂረቱተ**

ቶጴ አዜን ዮ ምርመርስ ሱዕሙ ቪአይኤ ያመመኖ፡፡ ኬሚካለ ቡሬን ቀወ ደቂቂች ዘኪን እሌን ጡዴን ደኖተ ዘድ፡፡ ምርመርስ ውጤቱ ፖዛቲቨ ዩ ማህፀን ሉዳን ከንሴረ ቆጭታ ጠዋከት ሌአዮ ዮአ፡፡ ምርመርስ ውጤቱ ንጌቲቨ ዩ ማህፀን ሉዳን ከንሴረ ቆጭታ ጠዋከት ሌዕምበአ ዮአ፡፡ ኩስ ብርተ ለቅ ዮ ዱዱቡ ኩለኖበአ፡፡

**ህክምን ዱዱቡ**

ቪአይኤ ምርመራን ማህፀን ሉዳን ከንሴረ ቆጭታ ጠዋከት ለልቶደ ሜንትቾሀንስ ክርዮቴራፒተ ዬኖ ህክምነ አሴኖ፡፡ አበ ገርደበ ኢሌ ከንሴሩ ለሌደ ገርደብስ ሁኔትጊን ዘብ ህክምነ፣ ዘሬን ጎቡ ሆጎደ ጨረሪን አከሚ ደንዴኖ፡፡ ህከኔን በርጌን አሞ ሞሱስ ኤበኖ ጥደታ ሲቃይሀ ከሚ ዶእሰኖ ዘቡ አሴኖ፡፡

**ምርመርስ ወጭታ ለለኖ ቤቹሃ**

ማህፀን ሉድ ምርመሩ ግዘ ባቴኑዕነን ጠለ አሴኗ ምርመረ፡፡ ከ ጄቾን ዱራሜ ከተማን ዶ/ር ቦጋለች ገብሬ ሆስፒታላኒ ሙዱል ወነ ገርደብ ሆስፒታላኒ ደገመኖ፡፡ ካለቱስ ሁጄ በራካን ሰኒች ሀርቡ እልነቀጤ ሁጄ ሰዓታን አሰመኖ፡፡

**ሜንቱ ምርመረ አቅቱንተ ምን አናከ ተሳትፉት አታ ካለቱተ**

አእኑ ደጌነንተጋንከ ኒ ባድ ገልቴን ቤተሰብ አዝ ጠዋከ አሌን ውሳንተ አስ ቆዱ ምን አናከ አንጋን ዮሃ፡፡ ምን አማከት አሞ አናከት ወሰንቶ ጠዋከተ ሁጄ አሌን ሆስሳበጋን አብስ መብቲሀንሰ ጠዋቱምቧና ጠዕምቱምቧኒ፡፡ አሞ አማከት ቆጨንቸን ኬእሽ ማህፀን ሉድ ከንሰሪን በርግ ቀወሪን ሆለመ ሞሲ አሌን ደጌኑዕነን ኡብ ጎደአንታሃ፡፡ ምን አማከት ምን አናካች ጎኒን ኡሬን ድክሱሃ ደናምተ ላገታ ሀሳሃ፡፡ ማህፃን ሉድ ከንሰሩ ሜጦመ ሄእ ሀዋከኒ ወጬ ሀዋኒ አግሰኖተኔ አናከንታ ቤተሰበንካ አበ ጉዳቴን ኡጀኗ፡፡ አናከት ካዕልቱምቦች አማከት ምርመረ አቁ አብሽ ኬዕመኖሰ፡፡ ህኩን አሞ ሞሱስ ጄቾን ደጌኑምቦገ ጉፍቹ እሀኖተኔ ሬሆ አሌን ኡጂ ደንደኖ፡፡ ህከንተኔ ክእኔ ምን አመት አእኑ ድክሴናን ፈሬእሴናኒ ሀኪም ምን መሬን ምርመረ አቁ ሀስሰኖሰ፡፡

*ምርመረ ጄቾን አቁ ሞሱስ ከዕሚ ፈኔኗ ቁልፈ፣ አሞ ኡሙሩሰ 30 አሉዱሃ እኮ ሜንቱ ሞሶንተስ አብስ ኡብ ደንድታሀ፡፡*

*ከን ሴሬኬትስ ተኔ ደቀሚ ሀሴንተደ: ሳሙኤል ዮሐንሴን ከ ስልክ ቁጥሪን ደቀንቴነተ (ስ.ቁ: 0912009150)*

Annex 4 CV of Principal Investigator

| SAMUEL YOHANNES  Telephone: Office: +2510465552315, Mob. : +2510912009158/+2510932551100  P.O.Box: 159 Hosanna, Ethiopia  Email: [contactsamijohn@gmail.com](mailto:contactsamijohn@gmail.com) | | | |
| --- | --- | --- | --- |
| Personal Identification | | |  |
| Date of Birth | | | August 11, 1975 GC |
| Sex | | | Male |
| Marital Status | | | Married |
| Nationality | | | Ethiopian |
| Career Objective | | |  |
| To make use of my potential as to the maximum level of my academic preparation & attain advancement in my professional achievement | | | |
| Summary of Qualification | | |  |
| I have MPH degree in Reproductive health from Jimma University, BSc degree in Public Health from Dilla University and Higher diploma in reflective training and teaching skills from Dilla University. I have attended and completed medium term online training course in sexual and reproductive health research in 2016 from Geneva Foundation for Medical Education and Research. | | | |
| Education background | | | |
| 2010 | | MPH, Reproductive health Jimma University, Jimma, Ethiopia | |
| 2000 | | BSc, Public Health Dilla University, Dilla, Ethiopia | |
| 2011  2016 | | Diploma in Reflective Training and Teaching Dilla University, Dilla, Ethiopia */Higher Diploma Program/*  Sexual and reproductive health research training  Geneva Foundation for Medical Education and Research | |
| Professional Work Experience | | | |
| Sept.2000 –  Oct. 2005 | *Clinician and technical team coordinator* Adis Hiwot and Abomsa Health centre | | |
| Nov. 2005 to  Nov. 2006 | *Disease prevention and control expert (Merti Woreda Health Office)* | | |
| Dec. 2006 to  June 2007 | *Instructor*  Hosanna College of Health Sciences | | |
| June 2007 to  Sept. 2009 | *Academic and research vice dean* Hosanna College of Health Sciences | | |
| April 2009 to  Sept. 2009 | *Dean of the College (delegated)* Hosanna College of Health Sciences | | |
| Sept 2009 to  June 2010 | Post graduate studies in Jimma University (working with postgraduate academic duties including research activities) | | |
| February 2011 to April 2013 | *Health extension upgrading program coordinator*  Hosanna College of Health Sciences | | |
| April 2013 to  February 2014 | *Instructor, Hosanna College of Health Sciences* | | |
| February 2014 to Jan 2018 | *In-service training coordinator, Hosanna CHS*   - Coordinating different in-service trainings in the college | | |
| Feb 2018 to present | *Instructor and Researcher, Hosanna College of Health Sciences* | | |
| Publications and Research Experiences | | | |
| Publications and research experiences | 1. Duration and determinants of birth interval among women of child bearing age in Southern Ethiopia. <http://www.biomedcentral.com/1471-2393/11/38> 2. Assessment of knowledge, attitude and practice about malaria and ITNs utilization among pregnant women in Shashego district, southern Ethiopia. <http://www.malariajournal.com/content/pdf/s12936-015-0755-7.pdf> 3. Prevalence of smear positive pulmonary tuberculosis and associated risk factors among prisoners in Hadiya Zone prison, Southern Ethiopia. <http://bmcresnotes.biomedcentral.com/articles/10.1186/s13104-016-2005-7> 4. The association between malaria and malnutrition among under five children in Sheshogo District, Southern Ethiopia. <https://www.ncbi.nlm.nih.gov/pmc/articles/PMC5234126/> 5. Knowledge, attitude and practice for cervical cancer prevention and control among women of childbearing age in Hossana Town, Hadiya zone, Southern Ethiopia: Community-based study. <https://doi.org/10.1371/journal.pone.0181415> 6. Utilization and Factors Affecting Adolescents and Youth Friendly Reproductive Health Services among Secondary School Students in Hadiya Zone, Southern Nations, Nationalities and Peoples Region, Ethiopia <https://www.omicsonline.org/open-access/utilization-and-factors-affecting-adolescents-and-youth-friendly-reproductive-health-services-among-secondary-school-students-in-h-96951.html> 7. Health seeking behavior and its determinants for cervical cancer among women of childbearing age in Hossana Town, Hadiya zone, Southern Ethiopia: community based cross sectional study. <https://doi.org/10.1186/s12885-018-4203-2> 8. Hypertension and its associated factors in Hosanna town, Southern Ethiopia: community based cross‑sectional study. <https://doi.org/10.1186/s13104-018-3435-1> 9. Adolescent-parent communication regarding SRH issues among high school students of Hadiya zone <https://doi.org/10.1186/s12887-018-1388-0> 10. Proximate determinants of fertility in Ethiopia; an application of revised Bongaarts model <https://doi.org/10.1186/s12978-019-0677-x> 11. Utilization of Integrated Community Case Management and Its Factors in Southern Ethiopia: Facility Based-Cross-Sectional Study <https://doi.org/10.1155/2021/8835804> 12. Accomplished project on “Effective implementation research skill (EIRS) training for the prevention, control and elimination of malaria, tuberculosis and neglected tropical disease training” 13. Health seeking behaviour & its determinants among residents of Hosanna town 14. Practices of essential hygiene action among lactating mothers during complimentary feeding and associated factors in Hosanna town, Hadiya zone, SNNPR, Ethiopia 15. Engagement of health professionals in the prevention and control of MDR-TB among health professionals in Hadiya Zone: Qualitative study | | |
| Computer and software skills | - Basic knowledge and skills on MS offices, SPSS and Epi-Info | | |
| Other experiences | - I participated in the development and preparation of Strategic plan and Balanced Score Card, research and teaching material preparation guideline, students’ field practice guideline, review of college legislation, In-service Training internal Guideline, exam preparation and administration guideline, SNNPR health sciences colleges forum regulation, retooling implementation guideline. | | |
| Trainings |  | | |
| May-November 2016 | - Training Course in Sexual and Reproductive Health Research organized by Geneva Foundation for Medical Education and Research (GFMER) | | |
| May 2016 | - The evidence based management of post-partum haemorrhage online course organized by University of Oxford and GFMER | | |
| June 2016 | - Obstetric fistula an e-learning course organized by Global Health Network | | |
| June 2016 | - Maternal Health infections an e-learning course organized by Global Health Network | | |
| December 2016 | - Leadership, Management and Governance (LMG) training organized by FMOH in collaboration with MSH-LMG project | | |
| Nov 21-26,2016 | - Grant Development and Management training organized by FMOH in collaboration with ICAP-Columbia University | | |
| August 29- Sep 3, 2016 | - National In-service Training Management organized by FMOH and ICAP-Columbia University | | |
| January 12-30, 2004 | - HIV/AIDS voluntary counselling and testing training course organized by Oromiya Health Bureau and Family Health International Ethiopia | | |
| June 10-12, 2015 | - Blended Comprehensive HIV Testing and Counselling organized by FMOH in collaboration with ICAP-CUE | | |
| August 13- Sept 7, 2007 | - Curriculum & teaching/Training and Learning Materials Development on Health Sector Occupations Organized by FMOE in Collaboration with FMOH | | |
| June 27- July1, 2011 | - Effective teaching skill training organized by MCHIP in collaboration with FMOH and USAID | | |
| Sept 19-24, 2011 | - Clinical Teaching Skills and Student performance Assessment organized by MCHIP in collaboration with FMOH and USAD | | |
| Nov.6- 19, 2011;  Jan.22- Feb. 4, 2012 | - Curriculum development for Ethiopian Health sciences colleges organized by FMOH and FMOE | | |
| April 19-30, 2004 | - Case management course on Integrated Management of New Born and Child hood Illnesses organize by Oromiya Health Bureau and Jimma U. | | |
| May 31- June 5, 2004 | - Facilitation Skill course training on Integrated management of Childhood illness organized by OHB and Jimma University | | |
| June 9^th^ to 10^th^, 2005 | - Facilitating training on the new malaria diagnosis and treatment guideline organized by Arsi Zone Health Department and merlin | | |
| February 12-16, 2007 | - Training on syndromic management of sexually transmitted infections (STIs) organized by SNNPR regional Bureau and CDC Ethiopia | | |
| December 22-27 &  March 5 to 10, 2007 | - Facilitating training on integrated management of new-born and childhood illnesses organized by save the children Ethiopia, and SNNPR RHB | | |
| March 19-24 and  June 18-23, 2012 | - Facilitating training on integrated management of new-born and childhood illnesses organized by JHU, and SNNPR RHB | | |
| Jan25-Feb.21, 2016 | - Facilitation of Effective implementation research skills training for researchers organized by Hosanna College of Health Sciences with financial support from WHO-TDR | | |
| April 4-6, 2016 | - In-service training Data Base Management for health professionals, organized by FMOH in collaboration with Jhpiego Ethiopia | | |
| Nov. 14-17, 2014  Feb.28-Mar3, 2015 | - Facilitation of Effective Teaching Skills training organized by IST and HSEDC of Hosanna College of Health Sciences | | |
| May 02-19, 2006 | - TOT course organized for accelerated implementation of Health service extension program on EPI, RH, IMNCI, ENA, Malaria, Hygiene and sanitation, HIV/AIDS, IEC, Monitoring and Evaluation organized by FMOH and SNNPR RHB in collaboration with UNICEF | | |
| July 15^th^ to 19^th^, 2003 | - Training on PHC facility management organized by Arsi Zone Health Department and Medical Emergency Relief International (merlin) | | |
| August 16^th^ -27^th^ 2004 | - Facilitating training of community malaria agents organized by Arsi Zone Health department and Medical Emergency Relief International. | | |
| Febr.28^th^ to March 10^th^, 2000 | - Skill development course on emergency surgery, resuscitation, orthopedics, trauma and obstetrics organized by Dilla College of Health Sciences in collaboration with Tropical Health and Education Trust, UK | | |
| Languages | - I have the skills to speak and write Kembatisa, Amharic & English | | |
| Professional Membership | - Ethiopian Public Health Association; Member since May, 2006 | | |

Annex 5 CV of Advisors

1. First advisor (Muluemebet Abera Wordofa, PhD, Associate Professor of RH)
2. Second advisor (Tefera Belachew Lema, PhD, Profesor of Nutrition)
