# Supplementary material for: Effectiveness of couple education and counseling on knowledge, attitude and uptake of cervical cancer screening service among women of child bearing age in Southern Ethiopia: A cluster randomized trial protocol
Source: PLoS One. 2022 Jul 21;17(7):e0270663. doi: 10.1371/journal.pone.0270663 (PMC9302843; doi:10.1371/journal.pone.0270663)
Supplement: Attachment — Submitted filename: PONE-D-21-32347_reviewer .pdf [file pone.0270663.s006.pdf]

PLOS ONE

# Effectiveness of Couple Education and Counseling on Knowledge, Attitude and Uptake of Cervical Cancer Screening Service among Women of Child Bearing Age in Southern Ethiopia: a cluster randomized trial protocol

--Manuscript Draft--

| | |
|---|---|
| **Manuscript Number:** | PONE-D-21-32347 |
| **Article Type:** | Study Protocol |
| **Full Title:** | Effectiveness of Couple Education and Counseling on Knowledge, Attitude and Uptake of Cervical Cancer Screening Service among Women of Child Bearing Age in Southern Ethiopia: a cluster randomized trial protocol |
| **Short Title:** | Effectiveness of Couple Education and Counseling on Knowledge, Attitude and Uptake of Cervical Screening |
| **Corresponding Author:** | Samuel Yohannes Ayanto<br>Hosanna College of Health Sciences<br>Jimma, ETHIOPIA |
| **Keywords:** | Cervical cancer, cervical cancer screening, effectiveness, couple education and counseling, cluster randomized trial |
| **Abstract:** | Abstract<br><br>Background<br><br>Cervical cancer occurred nearly in 570 000 women and 311 000 women died from the disease worldwide in 2018. Of the new cases diagnosed globally in 2012, approximately 85% of the burden took place in low- and middle-income countries. Human Papilloma virus is the necessary cause for the development of cervical cancer and the majority of these infections resolves naturally but progress to precancerous lesions whenever there is persistence and delay in treatment. Majority of the cervical cancer cases, over 80% in sub-Saharan Africa including Ethiopia, have been detected at a late stage mainly due to poor early preventive measures. Therefore, utilization of early preventive measures could increase timely detection and treatment of precancerous changes and significantly reduce morbidity & mortality due to advanced disease.<br><br>Methods<br><br>In this interventional study we will randomly assign 16 clusters (kebeles) in to the intervention and the control arm using block randomization. The study employed a cluster randomized controlled trial. Women are eligible to participate in this study when they satisfy certain eligibility criteria; being in the age range of 30-49 years, no history of hysterectomy, did not receive cervical cancer or pre-cancer treatment and non-pregnant. Home based couple education and counseling will be provided to the eligible participants within the intervention group, while the control group receives standard of care. Base line and end line surveys will be completed by interviewing 288 eligible women to evaluate the effect of couple education and counseling on the knowledge, attitude and cervical cancer screening uptake. Generally the intervention lasts for six months. The results of baseline & end line surveys will be compared between the groups to determine the effectiveness of the intervention. Blinding is not possible due to the clustering of the trial arms.<br><br>Discussion: Findings of the study will inform the regional or national scale up of the intervention modality to achieve the screening targets set by the Ethiopian government and world health organization. |
| **Order of Authors:** | Samuel Yohannes Ayanto |
| | Tefera Belachew |
| | Muluemebet Abera Wordofa |

*Powered by Editorial Manager® and ProduXion Manager® from Aries Systems Corporation*

| Additional Information: | |
|---|---|
| **Question** | **Response** |
| **Financial Disclosure**<br><br>Enter a financial disclosure statement that describes the sources of funding for the work included in this submission. Review the submission guidelines for detailed requirements. View published research articles from *PLOS ONE* for specific examples.<br><br>This statement is required for submission and **will appear in the published article** if the submission is accepted. Please make sure it is accurate.<br><br>**Unfunded studies**<br>Enter: *The author(s) received no specific funding for this work.*<br><br>**Funded studies**<br>Enter a statement with the following details:<br>• Initials of the authors who received each award<br>• Grant numbers awarded to each author<br>• The full name of each funder<br>• URL of each funder website<br>• Have or will the sponsors or funders play any role in the study design, data collection (when applicable) and analysis, decision to publish, or preparation of the manuscript?<br>• **NO** - Include this sentence at the end of your statement: *The funders had and will not have a role in study design, data collection and analysis, decision to publish, or preparation of the manuscript.*<br>• **YES** - Specify the role(s) played.<br><br>* typeset | This research will be carried out by financial support from Jimma University. Jimma University had no role over the design, data collection, management and analysis, report writing, interpretation and the decision to submit the report for publication process. The research has been internally funded by Jimma University and SYA received the fund for the study. Its official website is https://ju.edu.et/. |
| **Competing Interests**<br><br>Use the instructions below to enter a competing interest statement for this submission. On behalf of all authors, disclose any competing interests that could be perceived to bias this work—acknowledging all financial support and any other relevant financial or non-financial competing interests. | The authors have no competing interests |

| | |
|---|---|
| This statement is required for submission and **will appear in the published article** if the submission is accepted. Please make sure it is accurate and that any funding sources listed in your Funding Information later in the submission form are also declared in your Financial Disclosure statement.<br><br>View published research articles from *PLOS ONE* for specific examples.<br><br>**NO authors have competing interests**<br><br>Enter: *The authors have declared that no competing interests exist.*<br><br>**Authors with competing interests**<br><br>Enter competing interest details beginning with this statement:<br><br>*I have read the journal's policy and the authors of this manuscript have the following competing interests: [insert competing interests here]*<br><br>* typeset | |
| **Ethics Statement**<br><br>Enter an ethics statement for this submission. This statement is required if the study involved:<br><br>• Human participants<br>• Human specimens or tissue<br>• Vertebrate animals or cephalopods<br>• Vertebrate embryos or tissues<br>• Field research<br><br>Write "N/A" if the submission does not require an ethics statement.<br><br>General guidance is provided below. Consult the submission guidelines for detailed instructions. **Make sure that all information entered here is included in the** | The ethical approval statement has been attached as a supplementary information |

Powered by Editorial Manager® and ProduXion Manager® from Aries Systems Corporation

| | |
|---|---|
| **Methods section of the manuscript.**<br><br>**Format for specific study types**<br><br>**Human Subject Research (involving human participants and/or tissue)**<br>• Give the name of the institutional review board or ethics committee that approved the study<br>• Include the approval number and/or a statement indicating approval of this research<br>• Indicate the form of consent obtained (written/oral) or the reason that consent was not obtained (e.g. the data were analyzed anonymously)<br><br>**Animal Research (involving vertebrate animals, embryos or tissues)**<br>• Provide the name of the Institutional Animal Care and Use Committee (IACUC) or other relevant ethics board that reviewed the study protocol, and indicate whether they approved this research or granted a formal waiver of ethical approval<br>• Include an approval number if one was obtained<br>• If the study involved *non-human primates*, add *additional details* about animal welfare and steps taken to ameliorate suffering<br>• If anesthesia, euthanasia, or any kind of animal sacrifice is part of the study, include briefly which substances and/or methods were applied<br><br>**Field Research**<br><br>Include the following details if this study involves the collection of plant, animal, or other materials from a natural setting:<br>• Field permit number<br>• Name of the institution or relevant body that granted permission | |
| **Data Availability**<br><br>Authors are required to make the data underlying their research findings fully available, without restriction.<br><br>If the manuscript reports pilot or preliminary results, the data underlying | N/A - the protocol does not report results |

| | |
|---|---|
| those results must be made available at the time of publication. A Data Availability Statement describing where the data can be found is required at submission. Your answers to this question constitute the Data Availability Statement and will be published in the article, if accepted. When legal or ethical requirements prohibit public data sharing, authors must indicate how researchers may obtain access to the data. See the PLOS Data Policy and FAQ for detailed information.<br><br>**Important:** Stating 'data available on request from the author' is not sufficient. If your data are only available upon request, select 'No' for the first question and explain your situation in the text box.<br><br>For protocols without pilot or preliminary data, authors are strongly encouraged to state how they plan to share research data from their study when it is completed or published.<br><br>Do the authors confirm that EITHER the protocol does not report results OR all data underlying the preliminary results will be made fully available without restriction? | |
| **Describe where the data may be found in full sentences. If you are copying our sample text, replace any instances of XXX with the appropriate details.**<br><br>• If **no pilot data are reported** in the manuscript, please enter the following: *No datasets were generated or analysed during the current study. All relevant data from this study will be made available upon study completion.*<br>• If the manuscript reports pilot data **held in a public repository**, include URLs, accession numbers or DOIs. If this information will only be available after acceptance, indicate this by ticking the | The datasets generated during the study will be made available through the corresponding author upon justified official requests. |

| | |
|---|---|
| box below. For example: *All XXX files are available from the XXX database (accession number(s) XXX, XXX.).*<br>• If the manuscript reports pilot data contained **within the manuscript and/or Supporting Information files**, enter the following:<br>*All relevant data are within the manuscript and its Supporting Information files.*<br>• If neither of these applies but you are able to provide **details of access elsewhere**, with or without limitations, please do so. For example:<br><br>*Data cannot be shared publicly because of [XXX]. Data are available from the XXX Institutional Data Access / Ethics Committee (contact via XXX) for researchers who meet the criteria for access to confidential data.*<br><br>*The data underlying the results presented in the study are available from (include the name of the third party and contact information or URL).*<br>• This text is appropriate if the data are owned by a third party and authors do not have permission to share the data.<br><br>For study protocols with data management or sharing plans, authors are encouraged to briefly describe in their Data Availability Statement how data they generate will be made accessible when the study is completed. For example:<br>• Deidentified research data will be made publicly available when the study is completed and published.<br><br>* typeset | |
| Additional data availability information: | Tick here if the URLs/accession numbers/DOIs will be available only after acceptance of the manuscript for publication so that we can ensure their inclusion before publication. |

Click here to access/download;Manuscript;Cluster Randomized Controlled Trial Protocol.docx

# Effectiveness of Couple Education and Counseling on Knowledge, Attitude and Uptake of Cervical Cancer Screening Service among Women of Child Bearing Age in Southern Ethiopia: a cluster randomized trial protocol

Samuel Yohannes Ayanto[1*], Tefera Belachew[2], Muluemebet Abera Wordofa[3]

[1]Jimma University, department of Population and Family Health, PhD candidate

[2]Jimma University, department of Nutrition and Dietetics, Professor of Nutrition

[3]Jimma University, department of Population and Family Health, Associate Professor of Reproductive Health

[*]Correspondence: contactsamijohn@gmail.com

Sponsor: Jimma University; *Address*: *Tel* +251471111457; *PO Box* 378; *Email*: ero@ju.edu.et

## Abstract

**Background**

Cervical cancer occurred nearly in 570 000 women and 311 000 women died from the disease worldwide in 2018. Of the new cases diagnosed globally in 2012, approximately 85% of the burden took place in low- and middle-income countries. Human Papilloma virus is the necessary cause for the development of cervical cancer and the majority of these infections resolves naturally but progress to precancerous lesions whenever there is persistence and delay in treatment. Majority of the cervical cancer cases, over 80% in sub-Saharan Africa including Ethiopia, have been detected at a late stage mainly due to poor early preventive measures. Therefore, utilization of early preventive measures could increase timely detection and treatment of precancerous changes and significantly reduce morbidity & mortality due to advanced disease.

**Methods**

In this interventional study we will randomly assign 16 clusters (kebeles) in to the intervention and the control arm using block randomization. The study employed a cluster randomized controlled trial. Women are eligible to participate in this study when they satisfy certain eligibility criteria; being in the age range of 30-49 years, no history of hysterectomy, did not receive cervical cancer or pre-cancer treatment and non-pregnant. Home based couple education and counseling will be provided to the eligible participants within the intervention group, while the control group receives standard of care. Base line and end line surveys will be completed by interviewing 288 eligible women to evaluate the effect of couple education and counseling on the knowledge, attitude and cervical cancer screening uptake. Generally the intervention lasts for six months. The results of baseline & end line surveys will be compared between the groups to determine the effectiveness of the intervention. Blinding is not possible due to the clustering of the trial arms.

**Discussion:** Findings of the study will inform the regional or national scale up of the intervention modality to achieve the screening targets set by the Ethiopian government and world health organization.

**Trial Registration**: PACTR, PACTR202108529472385. Registered on 05 August 2021, https://pactr.samrc.ac.za/TrialDisplay.aspx?TrialID=16037

**Protocol version**: October 8, 2021, version 1.

**Key words**: Cervical cancer, cervical cancer screening, effectiveness, couple education and counseling, cluster randomized trial

## Introduction

Human Papilloma Virus (HPV) is the necessary cause for the development of cervical cancer. Nearly all sexually active women are infected with HPV during their lifetime but majority of them naturally resolve within 24 months (1). However, approximately 12% of these acute infections become persistent and can progress to precancerous lesions or invasive cervical cancer over decades, when not detected and treated early (2). The precancerous lesions progress to advanced cancer stages usually within 10 to 20 years. This extended course in the progression of HPV infection to advanced cancerous stage provides an opportunity for the implementation of effective screening programs to prevent the development of cervical cancer through early detection and treatment modalities (1).

In 2012, the global cancer statistics showed that there were an estimated 527,600 new cervical cancer cases and 265,700 deaths worldwide (3). Also, in 2018, cervical cancer occurred nearly in 570 000 women and 311 000 women died from the disease globally (4). Cervical cancer in low- and middle-income countries (LMICs) accounted for approximately 85% of new cases and 87% of deaths that occurred globally in 2012 (5). But in resource-rich countries the incidence and mortality due to cervical cancer were two to four times lower than what had been seen in resource scarce countries. The highest disease burden was demonstrated in southern and eastern Africa (4). All these statistics describes that there have been disproportionately heavy burden of cervical cancer among women in less developed regions of the world (1).

Larger proportions of cervical cancer cases and deaths occurred in Sub-Saharan African countries in 2018. Of the highest regional incidence and mortality rates observed in Africa, Eastern Africa shared the highest mortality rate due to cervical cancer in the year (6). Also studies reported that the highest prevalence of cervical infection with HPV is recorded in Sub-Saharan Africa (SSA) countries (7). Based on the projections made by World Health

Organization (WHO), cervical cancer will be responsible for 443,000 deaths in 2030 globally (8) of which 98% will occur in low income countries, with SSA facing the highest number of deaths (5).

In Ethiopia, in a trend analysis of the cancer registry data, 5293 cervical cancer cases were diagnosed between 1997 and 2012 and accounted for 31.8% of all new cancer cases (9). Also the trend analysis of cancer from 1998 to 2010 in Ethiopia showed that, malignancy involving cervix is among the leading malignancies in the country (10). The estimated number of new cases of cervical cancer was 6047 with age specific incidence rate of 22% that accounts for about 20% of all identified female cancer cases in 2015 (11). According to world’s summary report, about 6,300 new cervical cancer cases and 4,884 deaths due to cervical cancer occur each year in Ethiopia that makes cervical cancer the second-most common, and the second-most deadly cancer in the country among the target group (12).

Cervical cancer incidence and mortality have been considerably reduced in high resource countries during the last few decades. This is mainly due to the implementation of screening packages for the detection of precancerous cervical lesions and HPV infection. The availability of improved treatment options also played their role in this regard (13). However, in low- and middle income countries where access to screening and treatment services is limited, cervical cancer remains a significant public health problem (6).

As a cervical cancer preventive measure, the Ethiopian Federal Ministry of Health (FMOH) in collaboration with the Pathfinder piloted Visual Inspection with Acetic acid (VIA) screening services combined with access to cryotherapy in 2009 for people living with Human Immunodeficiency Virus (HIV). These services have been later on scaled up into public healthcare facilities and standardized with the subsequent development of comprehensive

cervical cancer prevention and control guideline in 2015 (14). Nearly 1% of age-eligible women ever received screening in Ethiopia before the implementation of this guideline (15). But more recent studies reported an uptake of 9.9% –15.5% in selected populations in Southern and Southwest Ethiopia (Yitagesu, Samuel and Tariku, 2017, Dulla, Daka and Wakgari, 2017, Nigussie, Admassu and Nigussie, 2019). Though the progress shows a favorable trend, it is far away from 80% target coverage nationally set for the 30-49 years target population by 2020 (19) which calls urgent public health interventions.

Evidences suggest that the availability and utilization of screening programs combined with effective treatment options lead to a significant reduction in the morbidity and mortality associated with advanced cervical cancer. In Ethiopia, despite the availability of screening services, only few of the eligible women underwent screening for cervical pre-cancer. Moreover studies have not been conducted to determine the effectiveness of locally adapted interventions which could increase cervical pre-cancer screening uptake among eligible women. Our research, therefore, aims to test the effectiveness of leaflet assisted home based couple education and counseling on knowledge, attitude and uptake of cervical cancer screening among eligible women.

## Methods

### Objectives

The objective of this study is to determine the effectiveness of leaflet assisted home based couple education and counseling in increasing the knowledge, attitude and uptake of cervical pre-cancer screening services among eligible women. Two groups of women are compared where the intervention group receives home based couple education and counseling while the control group receives the usual standard of care available in routine service delivery schemes.

## Trial design

This study is a two arm parallel cluster randomized trial that will be conducted in the Southern people regional state of Ethiopia.

## Study setting

The geographic location for our study is Kembata Tembaro and Hadiya zones which are located in the Southern Nations Nationalities and Peoples' Regional State. The total number of age eligible women (30-49 years) for cervical cancer screening in the study zones constitutes 19.2% of women of reproductive age group residing in the zones. Kembata Tembaro zone comprises of 8 districts and 3 city administrations with the total number of 150 clusters or kebeles, the smallest administrative units, of which 9 are located in the city administrations. The zone has one general hospital, four primary hospitals, 33 health centers and 138 health posts. Hadiya zone is administratively organized in to 13 districts and 4 city administrations having a total number of 329 clusters. The zone has one comprehensive specialized hospital, three primary hospitals, 61 health centers and 317 health posts.

The health care of women of child bearing age in general and maternal & child health care services in particular are of the main strategic pillars of the health service delivery efforts in the zones. Cervical cancer prevention and control activities are being integrated in to the routine health care delivery as an important health service package for the women's health. Currently, cervical cancer screening services are being implemented at selected hospitals and health centers of the zones where our research activities will base these facilities.

## Participants' eligibility

The study participants are women of child bearing age who are eligible for cervical cancer screening according to the Ethiopian national cervical cancer prevention and control guideline

(14). Accordingly women aged 30-49 years are targets for cervical cancer screening program and our research will be carried out within this program framework. Women should also satisfy the requirements of legal residency within their respective living quarters for at least six months, have not had received the screening services within the last 5 years, non-pregnant, beyond three months of postpartum, have not had hysterectomy, have not been diagnosed for any gynecological cancer including cervical cancer. These eligible women will be identified through censusing or health post records.

**Recruitment of participants**

Initially, sixteen non-adjacent clusters or kebeles are selected from the two study districts where cervical cancer screening services are currently provided. The clusters are randomly assigned in to intervention and control groups using block randomization technique. Census or health post records will be used to identify all the eligible women for cervical pre-cancer screening in the selected clusters and sampling frame will subsequently be created. Then, simple random sampling technique will be used to select study participants from each cluster for each arm. We will select an equal number of participants from each selected cluster.

Those women who consent to participate in the study will be included and requested to sign an informed consent to ensure voluntary participation. Selected women at each arm will be reached by health extension workers and the data collectors at their home. Once consent is obtained, each participant will be interviewed to complete a baseline survey. The baseline questionnaire includes items on cervical cancer screening knowledge, attitudes, screening experiences, and socio-demographic variables. Similarly end line survey will be conducted at six months.

**Randomization**

The clusters or kebeles are the units of randomization in our study. Initially, sixteen non-adjacent clusters, eight from each district, were identified from the two study districts. We stratified the clusters based on the study districts and created separate list of eight clusters alphabetically for each district. The stratification was done to evenly distribute any known and unknown district level confounders across the study arms. Each cluster was assigned a unique cluster code. Then, the statistician assigned the eight clusters in to two blocks of size 4 according to the order they appeared alphabetically. The statistician randomly selected the randomization sequence of clusters for each block using the sealed lots of the six possible permutations within the block.

The clusters in each block were randomly assigned to intervention and control arms according to the randomization sequence evident by the selected permutation within in the block for the stratum. We repeated the same process for blocks of clusters in the other stratum. Consequently, four clusters were obtained from each district which formed eight clusters to the intervention arm and eight clusters to the control arm maintaining 1:1 random allocation ratio.

The statistician was made unaware of the actual study arms to mask the knowledge about which group will receive the intervention and which group will receive the usual care. This was achieved by representing the study arms and clusters by confidential codes. Health extension workers will carry out identification of eligible participants' for the study and subsequent intervention administration activities. Assessors will also be made blind to the nature of clusters with respect to intervention administration.

**Contamination reduction**

When women from intervention kebeles join those women in the control kebeles during social occasions including funeral, wedding, marketing etc., there will be a possibility to contaminate the intervention messages through informal discussions of ideas regarding the intervention

activities. As a result individuals from control kebeles may gain some intervention messages which may affect the intervention effect unfavorably. To reduce such an undesirable effect of information contamination, a buffer zone has been used to create non-adjacent clusters of the two study arms using clusters that do not take part in our study.

## Intervention description

The full version of our proposed intervention modality is brochure assisted home based couple education and counseling followed by formal invitation for cervical pre-cancer screening. All the selected eligible women in the intervention clusters who consented to participate will be provided with the proposed intervention. The woman and her husband will be educated about cervical cancer and its screening and counseled on the importance of screening for cervical pre-cancer. The husband will also be counseled on the importance of support and encouragement to his wife for cervical pre-cancer screening. In addition, the woman will receive formal letter of invitation during the last visit which reinforces key messages of the intervention and importance of getting screened for cervical pre-cancer.

The educational and counseling material is organized to address susceptibility to cervical cancer, its seriousness, the benefits of screening and barriers to screening with the objective to build cues to action and develop woman’s self-efficacy using health belief model. The intervention is organized and designed to bridge the knowledge gap and positively influence women’s belief system related to cervical cancer and its screening. This, in effect would bring positive behavior change among women to use cervical pre-cancer screening services.

The educational brochure consists of four sections. Section one provides general information on the definitions, magnitude and incidence of the disease. Section two gives information and knowledge of risk factors, signs and symptoms, complications, and preventive modalities of the

disease. Section three offers an explanation of the eligibility criteria, screening schedule, benefits and barriers to screening services to encourage participants to adopt positive behaviors. Section four explains the meanings of screening results, available treatment options, cost and location of the service and importance of male involvement in woman's cervical pre-cancer screening uptake.

**Implementation of the intervention**

Each woman will receive a total of three contacts during the intervention period. Accordingly, each eligible woman in the intervention clusters will be physically visited three times at her residential home. During the initial contact, a 45 minutes leaflet guided education & counseling session will be held with the woman to convey information on cervical cancer and its screening and counsel the woman to encourage the uptake of screening service. At the end of the session, discussion will be held with the woman to address any questions and concerns. The woman will also be provided with the educational brochure for further reading at least once per week by themselves or to be read by any literate person within the family or neighborhood. The date of the subsequent follow up visit will be made consensually with the emphasis to attend the session together with her husband.

A follow up visit will be made to each woman one month after the initial visit with the objectives to re-emphasize important points of the material and make encouragement for screening. During this visit both the woman and her husband together will receive key information on cervical cancer and the importance of its screening. The husband will also be counseled on the importance and the way how the woman receives his support and encouragement to get screened for cervical cancer. Additionally any misconceptions related with the information will be

corrected and barriers to screening will be addressed during this visit. Repeated follow up visits will be made in a case of absence of the couples.

Finally, the second follow up visit, which is the last visit of the proposed intervention modality, will be made one month after the first follow up visit to convey key messages about cervical cancer and its screening and address any unresolved concerns related to cervical cancer and its screening. Also, a formal letter of invitation will be granted during this visit for free screening services available in the nearby health facility. During all the visits eligible women will be asked key questions at the end of education and counseling session to check women's comprehension. Also screening preparedness plan will be continuously negotiated with the woman at each visit as an encouragement to get screened for cervical cancer.

The intervention period lasts for six months in two phases. Phase one involves the first three months of active delivery of the proposed intervention at which time intervention will be provided to each woman in a monthly base. The second phase consists of the last three months of the intervention period. During the last three months women will not receive any intervention scheme but left unvisited with the objective to provide a period of rehearsal & translation of their knowledge in to practice. The health extension workers of the respective intervention clusters will be trained on the provision mechanisms of the intervention to couples at their homes. The control group will receive the usual standard of care but will be exposed to the same intervention after the trial period through the routine services delivery schemes [figure 2].

**Implementation of screening service**

Those women who visit the screening facilities will receive the service according to the national guideline. Women will be provided with the specific information how they will access the service delivery location and whom they will contact within the health facility. Accordingly

women who come from the study clusters for screening will be linked to the administrative procedures of the health facility to receive the screening service. A trained health professional will undergo assessment & counseling and conduct the screening procedure using visual inspection techniques according to the guideline. Any woman with positive precancerous screening results will receive treatment immediately after the results become available on the same day visit and be counseled to receive a follow up screening after one year. Those women who are tested negative will be counseled to receive a regular screening service after five years. All women who receive the screening will be registered on the format prepared for study purpose.

**Compliance parameter**

Eligible women in the intervention clusters will be repeatedly visited in a case of absence to resolve the problem of compliance to full intervention package. Despite these efforts, due to different reasons, the eligible women in the intervention clusters may not fully comply with the proposed intervention as per the recommendations within the intervention package. This might exert undesirable effect on the uptake of the screening services. The impact of variability in the compliance to the proposed intervention requirements will be considered during analysis as a dose response function. Therefore, compliance checklist will be used to track the level of women's compliance to our proposed intervention package to account for during data analysis.

**Trial protocol summary**

Table 1 summary of the intervention description

| Content of the intervention | Dosage | Frequency | Duration | Compliance parameter |
|---|---|---|---|---|
| Leaflet guided couple education & counseling | 30-45 minutes intervention | Every month | Two months | No of couples educated & counseled |

| | | | | |
|---|---|---|---|---|
| Leaflet guided couple education & counseling and formal invitation | 30-45 minutes intervention | Once a month | One month | No of couples educated & counseled; and formally invited |

**Primary outcome**

The primary outcome of this study is the completion of cervical cancer screening test within 6 months of the baseline assessment. Participants will be interviewed using the survey questionnaire and tracked via health records review for completing the screening service. The screening proportions will be determined for each study arm at the baseline & end line. The difference in the screening proportions between intervention and control arms will be calculated. Finally, the effectiveness of the intervention will be measured as a difference of the differences in the screening uptake between the study arms.

**Secondary outcomes**

We will also assess the progress of participants' knowledge and attitude by measuring their knowledge and attitude at baseline and end line. The effect of the intervention on participants' knowledge and attitude will be determined and compared between the study arms.

**Participant timeline**

Table 2 Participants' timeline of enrolment, intervention and assessment

| Activity | Month 1 | Month 2 | Month 3 | Month 4 | Month 5 | Month 6 | Month 7 | Month 8 |
|---|---|---|---|---|---|---|---|---|
| Participants Enrolment | | | | | | | | |
| Baseline assessment | | | | | | | | |
| Intervention | | | | | | | | |
| End line Assessment | | | | | | | | |

**Sample size**

We identified current cervical cancer screening proportions from existing literatures to be 15.5% among age eligible women (18) for the control arm. We will expect an absolute difference of 20% increase in screening proportions between the arms with the power of 80% and 5% significance level for one-tailed test which gives the expected proportions of screening to be 35.5% for the intervention arm. We also considered the design effect of 2 to adjust for the loss of variability due to clustering and 5% compensation for incomplete and non-response rates that would happen during data collection. Consequently, we arrived at a total of 288 study participants. We used Gpower software to determine our sample size. The required number of clusters we determined for the study is sixteen clusters.

**Sampling procedure**

Sixteen clusters (kebeles) will be selected from the two study districts where cervical cancer screening service is currently available. Sixteen clusters are taken based on the recommendation that taking fewer subjects from many clusters give better representation of the sample than taking many participants from fewer clusters. The clusters will be randomly assigned in to intervention and control groups. Census will be carried out in the selected Kebeles to identify women who are eligible for screening and to create sampling frame. Then, simple random sampling technique will be performed to select study participants in each arm. We will select equal number of participants from each cluster which consequently leads to selection of 18 participants per cluster. The same sample of participants will be used at the end of the intervention phase, six months later, to measure outcome variables [figure 1].

**Data collection methods**

Data will be collected using structured questionnaire designed for meeting the specific research purpose. The tool has been developed based on the research objectives from relevant literature

sources. The data collection instrument will be pretested to check for its clarity, logical sequence, cultural appropriateness etc. and appropriate modification will be made accordingly. Participants will be interviewed to complete baseline survey after randomization of the clusters and end line survey at the end of the intervention period in six months. Participants will be measured for socio-demographic characteristics, knowledge, attitudes, and cervical cancer screening uptake.

Data will be collected through face to face interview technique contacting each woman at her residential home. Data collectors will go to each selected woman's home physically by carrying all the data collection tools and communicate verbally with the local language to get the required information. Two different teams will be assigned for the intervention administration and data collection for the purpose of masking the intervention from data collectors. Both the baseline and end line data will be collected by the same individuals to ease the data gathering process.

**Confidentiality of the data**

The information we will obtain from the participants will be kept confidential and used only for the purpose of the study. No personal identifiers will be recorded on the information and data recording sheet and only codes will be employed. The completed data checklists will be kept in a secure manner until it will be officially discarded.

**Data management**

Specific cluster and individual codes will be assigned for each of the completed questionnaire. The data will be entered using Epi info version 7.2.4.0. The data will be edited and transported to either SPSS or STATA to carry out the desired statistical analysis.

**Statistical analysis**

Base line screening proportions will be computed after randomization and before the intervention administration to measure the last 5 years screening performances of women in both arms. The

groups will be examined and compared at base line for any statistical differences in terms of base line participants' characteristics and their screening experiences using Chi-square test for categorical variables and independent sample t-tests for continuous variables. A one-sided P value of 0.05 will be used to determine statistical significance.

After the end line survey, proportions of women screened during the trial period will be calculated. We will use paired sample t-test to test knowledge about cervical cancer and cervical cancer screening, within the intervention group by comparing the before and after intervention scores. A similar analysis will be done for the control group to compare before and after intervention scores. Then we will employ the independent-sample t-test to determine the effect of the intervention on knowledge and attitude by comparing participants' scores on knowledge and attitude about cervical cancer and its screening, between the two groups.

Finally, we will use Generalized Estimating Equation analysis technique to test the independent effect of the intervention on the uptake of cervical pre-cancer screening services. Intention to treat approach will be employed to analyze the data.

**Data monitoring**

Data will be monitored throughout the trial period with particular emphasis to baseline assessment, intervention period and end line assessment time. A team of field supervisors will carry out the responsibility of monitoring and auditing the overall trial data.

**Dissemination plan**

The findings of this trial will be disseminated to relevant stake holders in the local community. The findings will also be communicated to the scientific community through publication of the results in the peer reviewed journals.

## Discussion

Cervical cancer screening is among the national maternal health intervention priorities in Ethiopia. Even with free screening services available, majority of eligible women do not use the service as expected. Effective locally adapted strategies to increase the uptake of cervical screening have not yet been tested across the nation. The development of nationally sound effective intervention strategies based on reliable research findings needs to be in place to increase the low coverage of the screening services evident currently. The results of this study could increase the uptake of the existing low cervical pre-cancer screening services utilization. Consequently, determining the effectiveness of leaflet assisted home based couple education and counseling on the uptake of cervical cancer screening with subsequent recommendations may help increase the rates of screening among the eligible group. This intervention strategy may also serve as a bridge in reducing cervical cancer morbidity & mortality among the risk groups.

## Abbreviations

FMOH: Federal Ministry of Health; HBM: Health Belief model; HDI: Human development Index; HIV: Human Immunodeficiency Virus; HPV: Human Papilloma Virus; LMICs: Low and Middle Income Countries; SSA: Sub Saharan Africa; TASH: Tikur Anbesa specialized hospital; VIA: Visual Inspection with Acetic acid.

## Ethics approval and consent to participate

This study has been approved by the IRB of Jimma University; that is Jimma University Institute of Health IRB, with approval number: IHRPGn/355 on 16/7/2021. Any major amendment in the protocol will be communicated to the IRB for approval. Each participant will sign consent before data collection by the help of the data collectors using the consent format prepared for the research purpose and its copy will be issued for the participant.

## Consent for publication

Not applicable.

## Availability of data and materials

The datasets generated during the study will be made available through the corresponding author upon justified official requests.

## Competing interests

The authors declare that they have no competing interests

## Funding

This research will be carried out by financial support from Jimma University. Jimma University had no role over the design, data collection, management and analysis, report writing, interpretation and the decision to submit the report for publication process.

## Authors' contributions

SYA, MAW and TBL designed the trial. SYA drafted the manuscript. MAW and TBL critically revised the manuscript. All authors read and approved the final version of the manuscript

## Acknowledgements

We would like to acknowledge Jimma University for granting ethical approval and fund for the conduct of this research activity.

## References

1. WHO. Comprehensive Cervical Cancer Control. 2nd ed. Guideline. Geneva, Switzerland; 2014. 366–378 p.
2. Adebamowo SN. Persistent low-risk and high-risk human Papillomavirus infections of the Uterine cervix in hiV-negative and hiV-Positive Women. Front Public Heal. 2017;5(July):1–11.
3. Torre LA, Bray F, Siegel RL, Ferlay J, Lortet-Tieulent J, Jemal A. Global cancer statistics, 2012. CA Cancer J Clin. 2015;65(2):87–108.
4. Arbyn M, Weiderpass E, Bruni L, de Sanjosé S, Saraiya M, Ferlay J, et al. Estimates of

incidence and mortality of cervical cancer in 2018: a worldwide analysis. Lancet Glob Heal. 2020;8(2):e191–203.
5. Ferlay J, Soerjomataram I, Dikshit R, Eser S, Mathers C, Rebelo M, et al. Cancer incidence and mortality worldwide: Sources, methods and major patterns in GLOBOCAN 2012. Int J Cancer. 2015;136(5):E359–86.
6. Bray F, Ferlay J, Soerjomataram I, Siegel RL, Torre LA, Jemal A. Global cancer statistics 2018: GLOBOCAN estimates of incidence and mortality worldwide for 36 cancers in 185 countries. CA Cancer J Clin. 2018;68(6):394–424.
7. De Vuyst H, Alemany L, Lacey C, Chibwesha CJ, Sahasrabuddhe V, Banura C, et al. The burden of human papillomavirus infections and related diseases in sub-saharan Africa. Vaccine [Internet]. 2013;31(S5):F32–46. Available from: http://dx.doi.org/10.1016/j.vaccine.2012.07.092
8. WHO. Projections of mortality and causes of death, 2015 and 2030. 2012.
9. Abate SM. Gynecology & Obstetrics Trends of Cervical Cancer in Ethiopia. 2015;
10. Wondemagegnhu T. Pattern of Cancer in Tikur Anbessa Specialized Hospital Oncology Center in Ethiopia from 1998 to 2010. Int J Cancer Res Mol Mech ( ISSN 2381-3318 ). 2015;1(1):1–5.
11. Tessema S, Memirie, Kifle M, Habtemariam, Asefa M, Tefera B, et al. Estimates of Cancer Incidence in Ethiopia in 2015 Using Population-Based Registry Data. J Glob Oncol. 2018;
12. Bruni L, Albero G, Serrano B, Mena M, Gómez D, Muñoz J, et al. Human Papillomavirus and Related Diseases in the World- Summary report. ICO/IARC Inf Cent HPV Cancer (HPV Inf Centre) [Internet]. 2019;(June):307. Available from: https://hpvcentre.net/statistics/reports/XWX.pdf
13. Denny L, de Sanjose S, Mutebi M, Anderson BO, Kim J, Jeronimo J, et al. Interventions to close the divide for women with breast and cervical cancer between low-income and middle-income countries and high-income countries. Lancet [Internet]. 2017;389(10071):861–70. Available from: http://dx.doi.org/10.1016/S0140-6736(16)31795-0
14. FDRE M OH. Guideline for Cervical Cancer Prevention and Control in Ethiopia. 2015;
15. Gakidou E, Nordhagen S, Obermeyer Z. Coverage of cervical cancer screening in 57 countries: Low average levels and large inequalities. PLoS Med. 2008;5(6):0863–8.
16. Yitagesu H, Samuel Y, Tariku L. Knowledge, attitude and practice for cervical cancer prevention and control among women of childbearing age in Hossana Town, Hadiya zone, Southern Ethiopia: Community-based cross-sectional study. PLoS One. 2017;12(7):181–200.
17. Dulla D, Daka D, Wakgari N. Knowledge about cervical cancer screening and its practice among female health care workers in Southern Ethiopia: A cross-sectional study. Int J Womens Health. 2017;9:365–72.
18. Nigussie T, Admassu B, Nigussie A. Cervical cancer screening service utilization and associated factors among age-eligible women in Jimma town using health belief model, South West Ethiopia. BMC Womens Health. 2019;19(1):1–10.
19. FMoH. National Cancer Control Plan 2016 - 2020 of Ethiopia. Dis Prev Control Dir. 2015;(October 2015):83.

# Appendices

Annex I: Information sheet and informed consent form

Dear Respondent:

My name is ____________. I am working for the research project entitled "*effect of couple education on knowledge, attitude and uptake of cervical cancer screening service among women of child bearing age in southern Ethiopia*" as a data collector. The purpose of the study is to determine the effect of couple education on knowledge, attitude and uptake of cervical cancer screening services among women of child bearing age. Participation in this research will benefit individual woman by increasing her knowledge and encourage behavioral change to utilize the screening services. It also gives an opportunity to ask questions and learn more about the topic confidentially after the interview. Currently I am working with the research team of Jimma University to realize such objectives. Now, I am going to interview you & collect information which is required for the research purposes. You are randomly selected to be included in the study as part of the sample population to complete the questionnaire designed by the research team. The information obtained in this study will be used only for research purposes. The information you will provide is helpful to achieve the intended objectives of the study so that you and your community will benefit from. Any information obtained will be kept strictly confidential and will not be exposed to any other body. No personal identifiers will be attached to the information you will provide. Involvement in this study is voluntary and you can drop any individual question or the whole questionnaire at any time without giving a reason. Your refusal to participate or drop out will not produce penalty. However, your participation and contribution in the study is very helpful to come up with important findings to intervene the problem. The questionnaire will probably take 30-40 minutes to complete. For any information you can contact Mr. Samuel Yohannes; phone ______

Do you have any opinion regarding this study? Do you agree to participate in this study?

Yes, continue No, thank you!

**Consent form**

I, the undersigned have been informed, in the language I can understand, and understood the purpose of this particular research project. I have been informed that the information I give will be used only for the purpose of the study; my identity, the information I give will be treated confidentially. I have also been informed that I can refuse to participate in the study, not to

respond to question if I am not interested or stop responding to question at any time in the process. Based on the above information I agree to participate in the research voluntarily.

Participant's Sign_____________ Person in charge of the informed consent, sign ______________

Figure1**.** Recommended content for the schedule of enrolment, interventions, and assessments.*

| | | STUDY PERIOD | | | | | | | |
|---|---|---|---|---|---|---|---|---|---|
| | **Enrolment** | **Allocation** | **Intervention Phase** | | | | | | **Close-out** |
| **TIMEPOINT**** | ***-Month1*** | **0** | ***Mon th$_1$*** | ***Mon th$_2$*** | ***Mon th$_3$*** | ***Mon th$_4$*** | ***Mon th5*** | ***Mon th6*** | ***Month7*** |
| **ENROLMENT:** | | | | | | | | | |
| **Eligibility screen** | X | | | | | | | | |
| **Informed consent** | X | | | | | | | | |
| **Allocation** | | X | | | | | | | |
| ***Baseline assessment*** | | X | | | | | | | |
| **INTERVENTIONS:** | | | | | | | | | |
| ***[Couple Education, Counseling & Invitation*** | | | ◆—— | ——— | ——— | ——— | ——— | ——◆ | |
| ***[Usual Standard of Care]*** | | | ◆—— | ——— | ——— | ——— | ——— | ——◆ | |
| **ASSESSMENTS:** | | | | | | | | | |
| ***[Sociodemography, Knowledge, attitude, screening utilization]*** | | | | | | | | | X |
| ***[Knowledge, attitude, screening utilization]*** | | | | | | | | | X |
| ***[Information & Health service variables]*** | | | | | | | | | X |

*Recommended content can be displayed using various schematic formats. See SPIRIT 2013 Explanation and Elaboration for examples from protocols.

**List specific time points in this row.

Click here to access/download;Figure;Fig 1 Sampling procedures.docx

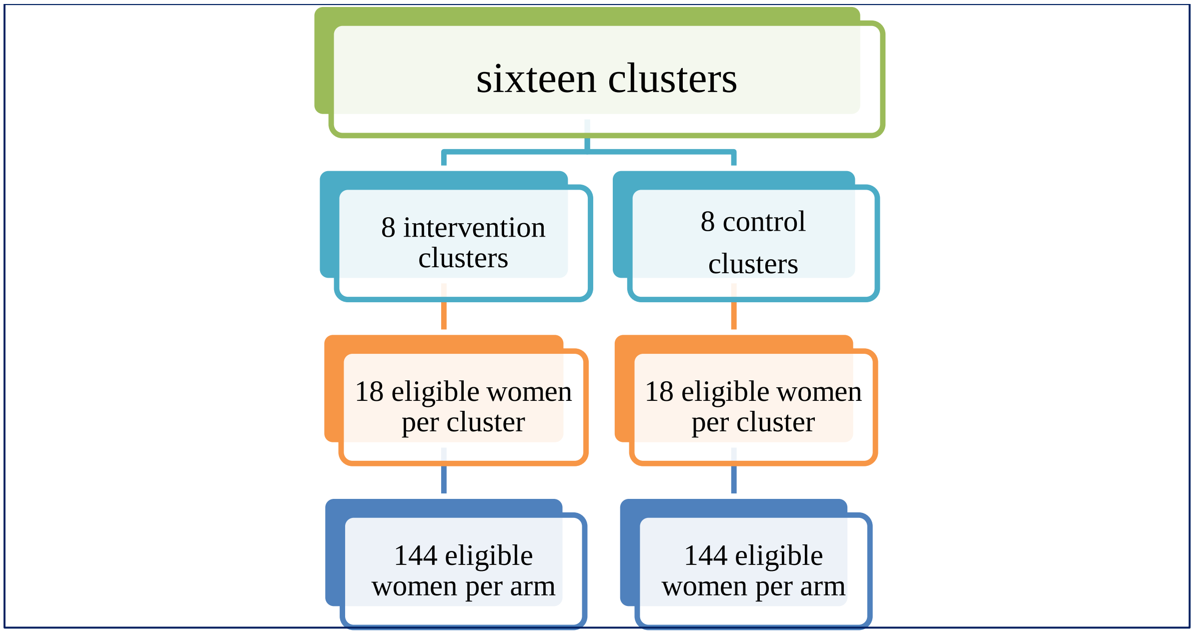


Fig 1 Sampling procedures of the study participants

 Click here to access/download;Figure;Fig 2 Study process.docx

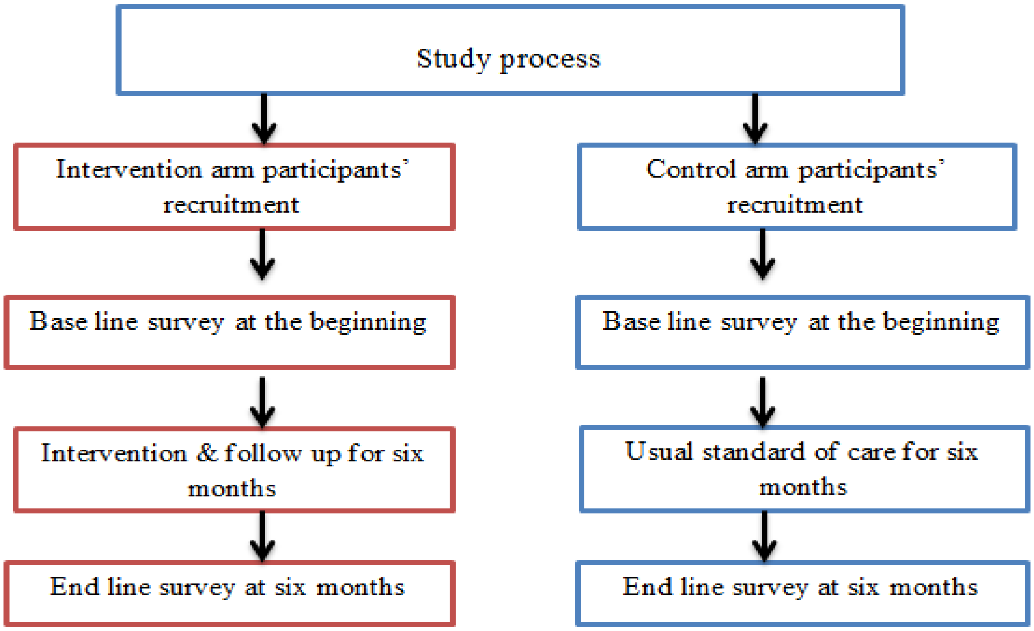


Figure 2 Implementation framework of the study process

Completed SPIRIT Checklist

Click here to access/download
**Supporting Information**
Fig 1 Completed SPIRIT.doc

Click here to access/download
**Supporting Information**
RCT Original Research Protocol_Approved.pdf

Click here to access/download
**Supporting Information**
Ethical Approval Letter.pdf
