# Supplementary material for: Effectiveness of couple education and counseling on knowledge, attitude and uptake of cervical cancer screening service among women of child bearing age in Southern Ethiopia: A cluster randomized trial protocol
Source: PLoS One. 2022 Jul 21;17(7):e0270663. doi: 10.1371/journal.pone.0270663 (PMC9302843; doi:10.1371/journal.pone.0270663)
Supplement: Attachment — Submitted filename: PONE D-21-32347 comments.docx [file pone.0270663.s008.docx]

Line 27 ‘employed’ should be future tense

Line 49 ‘majority of the infections resolve’ or ‘majority of women resolve the infections’

Line 63 ‘these statistics describe’

Line 77 I am not sure what ‘age specific incidence rate’ means. 22% seems very high.

Line 99. Evidence suggests

Line 122. Comprises ~~of~~

Line 160 five thousand
